# β-Ionone enhances the inhibitory effects of 5-fluorouracil on the proliferation of gastric adenocarcinoma cells by the GSK-3β signaling pathway

**DOI:** 10.1371/journal.pone.0309014

**Published:** 2024-09-06

**Authors:** Fa-lin Wang, Xiaoxia Chang, Yuanyang Shi, Tingting Yang, Juan Li, Hongwei Dong, Qi Wang, Shujun Zhang, Jiaren Liu

**Affiliations:** 1 Department of Clinical Laboratory, The Forth Affiliated Hospital of Harbin Medical University, Nangang District, Harbin, China; 2 Department of Clinical Laboratory, Xi’an No. 9 Hospital, Beilin District, Xi’an City, China; 3 Department of Laboratory, Shaoyang Central Hospital, Daxiang District, Shaoyang City, China; 4 Public Health College, Harbin Medical University, Nangang District, Harbin, China; 5 Department of Pathology, The Forth Affiliated Hospital of Harbin Medical University, Nangang District, Harbin, China; Neyshabur University of Medical Sciences, ISLAMIC REPUBLIC OF IRAN

## Abstract

5-Fluorouracil (5-FU) is widely used in the treatment of gastric cancer, and the emergence of drug resistance and toxic effects has limited its application. Therefore, there is an urgent need for safe and effective novel drugs or new therapies. β-Ionone (BI) is found in vegetables and fruits and possesses an inhibitory proliferation of tumor cells in vitro and in vivo. In this study, we investigated whether BI could enhance the inhibitory effects of 5-FU on the proliferation of gastric adenocarcinoma cells and the growth of gastric cancer cell xenografts in a mouse model. The effects of BI and 5-FU alone or their combination on the cell viability, apoptosis, and mitochondrial membrane potential, the cell cycle, and its related proteins—Cyclin D1, and CDK4 as well as PCNA and GSK-3β were evaluated in SGC-7901 cells and MKN45 cells by MTT, MB, flow cytometry and Western blot. In addition, the effects of BI and 5-FU alone or their combination on the growth of SGC-7901 cell xenografts in nude mice were investigated. The results showed that BI significantly enhanced the sensitivity of gastric adenocarcinoma cells to 5-FU in vitro and in vivo, i.e. proliferation inhibited, apoptosis induced and GSK-3β protein activated. Therefore, our results suggest that BI increases the antitumor effect of 5-FU on gastric adenocarcinoma cells, at least partly from an activated GSK-3β signaling pathway.

## 1. Introduction

Gastric cancer is one of the most common digestive system malignancies and the second leading cause of cancer-related deaths worldwide [1]. According to the 2020 Global Burden of Cancer data released by the International Agency for Research on Cancer (IARC), nearly 480,000 (44%) of the new gastric cancer cases in the world in 2020 were in China, and 370,000 (49%) of the global deaths were in Chinese patients [2]. 70–90% of gastric cancer patients are initially diagnosed at an advanced stage and have a poor prognosis. However, chemotherapy is the only option in the continuous treatment for advanced gastric cancer [3].

5-Fluorouracil (5-FU) was introduced in 1957 and is an essential systemic chemotherapeutic agent for treating gastrointestinal cancers. However, the widespread use of 5-FU in clinical treatment has been limited by its insurmountable drug resistance and toxic side effects. When patients with advanced gastric cancer receive single 5-FU therapy, the remission rate is only 21%, and the median survival is ten months [4]. Given the main problems existing in the clinical treatment of chemotherapeutics, phytochemicals can be considered to reduce the resistance of tumor cells to chemotherapeutics [5]. Phytochemicals are biologically active non-nutrient compounds in fruits, vegetables, grains, and other plant foods [6]. Several studies have demonstrated that phytochemicals can increase cancer cells’ sensitivity to chemotherapeutic drugs and overcome their multidrug resistance drawbacks [7, 8]. Combining chemotherapeutic drugs with one or several non-toxic phytochemicals to increase the sensitivity of tumor cells to chemotherapeutic drugs and reduce the toxic side effects of chemotherapeutic drugs is a strategy worth considering.

β-Ionone (BI), a cyclic isoprenoid compound found in many vegetables and fruits, has several biological activities, especially a significant inhibitory effect on the occurrence and development of tumors [9–11]. BI has been shown to inhibit tumor cell proliferation in gastric [12, 13], breast [10, 14], prostate [15], and renal cell malignancies [16] by halting the cell cycle, antioxidation, increasing apoptosis, and inducing autophagy. In addition, BI can also reduce cell migration and invasion in melanoma [17] and prostate cancer [18], affect antioxidant enzymes in animal models of breast cancer [19, 20], and the metastatic ability of gastric adenocarcinoma cells [10]. In our group’s findings, BI showed anti-tumor solid activity in both in vivo and in vitro experiments [10, 21], inhibiting the proliferation and inducing apoptosis of gastric adenocarcinoma cells [12, 14, 22].

Interestingly, BI combined with 5-FU has not been studied in gastric adenocarcinoma. This study aimed to investigate the anti-tumor effects of BI and 5-FU combination on gastric adenocarcinoma cells. These results may provide a theoretical basis for the future development of BI as an adjuvant for chemotherapy in gastric adenocarcinoma.

## 2. Materials and methods

### 2.1 β-Ionone

β-Ionone, 4-(2, 6, 6-trimethyl-1-cyclohexenyl)-3-buten-2-one with >95% purity was purchased from Sigma (USA). It was dissolved in absolute ethanol and diluted to the following concentrations:100, 200, 400, 800, and 1000 μmol/L.

### 2.2 Cell culture

Human gastric adenocarcinoma cells (SGC-7901 cells and MKN45 cells) purchased from the Cancer Research Institute of Beijing (China) were cultured in RPMI 1640 (Gibco) medium, supplemented with calf serum 100 ml/L, penicillin (100×10^3^ U/L) and streptomycin (100 mg/L). The pH was maintained at 7.2–7.4 by equilibration with 5% CO_2_. The temperature was kept at 37°C. For subculturing, cells were rinsed with phosphate buffer solution (PBS) and incubated with Ethylenedinitrile tetraacetic acid (EDTA) and trypsin for cell detachment.

### 2.3 Cell viability assay

The MTT and MB assays determined the effect of BI and 5-FU on cell viability. Briefly, cells were seeded in a 96-well plate at 5.0×10^3^ for SGC-7901 cells and 6.0×10^3^ for MKN45 cells per well. After 24 h, different concentrations of BI and 5-FU were added. For MTT assays: After incubation for the desired period, 20 μl MTT (10 mg/ml in PBS stock, diluted to a working concentration of 2 mg/ml with media) was added to each well and incubated for 4h. After carefully removing the medium, 100 μl dimethyl sulfoxide (DMSO) was added to each well and shaken carefully, and the absorbance was measured at 492nm using a model microplate Reader. For MB assay: After incubation for the desired period, 50 μl of MB staining solution was added to each well, continued pregnancy for 1h, and then discarded the staining solution. After washed with distilled water, the cells were added 100 μl of Elution solution to each well, and shaken on a shaker at low speed for about 30 min until the crystals were completely dissolved. The absorbance was measured at 570nm. The experiments were conducted in triplicates.

### 2.4 Calculation of the combination index (CI) and evaluation of the joint effect

The EC_50_ values of SGC-7901 cells and MKN45 cells were determined using the cell viability from MTT assay for BI and 5-FU alone. The ratio of the EC_50_ values of BI and 5-FU was then used as a fixed concentration ratio to establish different dose combinations for the combination of the two. CompuSyn software analyzed data, and pharmacological interaction between BI and 5-FU was evaluated using the combination index (CI), where CI<1, CI = 1, and CI>1 indicate synergistic, additive, and antagonistic effects, respectively. Briefly, the CI was determined using the following formula: CI = (D)_1_/(DX)_1_+ (D)_2_/(DX)_2_, (DX)_1_ and (DX)_2_ are the concentrations required to achieve a certain level of inhibitory proliferation for BI and 5-FU alone, respectively, (D)_1_ and (D)_2_ are the respective concentrations required to achieve a certain level of inhibitory proliferation for BI and 5-FU in their combination (equivalent to that of a single drug).

### 2.5 Colony formation assay

To detect the ability of colony formation after different treatments, 400 cells in each well were seeded into 12-well plates. Following cell adhesion, the medium was replaced with medium-containing drugs; after 72 h, the medium was changed to RPMI-1640 and replaced every two days. After two weeks, the medium was removed, cells were washed twice with PBS, and then 4% paraformaldehyde was added. Finally, 0.1% crystal violet was utilized to stain the colonies, and ImageJ software was used to count the number of stained colonies.

### 2.6 Cell cycle assay

SGC-7901 cells and MKN45 cells were seeded at a density of 4.0 ×10^5^ per well in 6-well plates followed by overnight incubation to facilitate attachment and treated separately with BI and 5-FU alone, their combination for 72 h, and then collected and washed twice with cold PBS. After being fixed with 75% cold ethanol at −20°C for 24h, cells were stained by adding 500μl of the propidium iodide (PI) mixture in the dark (4°C, 30 min). Flow cytometry subsequently performed cell cycle analysis using a FACSCalibur analyzer (BD Biosciences). The DNA content at the phase of the cell cycle was analyzed using ModFit LT 5.0 software.

### 2.7 Mitochondrial membrane potential (MMP)

Cells were cultured in a 6 cm dish with the complete medium at 37°C and 5% CO_2_. When the cells reached 70%-90% confluence, BI or 5-FU or their combination working solution prepared with RPMI 1640 medium containing 2% fetal bovine serum was added, and the cells were cultured for 6 h, 12 h, and 24 h, respectively. The rhodamine 123 reagent was made into a working solution with distilled water to a final 5μg/ml. 0.75ml RPMI-1640 medium containing 2% FBS and 0.75ml rhodamine 123 working solution was added and incubated in the incubator for 30 min. After washing three times with PBS, the cells were collected by digestion and centrifugation, the supernatant was discarded, and 0.5ml PBS buffer was added. MMP in each group was measured by flow cytometry. A set of non-stained cell tubes was prepared for the blank control in each experiment.

### 2.8 Annexin V-FITC/PI for apoptosis detection

Cells were cultured in a 6 cm dish with the complete medium at 37°C and 5% CO_2_. When the cells reached 70%-90% confluence, BI or 5-FU or their combination working solution prepared with RPMI 1640 medium containing 2% fetal bovine serum was added, and the cells were cultured for 6 h, 12 h, and 24 h, respectively. The supernatants (floating apoptotic cells) were also collected, the adherent cells were trypsinized and then 195μl Annexin V-FITC conjugate, 5μl Annexin V-FITC staining solution, 10μl PI staining solution, gently resuspend the cells, and incubated the cells at room temperature (RT) (20–25°C) for 20 min away from the light. After centrifuging and discarding the supernatant, the cell pellets then were added 0.5 ml of Annexin V-FITC conjugate to each tube. After centrifugation, 0.5ml Annexin V-FITC was added to cell pellets and resuspended the cells for flow cytometry detection. The cells with PI-negative and Annexin V-negative were considered non-apoptotic cells. The cells with PI-negative and Annexin V-positive were considered apoptotic, and cells with both PI and Annexin V were considered necrotic.

### 2.9 Immunofluorescence staining

SGC-7901 cells were seeded in 6-well plates at a density of 5.0 ×10^4^ per well and incubated overnight. After treatment with BI and 5-FU alone or their combination for 24 h, cells were washed twice with PBS, and fixed using 4% paraformaldehyde overnight at 4°C. The cells were permeabilized with 0.1% Triton X-100 PBS solution (1.0 ml/well) for 10 min, then washed with PBS 3 times each for 5 min. The pGSK-3β primary antibody (1:100 dilution) was dropwise to add to the cells at 4°C overnight, after being washed with PBS 3 times each for 5 min, and then 250μl/well fluorescent secondary antibody (diluted 1:200) was added to incubate at room temperature (RT) for 90 min. After washed with PBS, the cells were added 50μl of DAPI-containing antifluorescent anti-quenching sealer and then covered with a coverslip. The cells were observed and images were taken under a fluorescence microscope.

### 2.10 Xenograft mouse model

The procedure and animal care were approved by the Ethics Committee of the Fourth Hospital of Harbin Medical University (2022-WZYSLLSC-12). Twenty female BALB/c nude mice (4 weeks old) were bought from Beijing Vital River Laboratory Animal Technology Co., Ltd (Beijing, China) and were kept and operated in a clean-grade animal room at the School of Life Sciences, Harbin Institute of Technology (Harbin, China). After one week of adaptation, SGC-7901 cells were injected subcutaneously in the right flanks of BALB-c nude female mice (0.2ml per mouse containing 2.0×10^6^ cells). Mice were randomly divided into four groups (5 mice/group): 1) the control group; 2) the BI group; 3) the 5-FU group; and 4) the BI + 5-FU group. BI (50 mg/kg) and 5-FU (30 mg/kg) were administered intraperitoneal injections every other day. The xenograft growth and mouse weight were monitored every 2 days, and xenograft volume was measured by caliper and calculated as (L × W^2^) / 2 (mm^3^) (L, length; W, width). After 4 weeks, all mice were euthanized using phenobarbital (50 mg/kg b.w.) by intraperitoneal (IP), and each xenograft was weighed and divided into two parts. The first part of the xenograft was formalin-fixed for immunohistochemical analysis, and the other part was snap-frozen in liquid nitrogen and stored at -80°C for Western blot analysis.

### 2.11 Protein extract and Western blot

After being treated with different concentrations of BI or 5-FU, SGC-7901 cells were harvested, washed twice times with PBS, and lysed at 4°C for 30 min in lysis buffer containing 150 mmol/L NaCl, 1.0 mL/L NP-40, 5.0 mg/L sodium deoxycholate, 100 g/L SDS, 50 mmol Tris (pH 7.4), 1 mmol/L DTT, 0.5mmol/L Na_3_VO_4_, 10 mmol/L phenylmethylsulfonyl fluoride (PMSF), 10 mg/L aprotinin, and 5 mg/L leupeptin as well as inhibitors of phosphorlatease (Sigma-Aldrich). After centrifuging at 10,000 g for 30 min at 4°C, the amount of total protein in the supernatant was determined using BCA assay. An equal amount of protein was loaded and separated on SDS-polyacrylamide gel electrophoresis, transferred to a nitrocellulose membrane (Gibco BRL, USA) overnight, blocked with 50 g/L defatted milk for 20 min, and the membranes then were hybridized with rabbit anti-pGSK-3β, mouse anti-Cyclin D1, anti-PCNA, and anti-CDK4 antibody. The membranes were incubated with anti-rabbit or anti-mouse horseradish peroxidase-conjugated IgG for 2 h at RT. Finally, the immunoreactive bands were detected using the enhanced chemiluminescence (ECL) system with X-ray film at the dark room. At the same time, β-actin was used as a housekeeping protein.

### 2.12 Hematoxylin-Eosin staining

Xenografts from nude mice were sectioned and deparaffinized in xylene, then dehydrated in alcohol gradients (100%, 95%, and 60%), and finally, distilled water. The sections were stained with hematoxylin for 2 min, rinsed with running water, stained with 1% eosin stain, placed in 100%, 95%, 60% ethanol, and xylene, and sealed with drops of neutral resin. A pathologist judged histologic changes.

### 2.13 Immunocytochemistry

Immunocytochemical staining was performed on serial sections at RT using the horseradish peroxidase method. The sections were deparaffinized in xylene and rehydrated through graded alcohol. The sections were incubated for 10 min at 95°C in 10 mmol/L sodium citrate (pH 6.0) buffer for PCNA staining. The tissue sections were immersed in sodium citrate repair solution, heated and boiled in a microwave oven for 30 min, and naturally cooled. Endogenous peroxidases were inactivated by engaging the sections in 3% hydrogen peroxide for 10 min and then were incubated for 10 min with 100ml/L normal goat serum in PBS to block non-specific binding. The sections were subsequently incubated overnight at 4°C with corresponding antibodies (1:50 dilution). The next day, the sections were incubated with biotinylated anti-mouse IgG for 2h, the chromogenic reaction was developed with DAB (diaminobenzidine) for 10 min, and all sections were counterstained with hematoxylin. Controls consisted of the omission of the primary antibody. The positive rate (PR) was calculated as follows: Number of positive cells PR (%) / Total number (2.0×10^4^) ×100.

### 2.14 TUNEL assay

The TUNEL assay was performed as described in a previous study [19]. Briefly, after re dehydrated, the sections were treated with proteinase K (20 μg/ml) for 30 min at RT, then added TdT reaction at 37°C for 1 h, and finally added incubated anti-digoxigenin conjugate for 30 min at RT. The sections were visually colored using DAB, counterstained with hematoxylin, and counted the positive number of cells per field under a microscope (40×).

### 2.15 Statistical analysis

All statistical analyses were analyzed using SPSS23 software (IBM Inc.) and plotted using GraphPad Prism 7.0 (San Diego, CA). The quantitative data are presented as the mean ± standard deviation (S.D.). All experiments and analyses were performed in triplicate. The Kruskal-Wallis H test was used to compare the differences between different experimental groups; differences between means were analyzed for significance using the one-way ANOVA test with Turkey’s multiple comparisons, which was used to assess the difference between independent groups. Differences were considered significant at *P*<0.05.

## 3. Results

### 3.1 Effects of BI and 5-FU alone or their combination on proliferation and colony formation of gastric cancer cells

**Fig 1:**
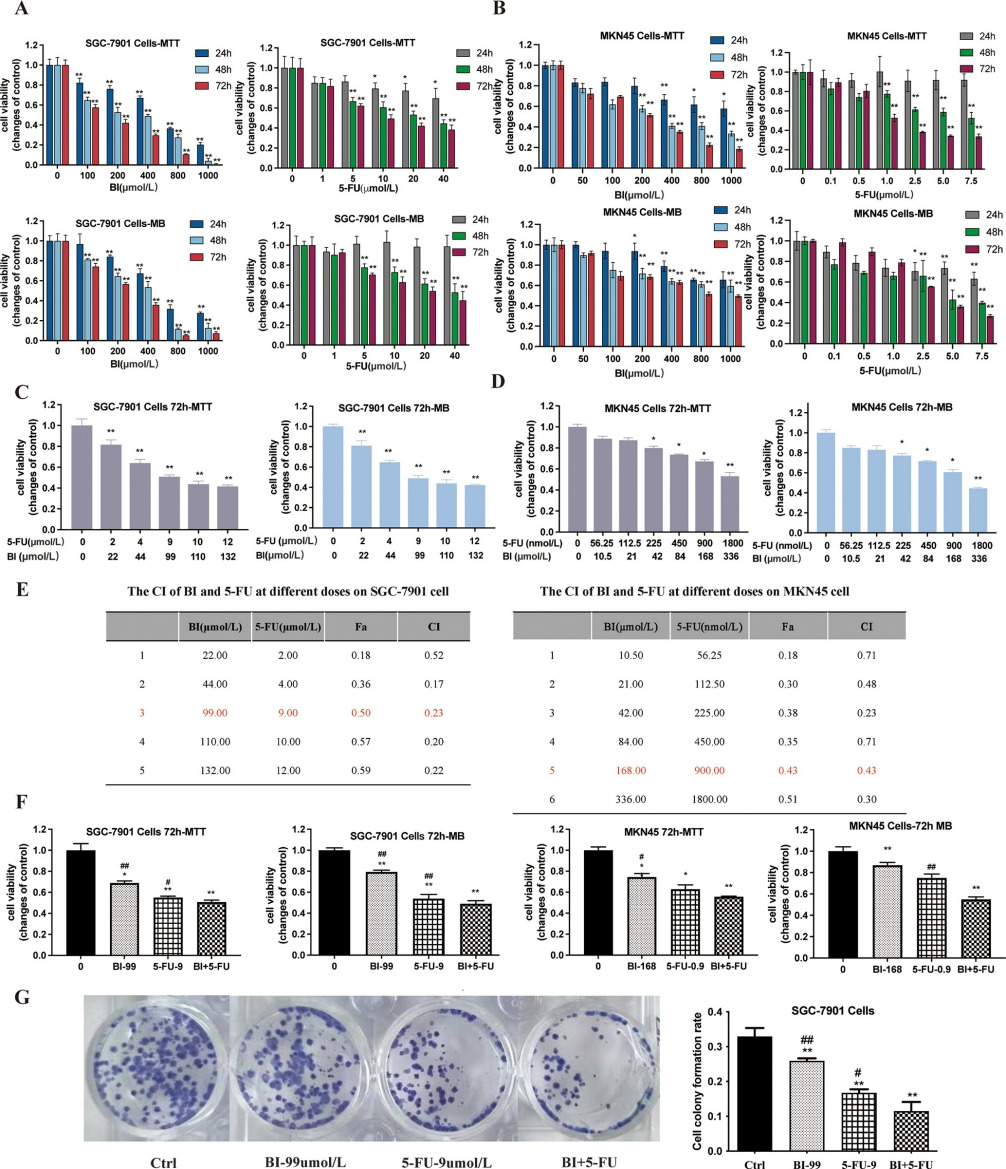
β-Ionone (BI) enhanced 5-fluorouracil (5-FU) to inhibit proliferation of SGC-7901 cells and MKN45 cells. (A) SGC-7901 cells were treated with different doses of BI (0, 100, 200, 400, 800, and 1000 μmol/L) and 5-FU (0, 1, 5, 10, 20, and 40 μmol/L) for 24, 48, and 72 h. The cell viability was assayed by MTT and MB assays; (B) MKN45 cells were treated with different doses of BI (0, 50,100, 200, 400, 800, and 1000 μmol/L) and 5-FU (0, 0.1, 0.5, 1.0, 2.5, 5.0, and 1.5 μmol/L) for 24 h, 48 h, and 72 h. The cell viability was assayed by MTT and MB assays; (C) The viability of SGC-7901 cells treated with BI and 5-FU at a ratio of 11:1 was detected by MTT and MB assays; (D) The viability of MKN45 cells treated with BI and 5-FU at a ratio of 187:1 was detected by MTT and MB assays; (E) The CI values of the different effects on 5-FU combined with BI in SGC-7901 cells and MKN45 cells; (F) Effects of 5-FU (9 μmol/L) in combination with BI (99 μmol/L) on cell viability of SGC-7901 cells, and 5-FU (0.9 μmol/L) in combination with BI (168 μmol/L) on cell viability of MKN45 cell viability; (G) Colony formation of SGC-7901 cells treated with BI (99μmol/L) and 5-FU (9μmol/L) alone or their combination for 14 days. The data are expressed as the mean of 3 independent experiments with 3 replicates per experiment. Compared with the control group, *P<0.05, **P<0.01; Compared with the combination of BI with 5-FU, ^#^*P*<0.05, ^##^*P*<0.01.

Whether BI and 5-FU inhibited proliferation of human gastric cancer cells was first examined by MTT and MB assays, SGC-7901 cells and MKN45 cells were treated with BI at the concentrations of 0 ~ 1000 μmol/L, 5-FU at the concentrations of 0 ~ 40 μmol/L, respectively. Both BI and 5-FU inhibited cell proliferation in two cell lines in a dose- and time-dependent manner (Fig 1A and 1B). The EC_50_ values of BI and 5-FU in SGC-7901 cells for 72 h were (186±27.84) μmol/L and (17±3.75) μmol/L, respectively, while the EC_50_ values in MKN-45 cells were (336+34.92) μmol/L and (1.8+0.17) μmol/L, respectively. The EC_50_ ratio of BI and 5-FU acting alone was chosen in SGC-7901 cells and MKN45 cells for 72 h treatment as the fixed-dose ratio of the combination, 11:1 and 187:1, respectively. Based on these results, the viability was evaluated in SGC-7901 cells and MKN45 cells treated with various concentrations of BI and 5-FU in their combination for 72 h. The combination of BI and 5-FU showed concentration-dependent anti-proliferation effects on gastric cancer cells (Fig 1C and 1D).

To assess whether the combination of BI and 5-FU showed synergistic enhancement of cytotoxicity, the combination index (CI) values were calculated using CompuSyn software based on the median effect principle (Chou, 2006). The obtained CI values showed positive interactions between BI and 5-FU and found cooperative interaction for all BI and 5-FU combination doses in SGC7901 cells and MKN45 (Fig 1E). Finally, the concentrations of BI (99μmol/L) and 5-FU (9μmol/L) were selected for SGC-7901 cells, BI (168μmol/L) and 5-FU (0.9 μmol/L) for MKN45 cells for follow-up experiments, which CI values were 0.23 and 0.43, respectively. The results showed that BI significantly enhanced inhibitory effects of 5-FU on the viability of SGC-7901 cells and MKN45 cells (Fig 1F). Further, compared to either BI or 5-FU alone, the combination of BI and 5-FU induced a significant decrease in colony formation (Fig 1G). These results indicated that BI could synergistically enhance the inhibitory effect of 5-FU on the proliferation and colony formation of gastric cancer cells.

### 3.2 Effects of BI and 5-FU alone or their combination on the cell cycle distribution in gastric adenocarcinoma cells

**Fig 2:**
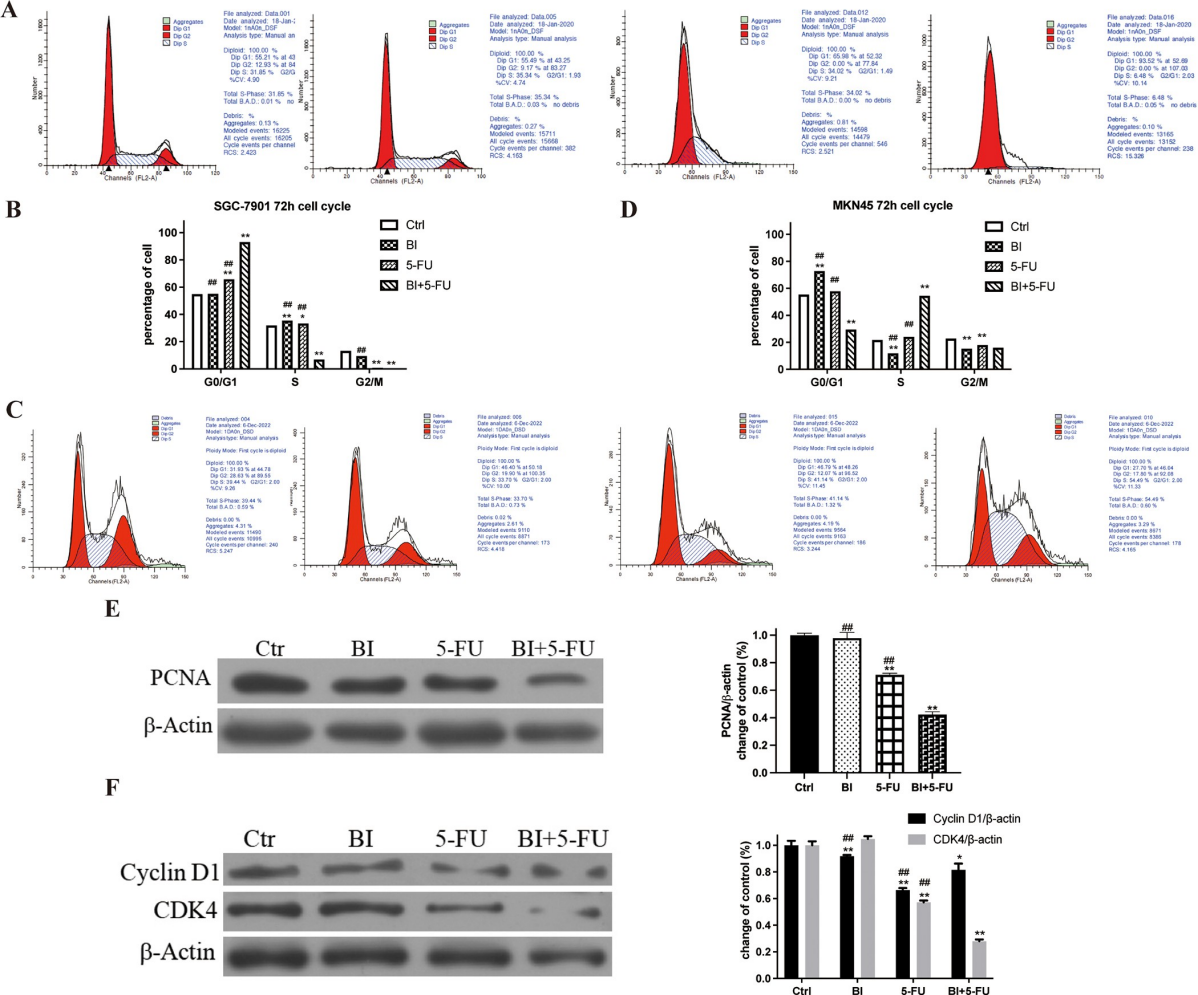
Effects of BI, 5-FU alone and their combination on the cell cycle of SGC-7901 cells and MKN45 cells. (A, B) SGC-7901 cells were harvested after treatment with BI (99 μmol/L) and 5-FU (9 μmol/L) alone or their combination for 72 h. The cell cycle of SGC-7901 cells was detected by flow cytometry with PI staining; (C, D) MKN45 cells were harvested after treatment with BI (168 μmol/L) and 5-FU (0.9 μmol/L) alone or their combination for 72h. Flow cytometry detected MKN45 cells’ cell cycle after PI staining;(E, F) The Western blot was used to assess PCNA, Cyclin D1, and CDK4 expression after SGC-7901 cells treated with BI and 5-FU alone or their combination for 72h. Compared with the control group, *P<0.05, **P<0.01; Compared with the combination of BI with 5-FU, ^#^*P*<0.05, ^##^*P*<0.01.

Flow cytometry was used to evaluate the changes in the distribution of cell cycle to clarify the reason why the mechanisms of BI+5-FU were the inhibitory proliferation of gastric adenocarcinoma cells. Compared with the control group, the number of G0/G1 phase cells in SGC-7901 cells treated with BI and 5-FU alone or in their combination increased from (54.90±0.01) % to (55.15±0.01) %, (65.84±0.02) % and (93.13±0.01) %, respectively. The cell cycle was arrested at the G0/G1 phase in SGC-7901 cells (Fig 2A and 2B). For MKN45 cells, BI alone increased the cell number of G0/G1 phase, the proportion of S-phase cells was increased by 5-FU alone, and the proportion of S-phase cells was further increased to (54.57±2.59) % by their combination compared to the 5-FU group. The cell cycle was arrested at the S phase in gastric cancer cells (Fig 2C and 2D).

In addition, the expression of proliferating cell nuclear antigen protein (PCNA) was examined in SGC7901 cells by Western blot. 5-FU significantly downregulated the expression of PCNA compared with the control group, and the combination of BI and 5-FU also decreased the expression of PCNA protein more significantly (*P*<0.01) (Fig 2E). To investigate the mechanism of action further, SGC-7901 cells were selected to examine the effects of BI and 5-FU on the cell cycle regulatory protein—Cyclin D1 and CDK4. The results showed that 5-FU inhibited the expression of Cyclin D1 and CDK4, and the inhibitory effect on CDK4 was more significant when combined with BI (Fig 2F). These results suggested that BI and 5-FU inhibited the proliferation of SGC-7901 cells by arresting the cell cycle at the G0/G1 phase and changed the protein expression of cell cycle-related proteins—CDK4 and Cyclin D1.

### 3.3 Effects of BI and 5-FU alone or their combination on apoptosis in SGC-7901 cells

**Fig 3:**
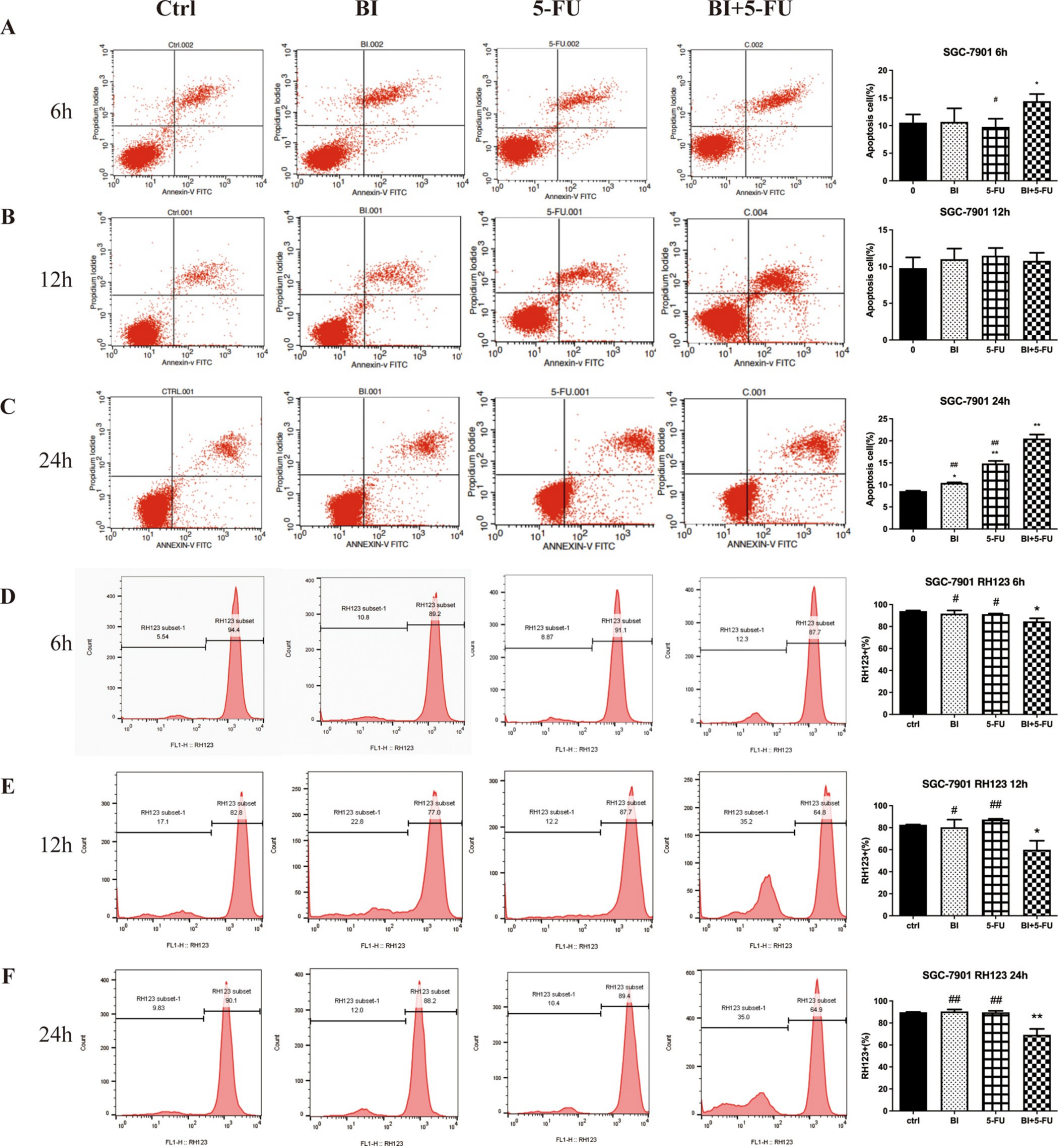
Combination of BI and 5-FU induced apoptosis of SGC7901 cells. Apoptosis of SGC-7901 cells was detected by Annexin V-FITC/PI double staining assay after 6 h (A), 12 h (B), and 24 h (C) of treatment with BI, 5-FU alone or their combination. The changes of mitochondrial membrane potential (MMP) in SGC-7901 cells were detected by RH-123 staining after treatment with BI, 5-FU alone or their combination for 6 h (D), 12 h (E) and 24 h (F). The data shown are representative of one of three independent experiments. Compared with the control group, *P<0.05, **P<0.01; Compared with the combination of BI with 5-FU, ^#^*P*<0.05, ^##^*P*<0.01.

Based on the above findings, the effects of BI and 5-FU alone and their combination on apoptosis were further investigated in gastric cancer cells. Annexin V-FITC/PI assay was used to detect the apoptosis of SGC-7901 cells. After 6, 12, and 24 h of treatment with the individual and 5-FU combined BI, there was no significant change in the number of apoptotic cells among the groups after 12 h (Fig 3B). Still, after 6 h of treatment, the number of apoptotic cells in the combined treatment group was higher than that in the control group (Fig 3A). For 24 h of treatment, compared with the control group, the number of apoptotic cells increased from 8.47% to 10.31%, 14.19%, and 21.24% in the BI, 5-FU, and BI + 5-FU groups, respectively, the number of apoptotic cells increased significantly in the combination of BI and 5-FU (Fig 3C).

The changes in mitochondrial membrane potential, considered a marker of early apoptosis, were assessed by RH-123 staining. At 6 h, the number of cells bound to RH-123 dye changed from 94.4% to 89.2%, 91.1%, and 87.7% in the BI, 5-FU, and BI+5-FU groups, respectively (Fig 3D). After 12 h of treatment, it changed from 82.8% to 77.0%, 87.7%, and 64.8% (Fig 3E). It changed from 90.1% to 88.2%,89.4%, and 64.9% after 24h of treatment (Fig 3F). The results of RH123 staining showed that BI and 5-FU alone had little effect on mitochondrial membrane potential (MMP), but 5-FU combined with BI had a time-dependent destruction of MMP. These results suggested that BI and 5-FU had a synergistic effect on inducing apoptosis of gastric cancer cells through the mitochondrial pathway.

### 3.4 Effects of BI and 5-FU alone and their combination on the growth of xenografts in a nude mice model

**Fig 4:**
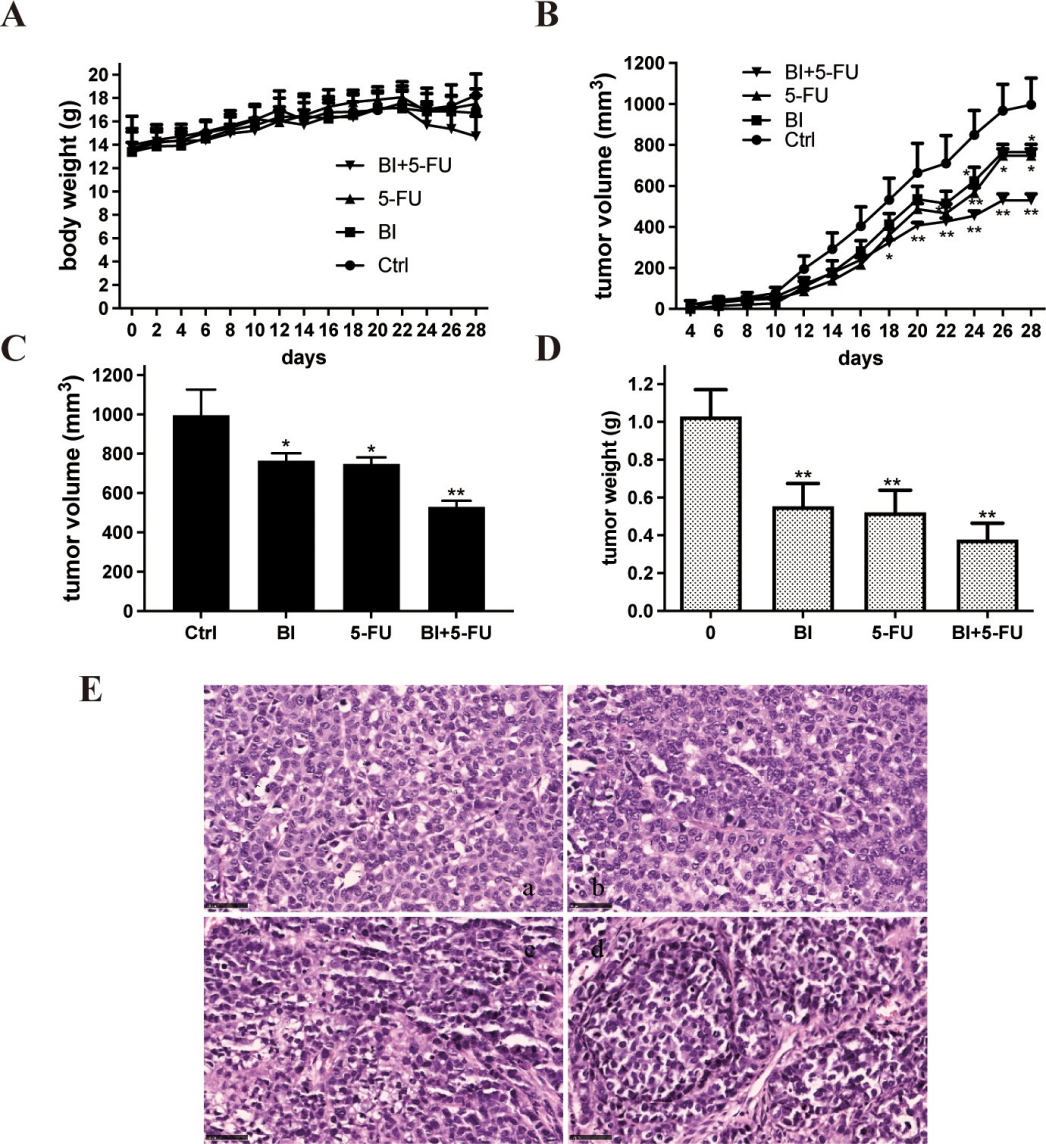
BI enhanced the ability of 5-FU to inhibit the growth of SGC-7901 cell xenograft in nude mice. After inoculation of SGC-7901 cells, nude mice were injected with BI (50mg/kg) and 5-FU (30mg/kg) alone or their combination for 4 weeks. (A) The body weight changes of mice during the treatment; (B) The tumor volume changes; (C) The tumor volume at the end of treatment; (D) The tumor weight at the end of treatment; (E) HE staining of SGC-7901 cell xenografts. Magnification = ×40. Scale bar = 40 μm. n = 5 mice per group. Compared with the control group, *P<0.05, **P<0.01.

Our findings suggest that BI enhances the inhibitory effects of 5-FU on the proliferation of gastric adenocarcinoma cells. To further confirm the synergistic effect of 5-FU combined with BI in vivo, a subcutaneous xenograft model in nude mice was established by the implantation of SGC-7901 cells. After implantation for 24h, the mice were injected intraperitoneally with 20% DMSO as a negative control, BI (50mg/kg) alone, 5-FU (30mg/kg) alone, or their combination. The volume of SGC-7901 cell xenografts and body weight of mouse changes were measured during the treatment administration. The results revealed that the given doses of 5-FU or BI did not produce toxic effects on mice compared to the control group (Fig 4A). The mice treated with DMSO, BI, and 5-FU showed a trend of progressive xenograft enlargement.

In contrast, the xenograft volume in mice treated with their combination was significantly smaller than in the other three treatment cohorts (Fig 4B). The final volume and weight of xenograft were the smallest in the BI combined with the 5-FU group (Fig 4C and 4D). To observe the morphological changes of xenograft cells in nude mice after the effects of BI, 5-FU, and BI+5-FU, xenograft sections were performed by HE staining. The results showed that the morphological effects of BI and 5-FU on xenografts were not significantly different from those of the control group (Fig 4E).

### 3.5 Effects of BI and 5-FU alone or their combination on proliferation and apoptosis of xenografts in nude mice

**Fig 5:**
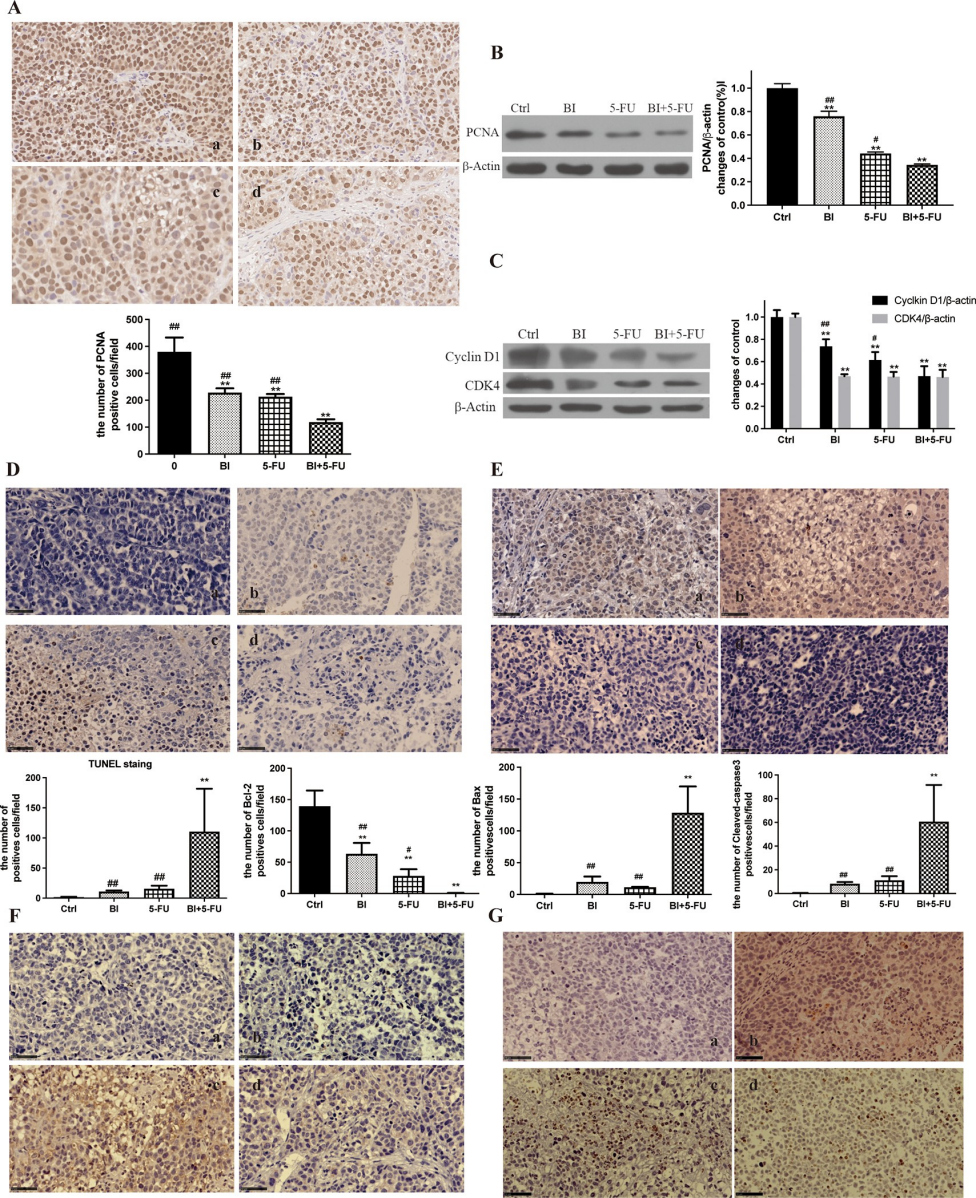
Effects of BI and 5-FU alone or their combination on the proliferation and apoptosis of SGC-7901 cell xenografts in nude mice. (A) The expression of PCNA was detected in xenografts by IHC; (B) The expression of PCNA was detected in xenografts by Western blot; (C) The expression of Cyclin D1 and CDK4 in xenografts; (D) The number of apoptotic cells was detected in xenografts by TUNEL; (E, F, G) The expression of Bcl-2, Bax, and Cleaved caspase-3 proteins were detected in xenografts by IHC. a, b, c and d stand for the control, BI, 5-FU and BI_5-FU groups. Magnification = ×40. Scale bar = 50 μm. Compared with the control group, **P<0.01; Compared with the combination of BI with 5-FU, ^#^*P*<0.05, ^##^*P*<0.01.

To better understand the potential molecular mechanisms of the combination of 5-FU and BI, the expression of proteins related to the cell cycle and apoptosis was examined in SGC-7901 cell xenografts. The level of PCNA in xenografts was significantly lower in the BI + 5-FU group than in the other groups (Fig 5A). The same trend was also found in the results of Western blot (Fig 5B). Meanwhile, the expression of cyclin D1 and CDK4 was also significantly reduced in the BI + 5-FU group (Fig 5C), suggesting that BI enhanced the inhibitory effect of 5-FU on growth of xenografts in vivo.

The TUNEL assay was used to detect apoptosis cells in the BI, 5-FU, and BI+5-FU groups. Compared with the control group, there was no significant difference in apoptosis cells between the BI and 5-FU groups, but the number of apoptotic cells was considerably higher in the BI+5-FU group than that in other groups (Fig 5D). There were also significant changes in the expression of Bcl-2 (Fig 5E) and Bax (Fig 5F) proteins in the BI+5-FU group. The expression of cleaved-caspase-3 protein was not substantially altered by BI or 5-FU alone but was dramatically elevated by BI + 5-FU compared to the control group (Fig 5G). These results indicate that 5-FU combined with BI increased apoptosis of SGC-7901 cell xenografts.

### 3.6 GSK-3β was a target for BI regulating the anticancer effects of 5-FU on gastric cancer

**Fig 6:**
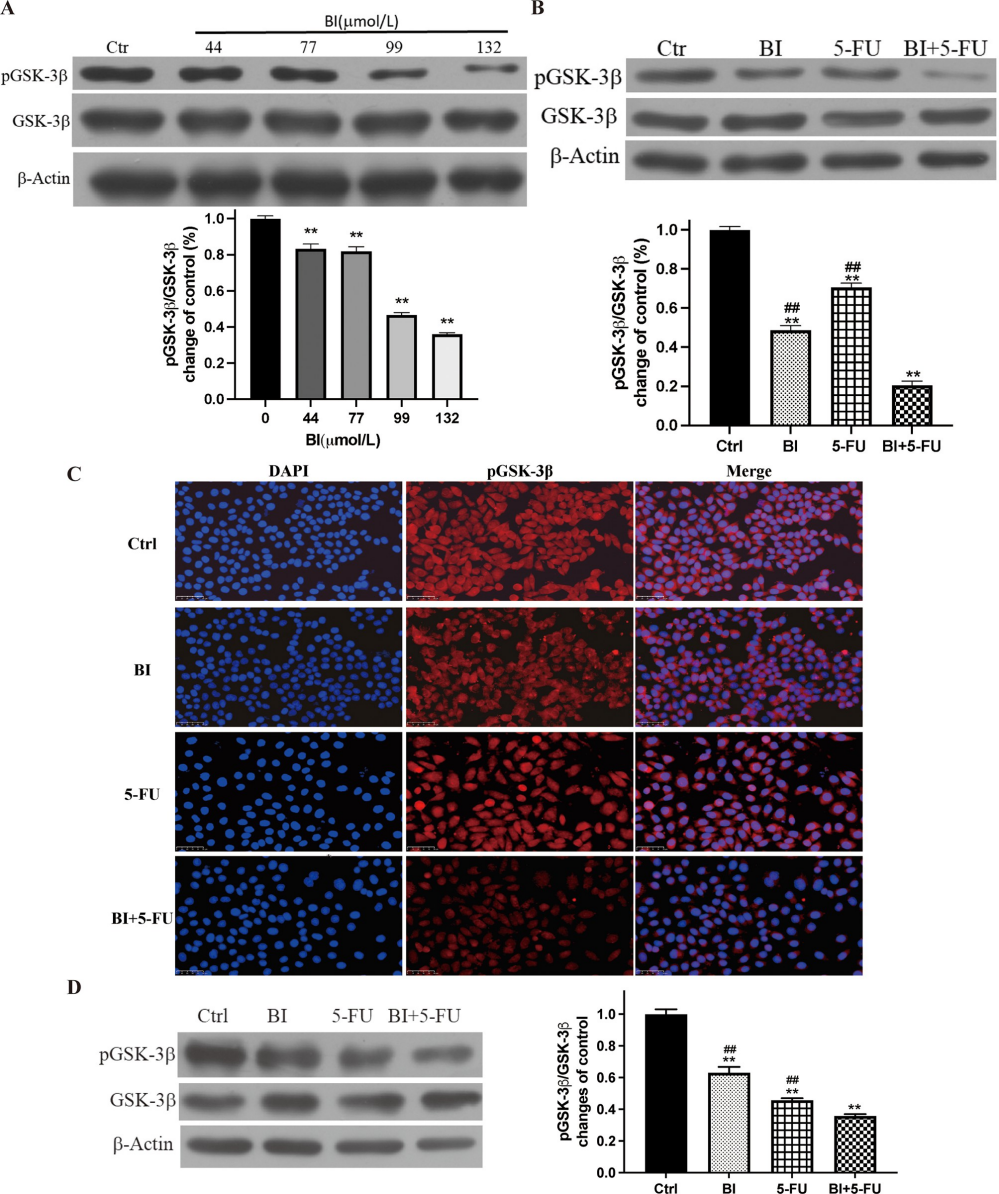
GSK-3β was a target for BI regulating the antitumor effects of 5-FU on gastric cancer cells. (A) The expression of pGSK-3β protein was examined in SGC-7901 cells treated with different doses of BI by Western blot; (B) The expression of pGSK-3β protein was examined in SGC-7901 cells treated with BI and 5-FU alone and their combination; (C) The changes of pGSK-3β protein expression in SGC-7901 cells by immunofluorescence assay; (D) The expression of pGSK-3β protein in SGC-7901 cell xenografts. Compared with the control group, **P<0.01; Compared with the combination of BI with 5-FU, ^##^*P*<0.01.

Our earlier research demonstrated that BI can regulate the PI3K/AKT signaling pathway [21]. AKT adversely controls the activity of GSK-3β since it is a significant substrate of AKT. GSK-3β activity will better understand the potential molecular processes of 5-FU combined with BI treatment. The significant dose-response relationship between BI and pGSK-3β (ser9) protein expression indicated that BI enhanced GSK-3β protein activity (the active unit of GSK-3β protein is non-phosphorylation) (Fig 6A). Additionally, when 5-FU was combined with BI, the expression of pGSK-3β protein decreased with time and was 80% lower after 72 h of treatment compared to the control group (Fig 6B). Immunofluorescence was also employed to determine how medication treatment affected the distribution and expression of the pGSK-3β protein. After 24 h of treatment with BI, 5-FU alone, or their combination, the nucleus’ position could be located by DAPI, and pGSK-3β was red and localized in the cytoplasm, in contrast to the control group. BI and 5-FU alone or their combination caused the expression of pGSK-3β to display varying degrees of red fluorescence attenuation (Fig 6C). Subsequent analysis of pGSK-3β protein expression in xenografts showed that pGSK-3β expression of the BI and 5-FU alone groups had different degrees of decrease, the degree of down-regulation was more pronounced in the combination group (Fig 6D).

## 4. Discussion

5-Fluorouracil is widely used as a first-line anticancer drug to treat gastric cancer [23]. However, the rapid emergence of drug resistance and systemic toxicity has limited its clinical practice. Cancer therapies combining plant-derived active ingredients and chemotherapeutic agents such as β-ionone are receiving increasing attention as a novel method to increase efficiency and reduce toxicity [24, 25]. It has been reported in the literature that baicalein (WOG) in combination with 5-FU inhibited the proliferation of MGC-803 cells in a concentration-dependent manner. 5-FU can be used at relatively low concentrations to exhibit the effects of 5-FU on synergistic anticancer [26]. BI is a cyclized isoprenoid and a terminal ring analog of β-carotene, a natural flavor found mainly in fruits and grains [27, 28]. Numerous studies have confirmed its anti-inflammatory, anticancer, antibacterial, and antifungal effects [14, 29–31]. In the present study, our findings demonstrated for the first time that BI could enhance 5-FU-mediated antitumor effects on gastric cancer cells using cell viability (Fig 1), cell proliferation (Fig 4), cell cycle distribution (Fig 2), and cell apoptosis assays (Fig 3).

Several studies have focused on the anti-tumor properties of BI in different tumor models. BI can inhibit the proliferation and migration of melanoma cells by activating OR51E2 [17] and can inhibit the proliferation of prostate cancer cells by down-regulating cell cyclin-related proteins [32]. β-Ionone also inhibits renal-cell carcinoma progression by activating LKB1/AMPK-triggered autophagy [18]. Additionally, our data have demonstrated that BI could inhibit the activity of COX-2 [14] to suppress proliferation of breast cancer cell, through the MARK pathway in gastric cancer cells to arrest the cell cycle [12], and inhibit the PI3K - AKT pathway to trigger apoptosis of gastric cancer cells [22].

In this study, BI, when combined with 5-FU, had a synergistic impact on the suppression of proliferation of SGC-7901 cells and MKN45 cells in a dose-dependent manner (Fig 1A and 1B). The Combination Index (CI) of 5-FU and BI was calculated and analyzed by CompuSyn software. The Chou-Talalay method was used to determine the interaction between the two drugs based on the magnitude of the CI value, in which the CI < 1, = 1, and > 1 indicated synergistic, additive, and antagonistic effects, respectively. The combination of 5-FU (9 μmol/L) and BI (99 μmol/L) was shown to inhibit proliferation of SGC-7901 cells and MKN45 cells in a dose-dependent manner. It indicates a synergistic effect on the viability of gastric cancer cells treated with 5-FU in combination with BI. The CI values of 5-FU (9μmol/L) combined with BI (99μmol/L) in SGC-7901 cells was 0.23, which showed a strong synergistic effect, while the CI value of BI (168μmol/L) combined with 5-FU (0.9μmol/L) in MKN45 cells was 0.43, which was in the range of 0.3~0.7, and showed a moderate synergistic effect (Fig 1E). The combination of BI and 5-FU significantly reduced the cell viability of gastric adenocarcinoma cells compared to the control and 5-FU alone, and the reduced dose of the combination achieved the same effect as that produced by 5-FU alone (Fig 1F). The colony formation assay is a more sensitive indicator to assess the long-term effects of a drug than cell activity assay, which reflects the potential clone-forming potential of potential tumor stem cells. Our results also showed fewer and smaller colonies in the combination group compared to the control and 5-FU or BI alone (Fig 1G). To further verify that BI can enhance inhibitory proliferation effects of 5-FU on gastric adenocarcinoma cells, a nude mouse model of SGC-7901 cells was established to evaluate the growth of xenograft and the effect of combination treatment, and to confirm the sensitizing effect of BI on 5-FU at a deeper and more comprehensive level. The xenografts in the combination group were significantly smaller than in the control and 5-FU alone groups, the nude mice in each group did not show any significant signs of toxicity (Fig 4). Therefore, by lowering the dosage of chemotherapy medicines while also having an adjuvant therapeutic impact on xenografts, the combined use of phytochemicals and chemotherapeutic agents may be a means to lessen the toxicity of chemotherapeutic agents.

Cell proliferation is closely related to the cell cycle [33]. It has been well-documented that natural phytochemicals can cause the cell cycle arrest in tumors. Honokiol induces the cell cycle arrest at the G0/G1 phase in oral squamous carcinoma OC2 cells and OCSL cells in a time-dose-dependent manner [34]. BI also induces cell cycle arrest at the G1 phase by down-regulating CDK4 and cyclin D1 in DU145 cells and PC-3 cells [32]. Treatment of colon cancer cells with sub-toxic BI resulted in dose-dependent proliferative inhibition, the G1-S phase arrested, and induction of apoptosis [35]. Our group previously found that BI could cause the cell cycle arrested at the G1/G0 phase in breast cancer cells.

In contrast, BI at different doses inhibited cell proliferation and DNA synthesis of SGC-7901 cells and arrested the cell cycle at the G0/G1 phase. This degree of inhibition was a dose-dependent manner [12]. In this study, consistent with previous research, BI could not only arrest the cell cycle at the G1 phase in SGC-7901 cells, but also increase the number of cells at the G1 phase when BI combined with 5-FU (Fig 2A and 2B). In MKN45 cells, 5-FU could arrest the cell cycle at the S phase, and the number of cells in the S phase increased after 5-FU combined with BI (Fig 2C and 2D). After treatment for 72h, 5-FU resulted in the down-regulation of the expression of CDK4 and cyclin D1 proteins in SGC-7901 cells, but it was more pronounced when it was combined with BI (Fig 2F). These results were also confirmed in vivo (Fig 5C). The proliferating cell nuclear antigen (PCNA) is a cell cycle protein with a role in DNA damage, which interacts with the S-phase specific complex (CDK2-Cyclin A), suggesting that PCNA has a functional role in binding to cyclin-dependent protein kinase complex [36]. Our results showed that BI and 5-FU alone reduced the expression of PCNA in SGC-7901 cells, and this decrease was more significant after the combination of 5-FU and BI (Figs 2E and 5B). The number of PCNA-positive cells in the combined treatment group was significantly lower than that in the control or the single treatment groups (Fig 5A). These results suggest that BI can synergize with 5-FU to significantly inhibit the proliferation of human gastric adenocarcinoma cells.

Apoptosis, also known as programmed cell death, is regulated through a series of signaling mechanisms to maintain the balance and homeostasis of cell growth and development [11]. Many studies have been conducted on the pharmacological effects and physiological functions of apoptosis; 5-FU, as a chemotherapeutic agent, can be used in digestive tract cancer cells to induce apoptosis of tumor cells and exert anticancer activities. BI can induce apoptosis in various cells in breast, colon, and gastric cancer cells by affecting the relevant signaling pathways [11, 37–39]. On this basis, the Annexin V-FITC/PI assay was used to detect apoptosis, and the results of 5-FU combined with BI for 24 h showed that the number of apoptotic cells in the BI+5-FU group was significantly increased compared with the control, BI, and 5-FU alone (Fig 3A–3C). RH123 dye can be transmitted through the cell membrane and bind to the mitochondrial matrix; when the MMP decreases, it suggests that early apoptosis occurs, and it is considered an important marker for the early stage of apoptosis [39]. Our findings showed that BI and 5-FU could damage the mitochondrial membrane potential (MMP), but the combination of BI and 5-FU had a more noticeable decrease in MMP (Fig 3D–3F). The TUNEL assay also showed a significant increase in apoptotic cells when this combination was applied (Fig 5D). Immunohistochemistry (IHC) was used to detect apoptosis-related proteins—Bcl-2, Bax, and Cleaved-caspase-3 in SGC-7901 cell xenografts (Fig 5E–5G). Our findings suggest that BI can synergize with 5-FU to increase apoptotic induction in gastric cancer cells through the mitochondrial pathway. Therefore, natural phytochemicals are used to inhibit cell proliferation or induce apoptosis by blocking the cell cycle of gastric cancer cells, arising from a great interest in a therapeutic option for tumor treatment.

The possible mechanism by which BI synergistically enhances the anti-tumor efficacy mediated by 5-FU was further investigated in a pathway of glycogen synthase kinase-3β (GSK-3β), which may be a target of BI to regulate the anti-tumor effects of 5-FU. The PI3K-AKT pathway is involved in cell proliferation, survival, and cell signaling, such as prostate cancer and breast cancer, while mitochondria is an important intersection point of the PI3K/AKT signaling pathway [40–42]. BI can regulate the PI3K-AKT signaling pathway to induce apoptosis by inhibiting the expression of pPI3K and pAKT in a dose-dependent manner [21]. Many studies have confirmed that the regulation of PI3K/AKT/GSK-3β plays a vital role in differentiating gastric adenocarcinoma cells [43]. GSK-3β is a multifunctional cytoplasmic protein kinase, which acts as a downstream substrate in the PI3K/AKT signaling pathway and participates in a variety of signaling pathways such as Wnt/β-catenin, Hedgehog [44]. The activity of GSK-3β is regulated by site-specific phosphorylation, and the phosphorylation of Ser-9 inhibits the activity of GSK-3β; that is, pGSK-3β (Ser9) is its inactive form [45]. Although GSK-3β and Cyclin D1 are known to be involved in cell cycle regulation, their relationship in cancer cells remains controversial. It has been shown that activated GSK-3β inhibits Cyclin D1 expression in tumor cells, including breast cancer [46] and tongue squamous carcinoma [47]. In contrast, GSK-3β did not correlate with Cyclin D1 expression in hepatocellular carcinoma cells [48] and fibroblasts [49]. However, in our study, BI could synergize with 5-FU to enhance GSK-3β protein activity by participating and inhibiting the expression of pGSK-3β (Ser-9) (Fig 6). On the other hand, it increases the Bax/Bcl-2 protein ratio and the expression of Cleaved-caspase-3 protein, which initiates the caspase3 cascade reaction through the mitochondrial pathway, thus inducing apoptosis in human gastric adenocarcinoma cells.

## 5. Conclusion

Our results presented in this study suggest that the combination of 5-FU and BI processes a synergistic effect on proliferative inhibition and apoptosis induction by enhancing the GSK-3β pathway against gastric adenocarcinoma cells. These findings provide a new strategy for developing phytochemicals such as BI as adjuvants to chemotherapeutic drugs, thereby reducing the toxicity and resistance induced by chemotherapeutic drugs.

## Supporting information

S1 Data (PDF)

S2 Data (PDF)

S3 Data (PDF)

## Abbreviations

5-FU: 5-Fuorouracil
Bax: Bcl2-associated X
Bcl-2: B-cell lymphoma-2
BI: 4-(2,6,6-trimethyl-1-cyclohexen-1-yl)-3-buten-2-one, (β-ionone)
CDK4: Cyclin-dependent kinase
CI: Combination index
DAPI: 2-(4-Amidinophenyl)-6-indolecarbamidine dihydrochloride
DMSO: Dimethyl sulfoxide
EC50: Median effect concentration
FBS: Fetal bovine serum
GSK-3β: Glycogen synthase kinase-3beta
IHC: Immunohistochemistry
MB: Methylene blue
MMP: Mitochondrial membrane potential
MTT: 3-(4,5-Dimethylthiazol-2-yl)-2,5-diphenyltetrazolium bromide
PBS: Phosphate saline buffer
PCNA: Proliferating cell nuclear antigen
PI: Propidium iodide
PMSF: Phenylmethylsulfonyl fluoride
SDS: Sodium dodecyl sulfate-Na salt
