# Supplementary material for: β-Ionone enhances the inhibitory effects of 5-fluorouracil on the proliferation of gastric adenocarcinoma cells by the GSK-3β signaling pathway
Source: PLoS One. 2024 Sep 6;19(9):e0309014. doi: 10.1371/journal.pone.0309014 (PMC11379261; doi:10.1371/journal.pone.0309014)
Supplement: S1 Data — (PDF) [file pone.0309014.s001.pdf]

Figure 1G Colony formation of SGC-7901 cells treated with BI (99μmol/L) and 5-FU (9μmol/L) alone or a combination for 14 days.

SGC-7901 colony formation

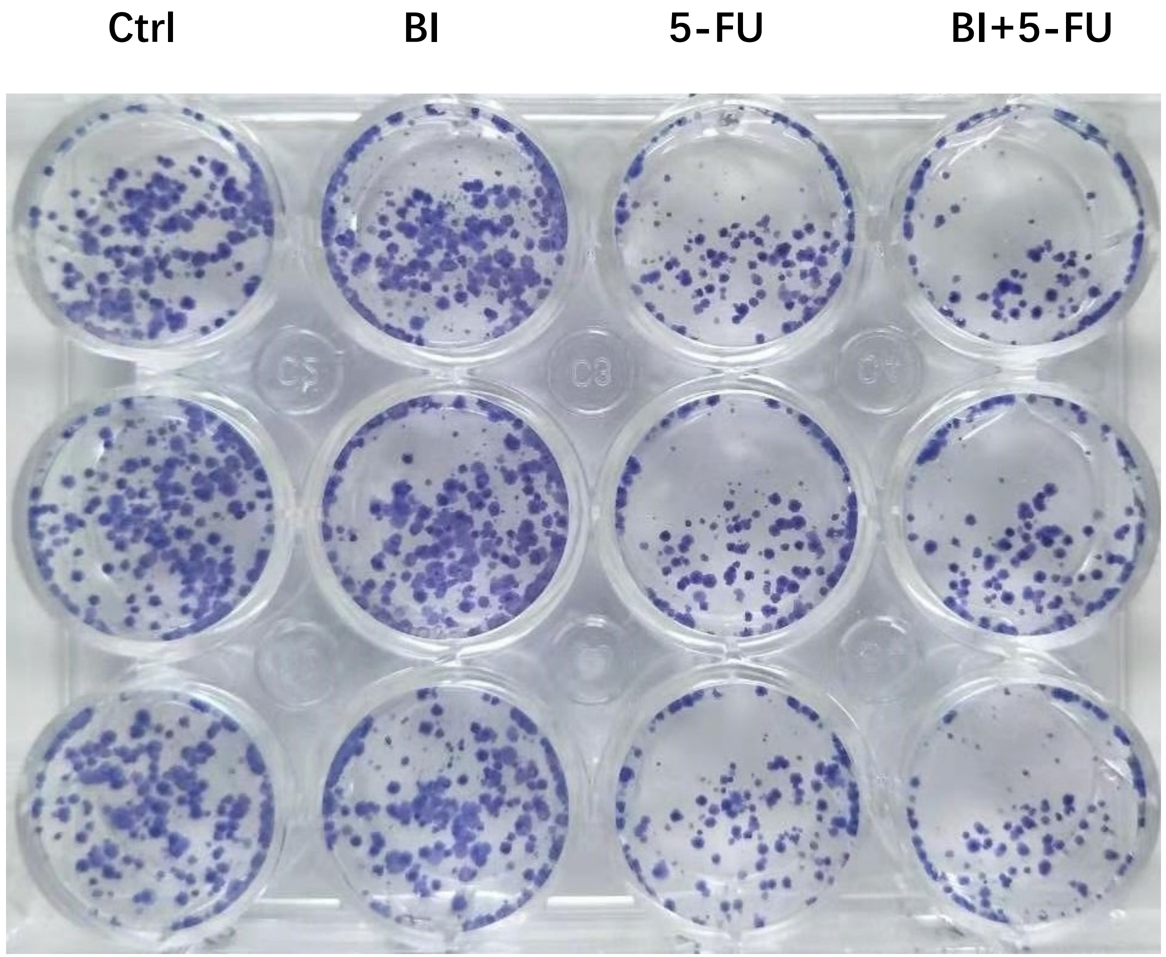

0	BI	5-FU	BI+5-FU
134	102	71	41
140	107	63	58
121	102	67	39

Ctrl	BI-99	5-FU-9	BI+5-FU
0.3350	0.2550	0.1775	0.1025
0.3500	0.2675	0.1575	0.1450
0.3025	0.2550	0.1675	0.0975

Figure 2A SGC-7901 cells were harvested after treatment with BI (99 μmol/L) and 5-FU (9 μmol/L) alone or in combination for 72 h.

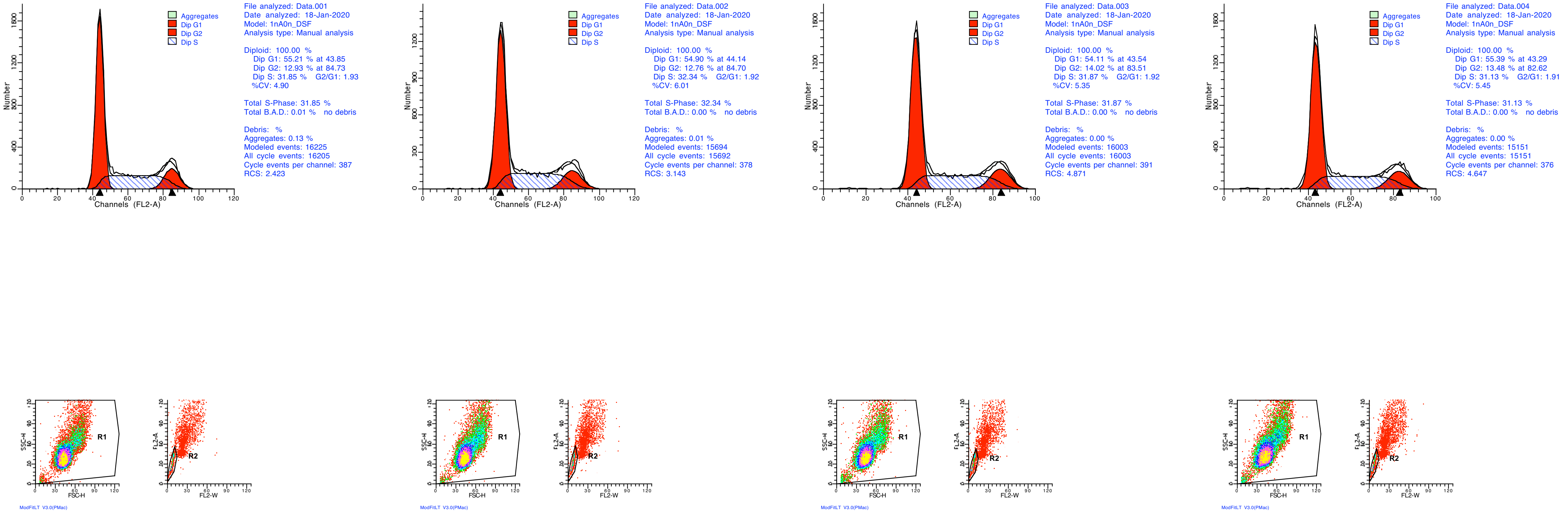

SGC-7901 Ctrl

Figure 2A

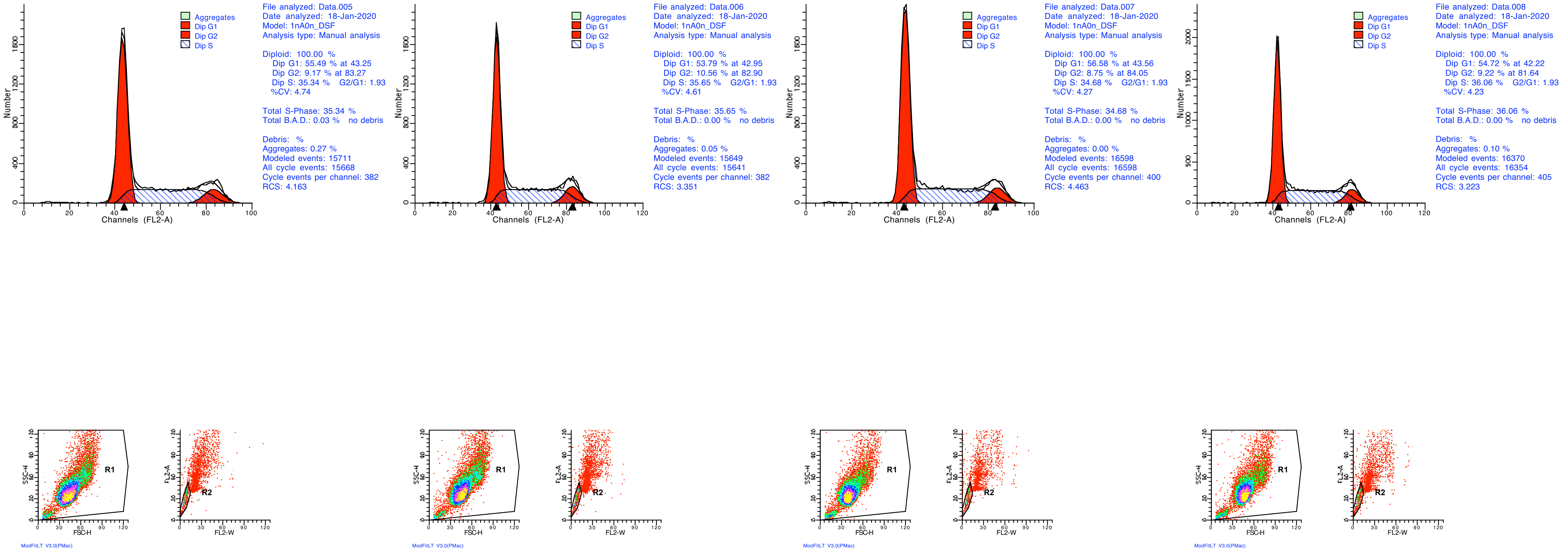

SGC-7901 BI

Figure 2A

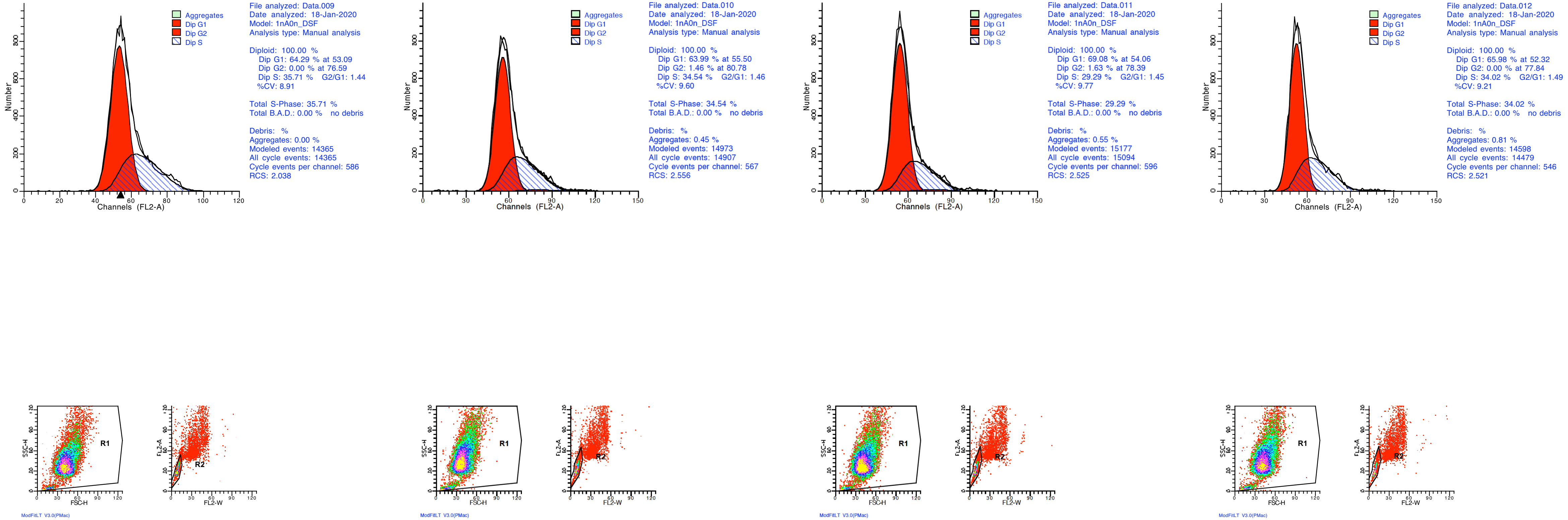

SGC-7901 5-FU

Figure 2A

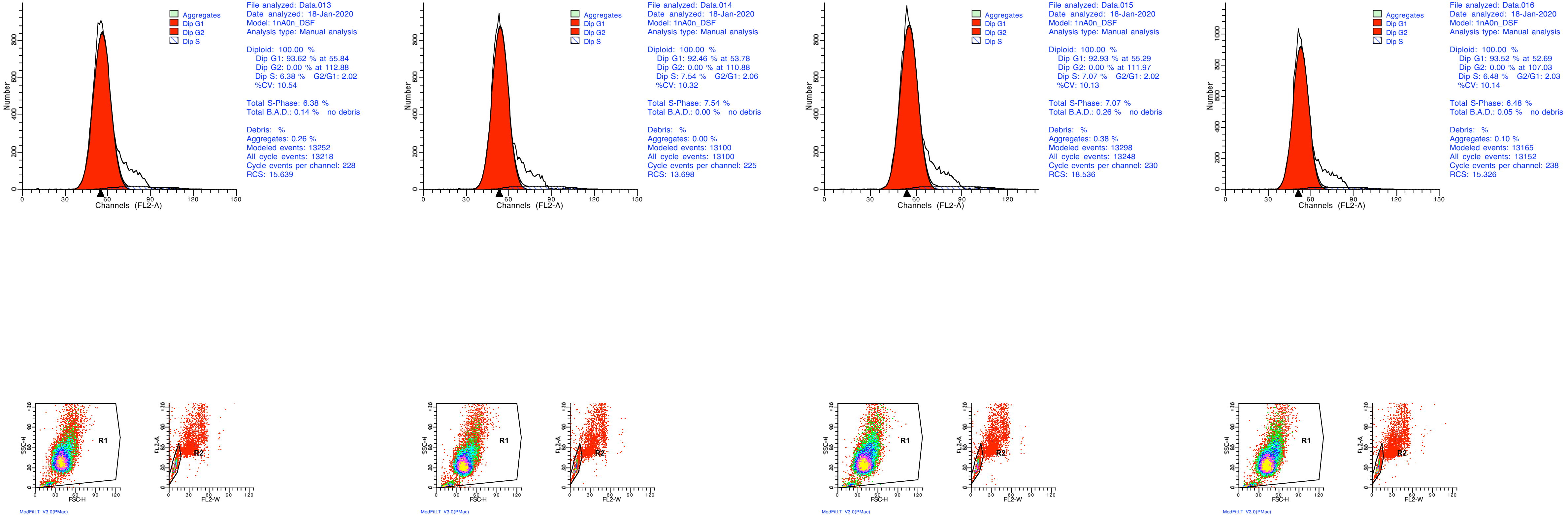

SGC-7901 BI+5-FU

Figure 2A

		G1	S	G2
Control	1	55.21%	31.85%	12.93%
	2	54.90%	32.34%	12.76%
	3	54.11%	31.87%	14.02%
	4	55.39%	31.13%	13.48%
	average	54.90%	31.80%	13.30%
	SD	0.01	0.00	0.01
		G1	S	G2
BI	1	55.49%	35.34%	9.17%
	2	53.79%	35.65%	10.56%
	3	56.58%	34.68%	8.75%
	4	54.72%	36.06%	9.22%
	average	55.15%	35.43%	9.43%
	SD	0.01	0.01	0.01
		G1	S	G2
	1	64.29%	35.71%	0.00%
	2	63.99%	34.54%	1.46%
5—FU	3	69.08%	29.29%	1.63%
	4	65.98%	34.02%	0.00%
	average	65.84%	33.39%	0.77%
	SD	0.02	0.03	0.01
		G1	S	G2
	1	93.62%	6.38%	0.00%
	2	92.46%	7.54%	0.00%
BI+5—FU	3	92.93%	7.07%	0.00%
	4	93.52%	6.48%	0.00%
	average	93.13%	6.87%	0.00%
	SD	0.01	0.01	0.00

Figure 2C MKN45 cells were harvested after treatment with BI (168 μmol/L) and 5-FU (0.9 μmol/L) alone or in combination for 72h.

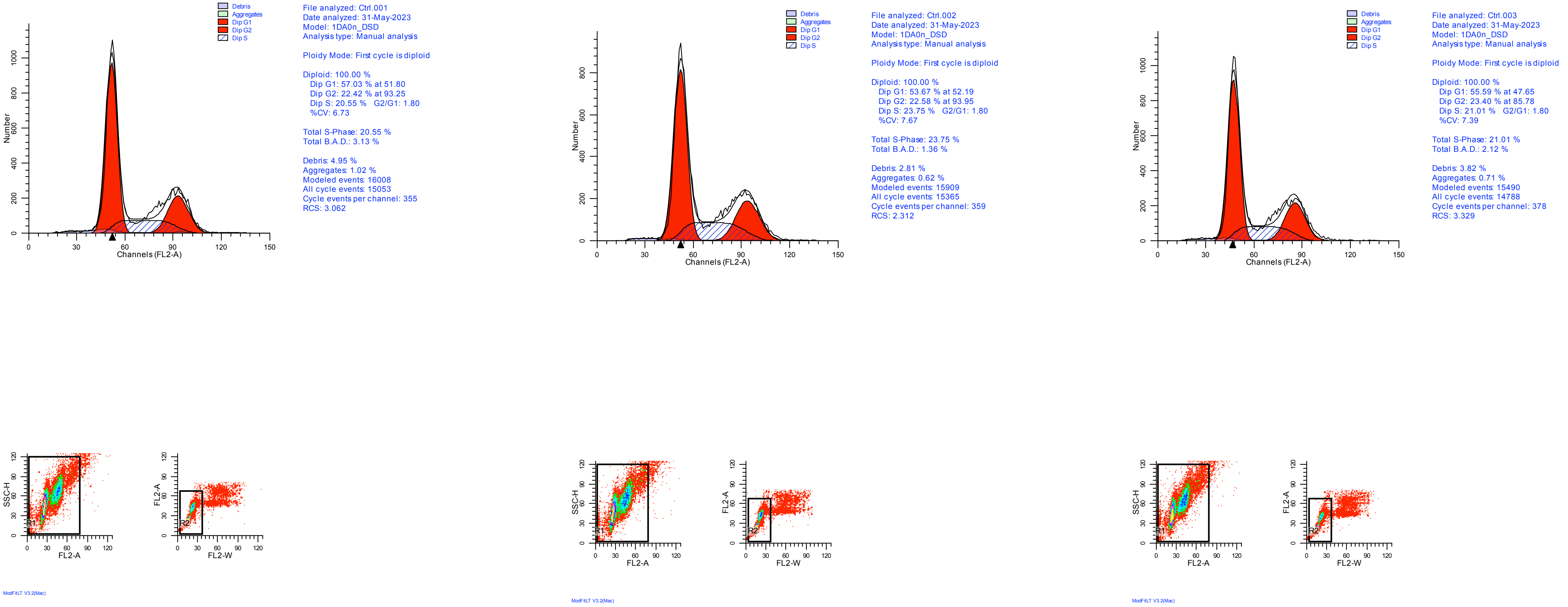

MKN45 Ctrl

Figure 2C

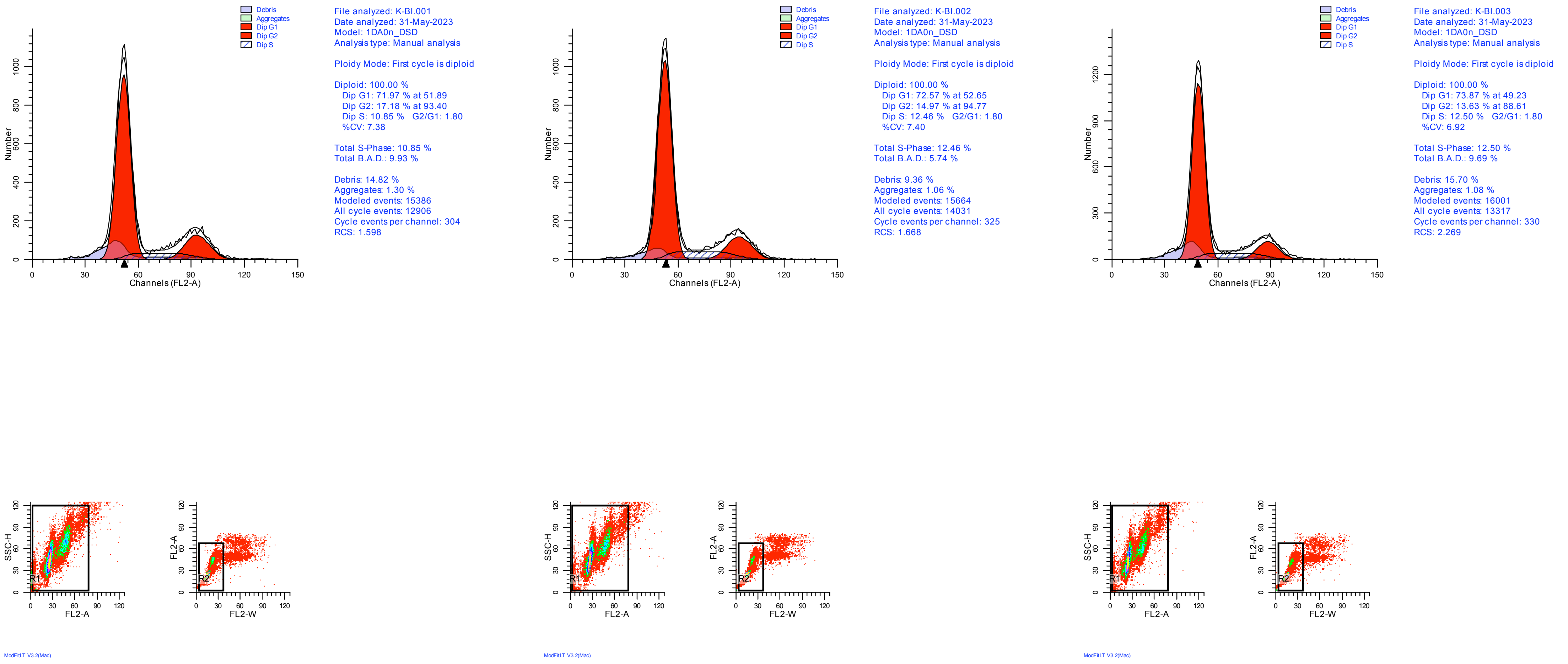

MKN45 BI

Figure 2C

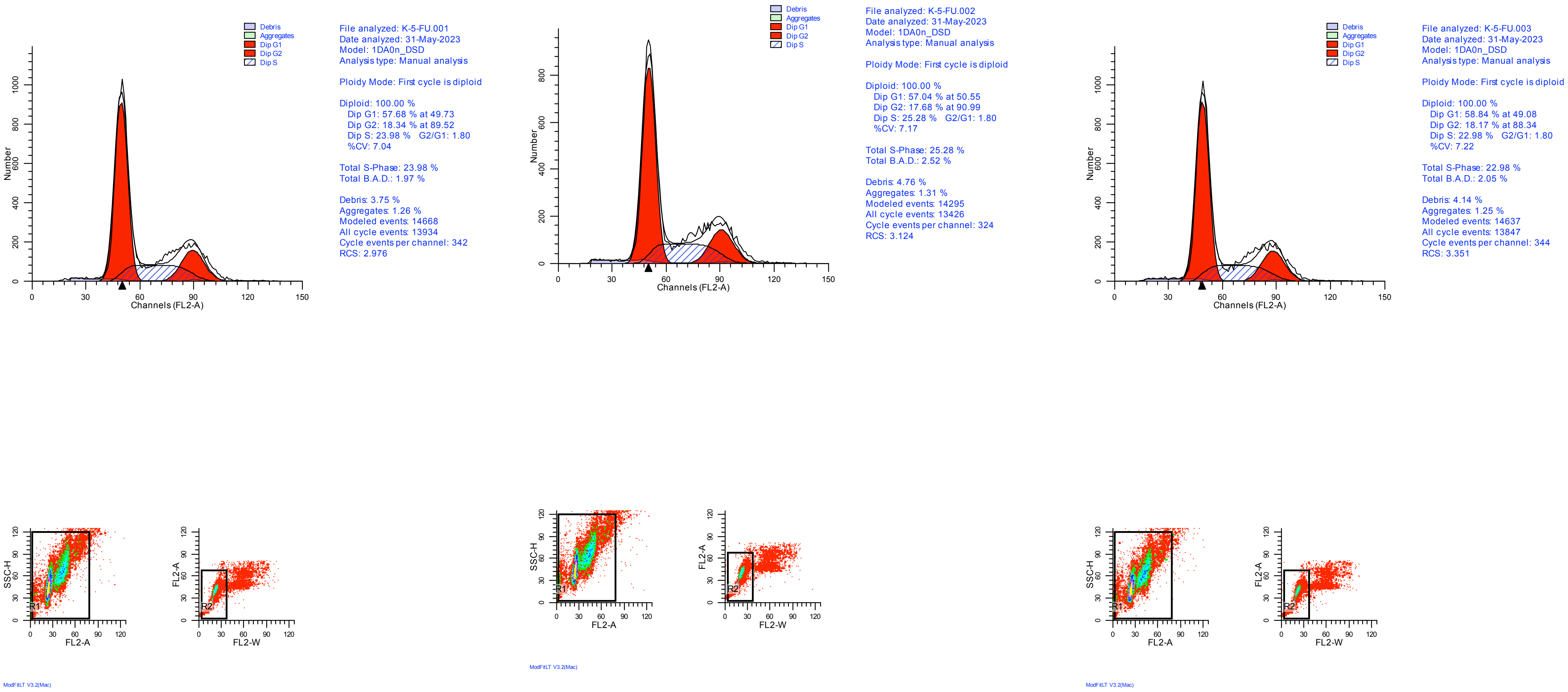

MKN45 5-FU

Figure 2C

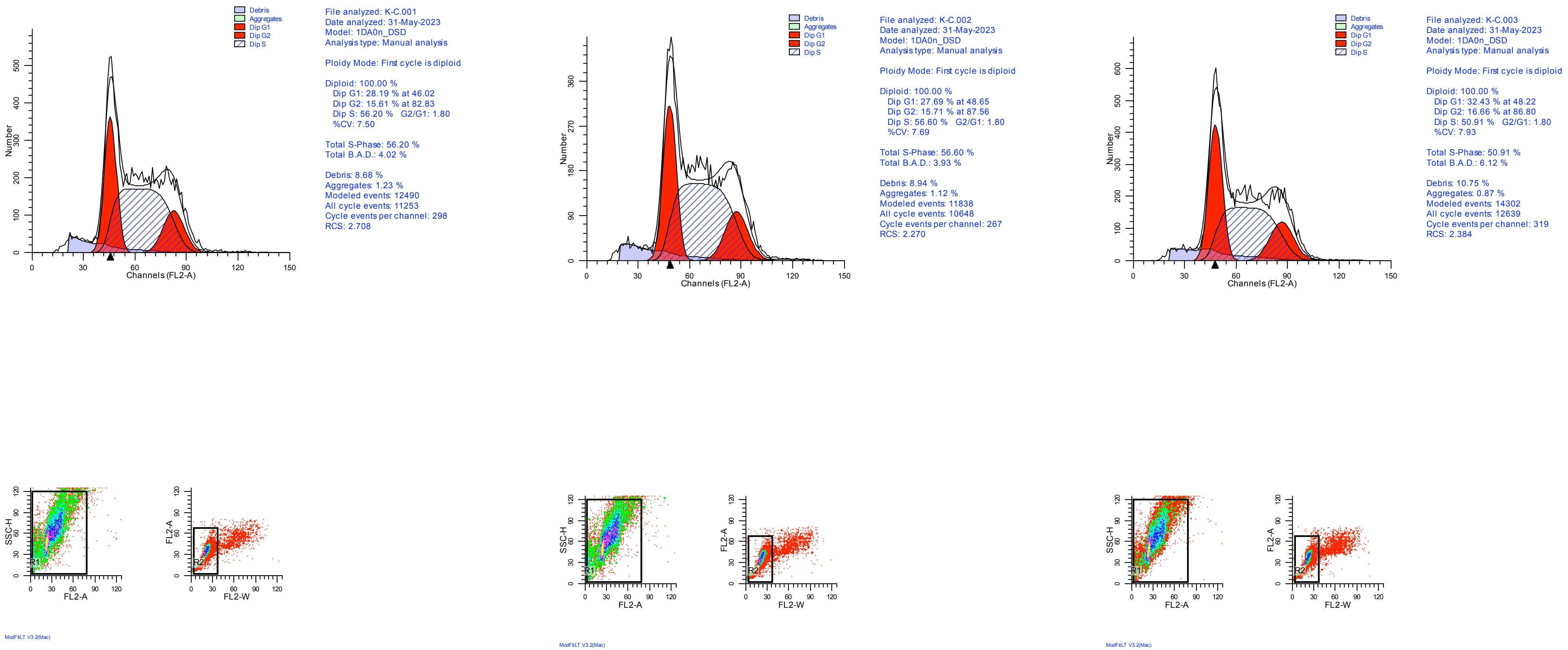

MKN45 BI+5-FU

Figure 2C

MKN45 CELL																
	G0/G1			S			G2/M				G0/G1		S		G2/M	
	1	2	3	1	2	3	1	2	3		MEAN	SD	MEAN	SD	MEAN	SD
CTRL	57.03%	53.67%	55.59%	20.55%	23.75%	21.01%	22.42%	22.58%	23.40%		55.43%	1.38%	21.77%	1.41%	22.80%	0.43%
BI	71.97%	72.57%	73.87%	10.85%	12.46%	12.50%	17.18%	14.97%	13.63%		72.80%	0.79%	11.94%	0.77%	15.26%	1.46%
5-FU	57.68%	57.04%	58.84%	23.98%	25.28%	22.98%	18.34%	17.68%	18.17%		57.85%	0.74%	24.08%	0.94%	18.06%	0.28%
C	28.19%	27.69%	32.43%	56.20%	56.60%	50.91%	15.61%	15.71%	16.66%		29.44%	2.13%	54.57%	2.59%	15.99%	0.47%

Figure 3A Apoptosis of SGC-7901 cells was detected by Annexin V-FITC/PI double staining assay after 6h of treatments with BI, 5-FU alone, or their combination.

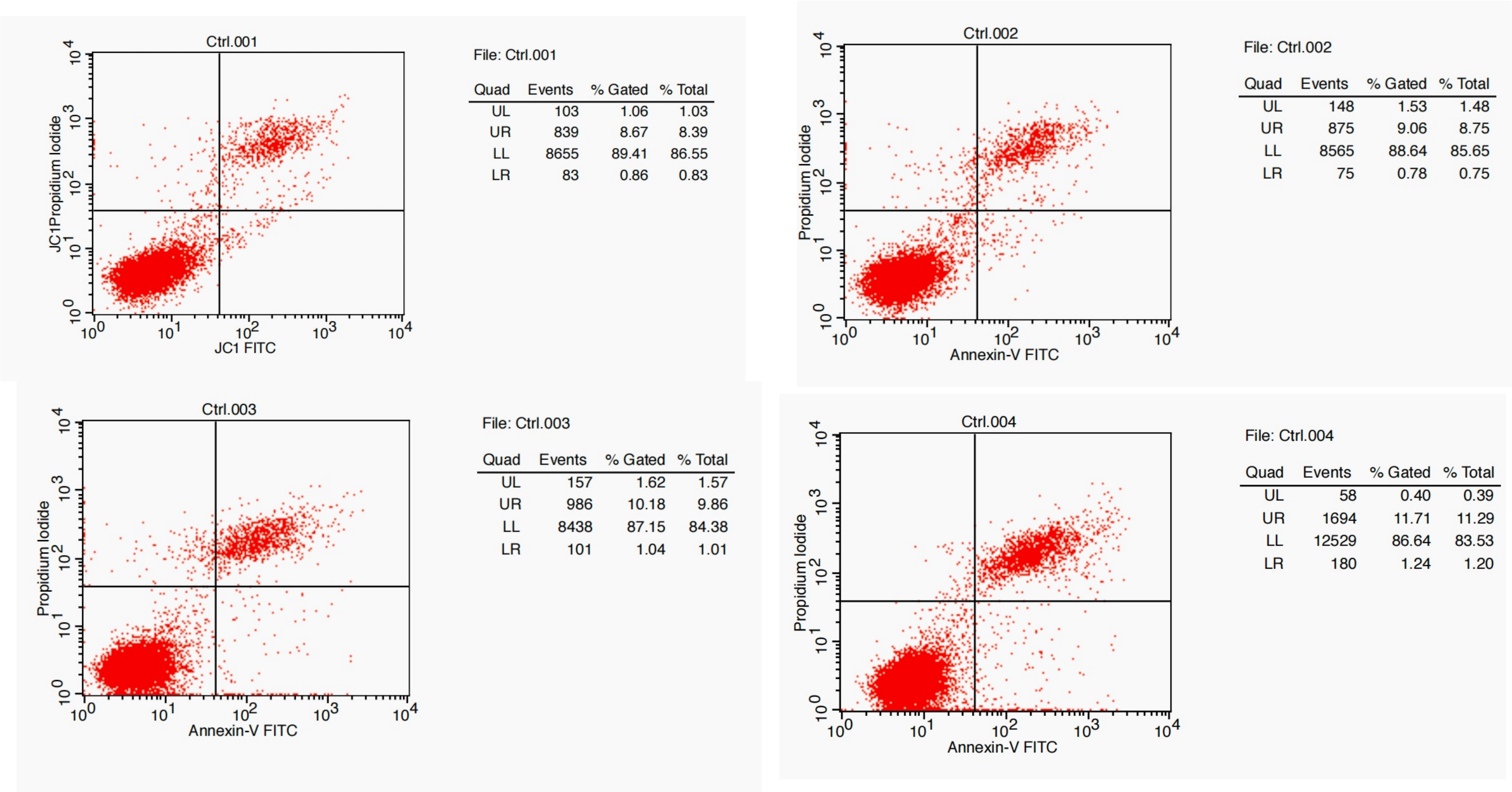

File: Ctrl.001

Quad	Events	% Gated	% Total
UL	103	1.06	1.03
UR	839	8.67	8.39
LL	8655	89.41	86.55
LR	83	0.86	0.83

File: Ctrl.002

Quad	Events	% Gated	% Total
UL	148	1.53	1.48
UR	875	9.06	8.75
LL	8565	88.64	85.65
LR	75	0.78	0.75

File: Ctrl.003

Quad	Events	% Gated	% Total
UL	157	1.62	1.57
UR	986	10.18	9.86
LL	8438	87.15	84.38
LR	101	1.04	1.01

File: Ctrl.004

Quad	Events	% Gated	% Total
UL	58	0.40	0.39
UR	1694	11.71	11.29
LL	12529	86.64	83.53
LR	180	1.24	1.20

SGC-7901 Ctrl

Figure 3A Apoptosis of SGC-7901 cells was detected by Annexin V-FITC/PI double staining assay after 6h of treatments with BI, 5-FU alone, or their combination.

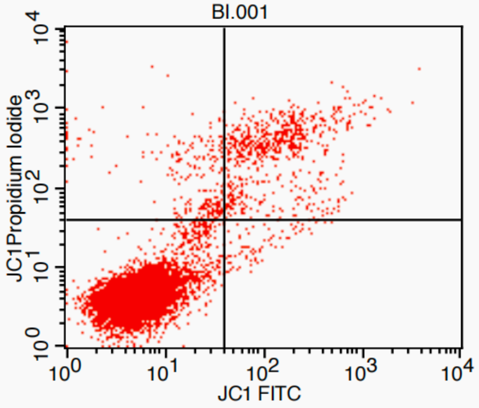

File: BI.001

Quad	Events	% Gated	% Total
UL	237	2.48	2.37
UR	673	7.03	6.73
LL	8588	89.71	85.88
LR	75	0.78	0.75

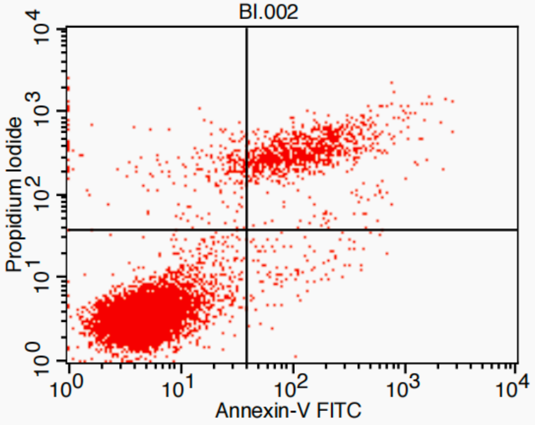

File: BI.002

Quad	Events	% Gated	% Total
UL	219	2.30	2.19
UR	951	9.98	9.51
LL	8264	86.73	82.64
LR	94	0.99	0.94

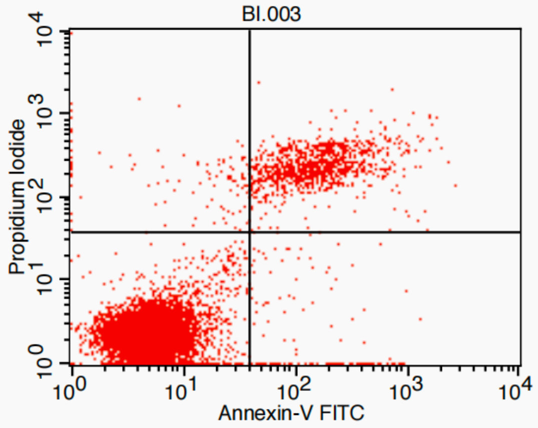

File: BI.003

Quad	Events	% Gated	% Total
UL	114	1.19	1.14
UR	1027	10.75	10.27
LL	8291	86.76	82.91
LR	124	1.30	1.24

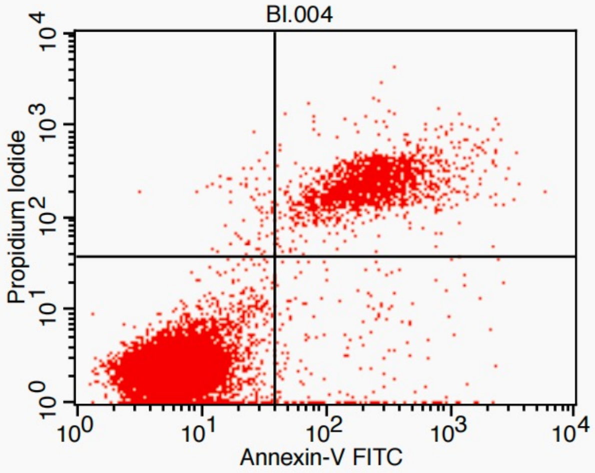

File: BI.004

Quad	Events	% Gated	% Total
UL	65	0.45	0.43
UR	1809	12.66	12.06
LL	12229	85.59	81.53
LR	185	1.29	1.23

SGC-7901 BI

Figure 3A Apoptosis of SGC-7901 cells was detected by Annexin V-FITC/PI double staining assay after 6h of treatments with BI, 5-FU alone, or their combination.

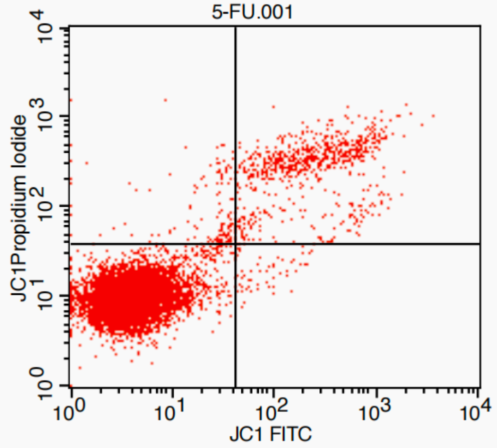

File: 5-FU.001

Quad	Events	% Gated	% Total
UL	129	1.31	1.29
UR	714	7.26	7.14
LL	8931	90.83	89.31
LR	59	0.60	0.59

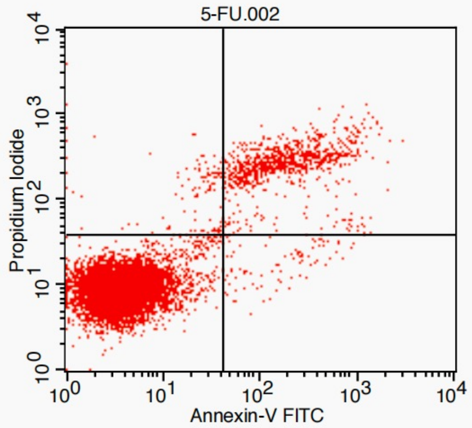

File: 5-FU.002

Quad	Events	% Gated	% Total
UL	112	1.14	1.12
UR	883	9.01	8.83
LL	8729	89.04	87.29
LR	80	0.82	0.80

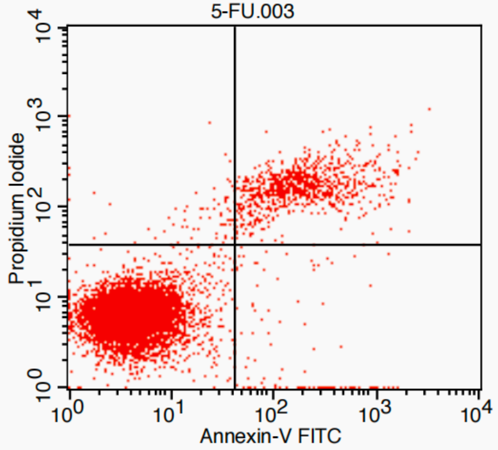

File: 5-FU.003

Quad	Events	% Gated	% Total
UL	67	0.68	0.67
UR	919	9.38	9.19
LL	8682	88.65	86.82
LR	126	1.29	1.26

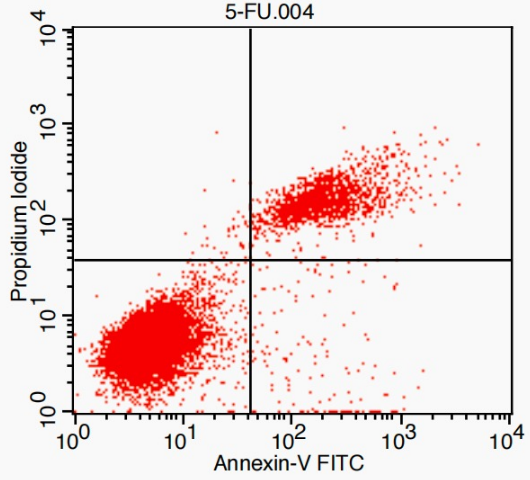

File: 5-FU.004

Quad	Events	% Gated	% Total
UL	39	0.27	0.26
UR	1528	10.43	10.19
LL	12933	88.27	86.22
LR	151	1.03	1.01

SGC-7901 5-FU

Figure 3A Apoptosis of SGC-7901 cells was detected by Annexin V-FITC/PI double staining assay after 6h of treatments with BI, 5-FU alone, or their combination.

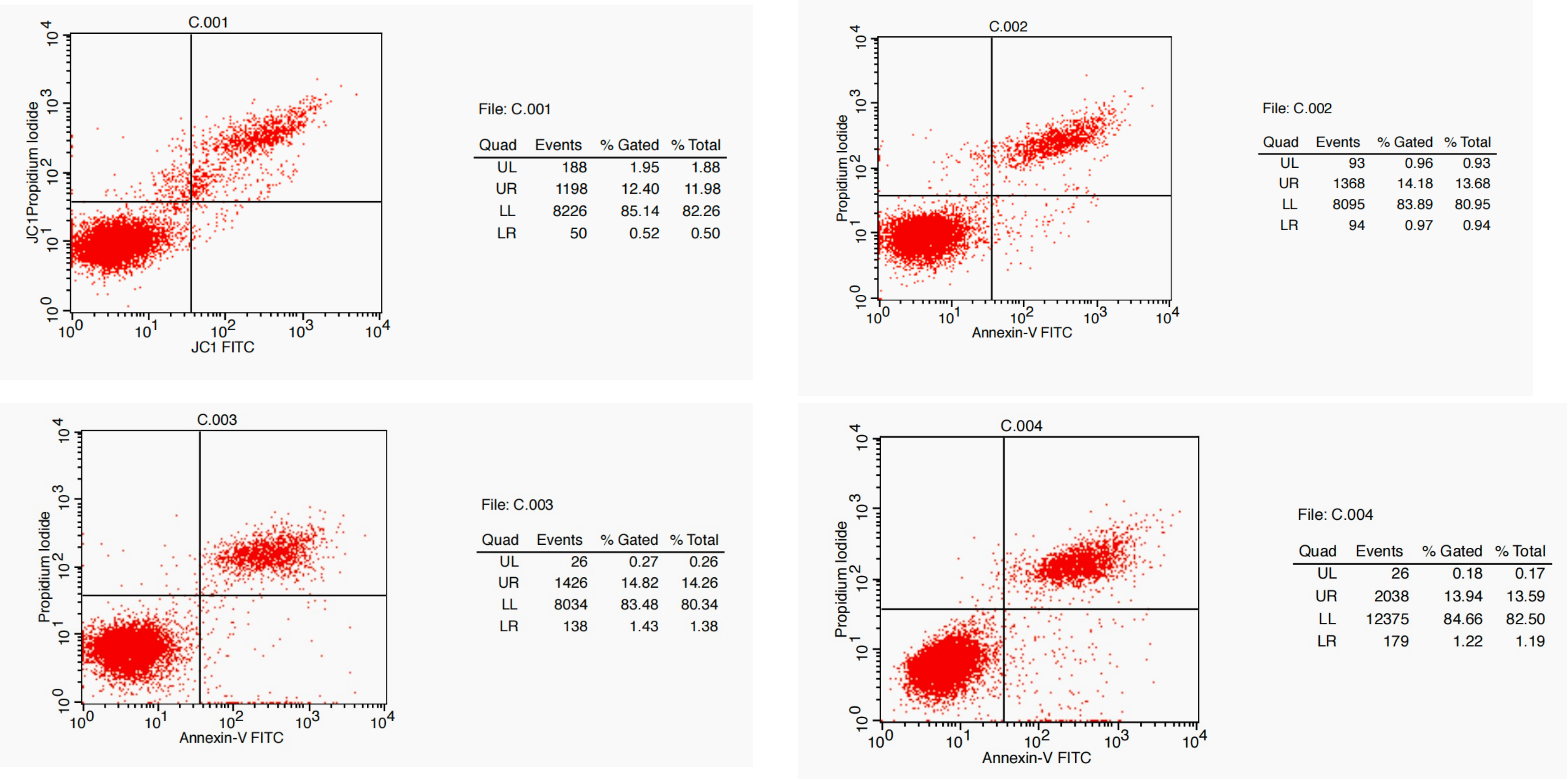

SGC-7901 BI+5-FU

Figure 3A Apoptosis of SGC-7901 cells was detected by Annexin V-FITC/PI double staining assay after 6h of treatments with BI, 5-FU alone, or their combination.

7901	6h	右下	右上	总
	ctrl1	0.83	8.39	9.22
	ctrl2	0.75	8.75	9.5
	ctrl3	1.01	9.86	10.87
	ctrl4	1.2	11.29	12.49
	BI1	0.75	6.73	7.48
	BI2	0.94	9.51	10.45
	BI3	1.24	10.27	11.51
	BI4	1.23	12.06	13.29
	5—FU1	0.59	7.14	7.73
	5—FU2	0.8	8.83	9.63
	5—FU3	1.26	9.19	10.45
	5—FU4	1.01	10.19	11.2
	C1	0.58	11.98	12.56
	C2	0.94	13.68	14.62
	C3	1.38	14.26	15.64
	C4	1.19	13.59	14.78

Figure 3B Apoptosis of SGC-7901 cells was detected by Annexin V-FITC/PI double staining assay after 12h of treatments with BI, 5-FU alone, or their combination.

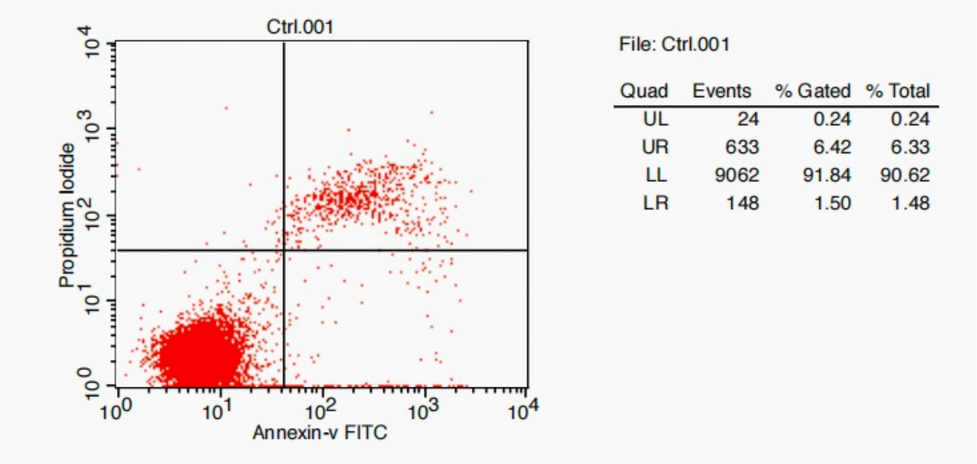

Quad	Events	% Gated	% Total
UL	24	0.24	0.24
UR	633	6.42	6.33
LL	9062	91.84	90.62
LR	148	1.50	1.48

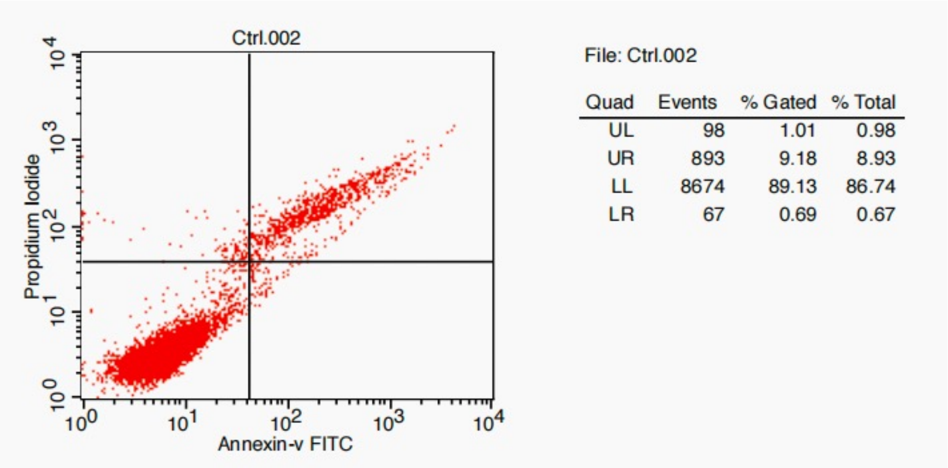

Quad	Events	% Gated	% Total
UL	98	1.01	0.98
UR	893	9.18	8.93
LL	8674	89.13	86.74
LR	67	0.69	0.67

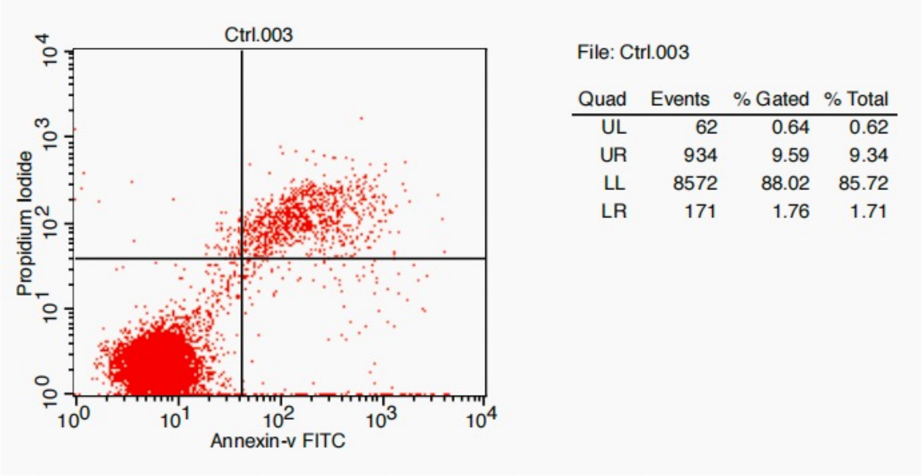

Quad	Events	% Gated	% Total
UL	62	0.64	0.62
UR	934	9.59	9.34
LL	8572	88.02	85.72
LR	171	1.76	1.71

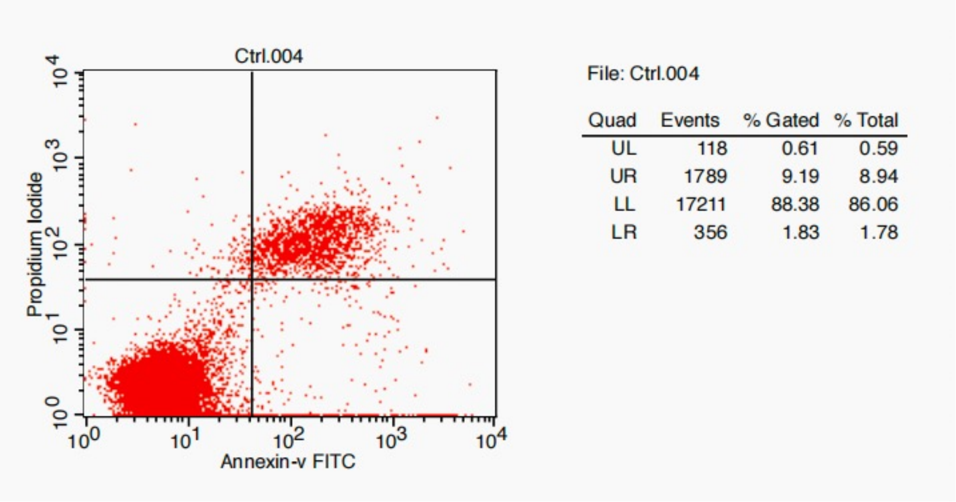

Quad	Events	% Gated	% Total
UL	118	0.61	0.59
UR	1789	9.19	8.94
LL	17211	88.38	86.06
LR	356	1.83	1.78

SGC-7901 Ctrl

Figure 3B Apoptosis of SGC-7901 cells was detected by Annexin V-FITC/PI double staining assay after 12h of treatments with BI, 5-FU alone, or their combination.

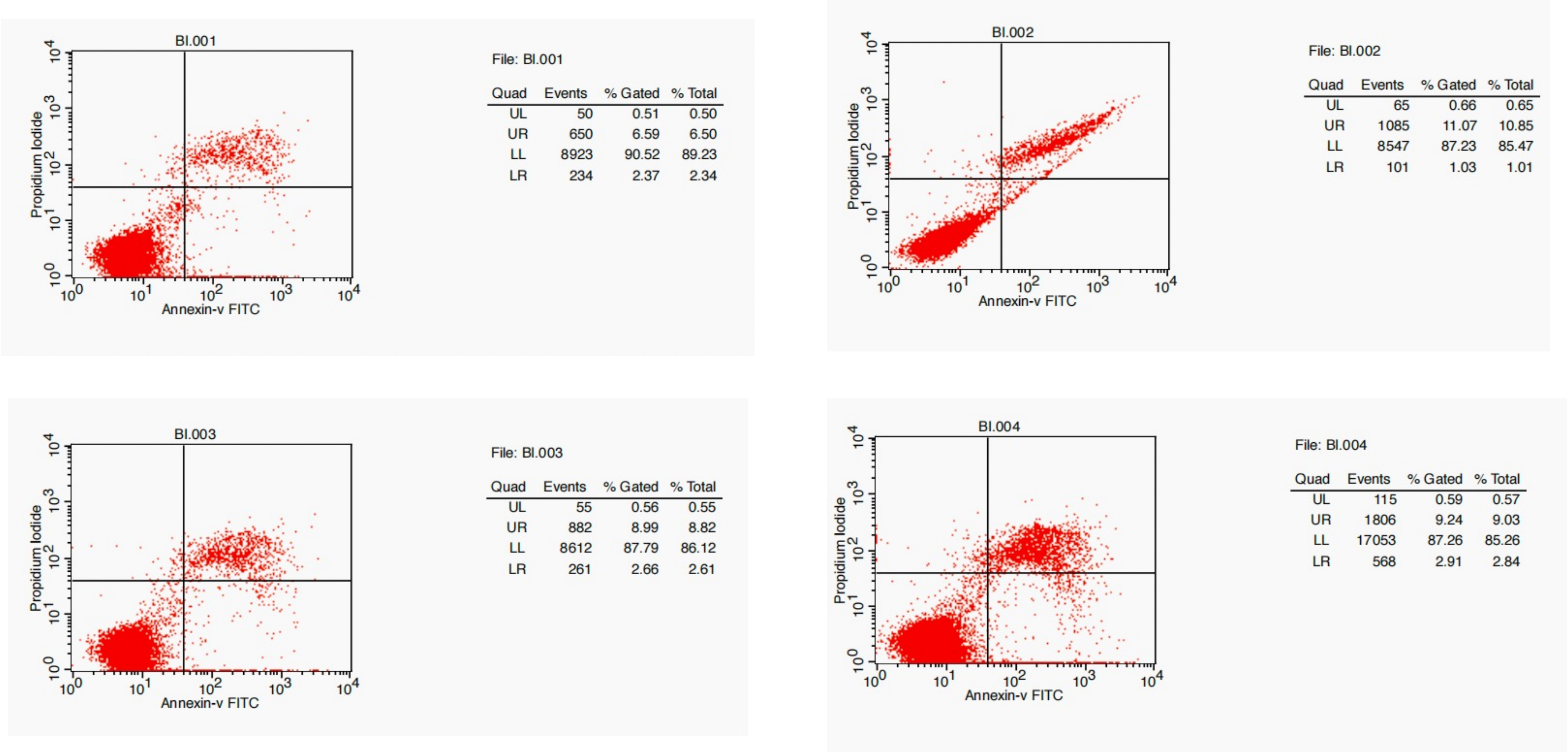

File: BI.001

Quad	Events	% Gated	% Total
UL	50	0.51	0.50
UR	650	6.59	6.50
LL	8923	90.52	89.23
LR	234	2.37	2.34

File: BI.002

Quad	Events	% Gated	% Total
UL	65	0.66	0.65
UR	1085	11.07	10.85
LL	8547	87.23	85.47
LR	101	1.03	1.01

File: BI.003

Quad	Events	% Gated	% Total
UL	55	0.56	0.55
UR	882	8.99	8.82
LL	8612	87.79	86.12
LR	261	2.66	2.61

File: BI.004

Quad	Events	% Gated	% Total
UL	115	0.59	0.57
UR	1806	9.24	9.03
LL	17053	87.26	85.26
LR	568	2.91	2.84

SGC-7901 BI

Figure 3B Apoptosis of SGC-7901 cells was detected by Annexin V-FITC/PI double staining assay after 12h of treatments with BI, 5-FU alone, or their combination.

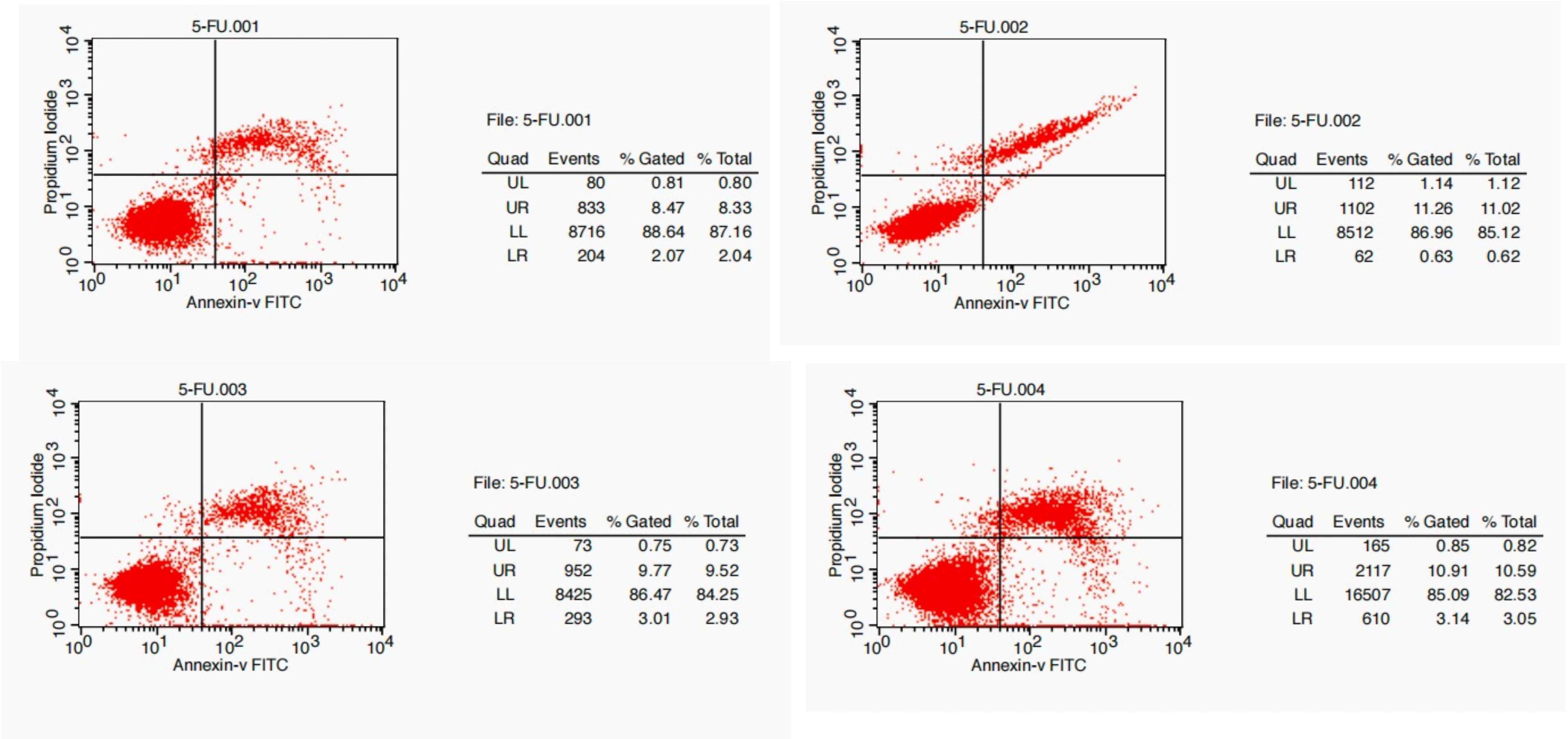

File: 5-FU.001

Quad	Events	% Gated	% Total
UL	80	0.81	0.80
UR	833	8.47	8.33
LL	8716	88.64	87.16
LR	204	2.07	2.04

File: 5-FU.002

Quad	Events	% Gated	% Total
UL	112	1.14	1.12
UR	1102	11.26	11.02
LL	8512	86.96	85.12
LR	62	0.63	0.62

File: 5-FU.003

Quad	Events	% Gated	% Total
UL	73	0.75	0.73
UR	952	9.77	9.52
LL	8425	86.47	84.25
LR	293	3.01	2.93

File: 5-FU.004

Quad	Events	% Gated	% Total
UL	165	0.85	0.82
UR	2117	10.91	10.59
LL	16507	85.09	82.53
LR	610	3.14	3.05

SGC-7901 5-FU

Figure 3B Apoptosis of SGC-7901 cells was detected by Annexin V-FITC/PI double staining assay after 12h of treatments with BI, 5-FU alone, or their combination.

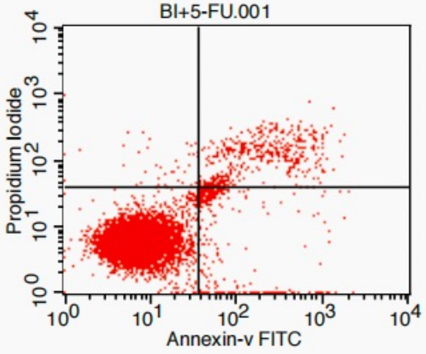

File: BI+5-FU.001

Quad	Events	% Gated	% Total
UL	37	0.38	0.37
UR	432	4.39	4.32
LL	9034	91.78	90.34
LR	340	3.45	3.40

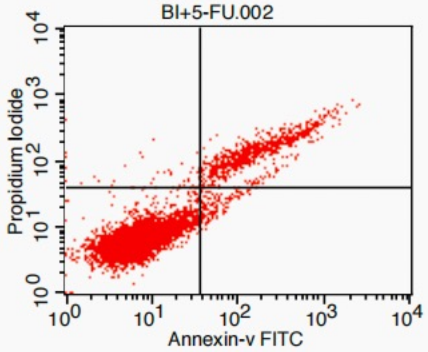

File: BI+5-FU.002

Quad	Events	% Gated	% Total
UL	38	0.39	0.38
UR	804	8.20	8.04
LL	8816	89.89	88.16
LR	150	1.53	1.50

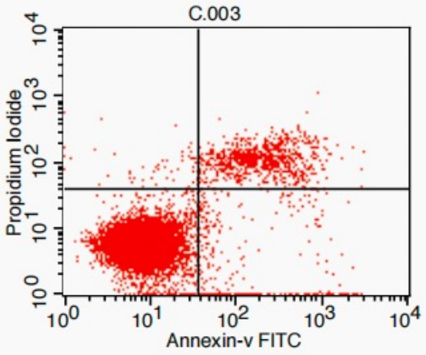

File: C.003

Quad	Events	% Gated	% Total
UL	52	0.53	0.52
UR	833	8.49	8.33
LL	8639	88.05	86.39
LR	288	2.94	2.88

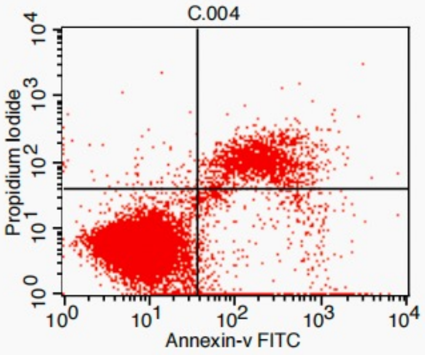

File: C.004

Quad	Events	% Gated	% Total
UL	80	0.41	0.40
UR	1657	8.45	8.28
LL	17203	87.74	86.01
LR	666	3.40	3.33

SGC-7901 BI+5-FU

Figure 3B Apoptosis of SGC-7901 cells was detected by Annexin V-FITC/PI double staining assay after 12h of treatments with BI, 5-FU alone, or their combination.

7901	12h	右下	右上	总
	ctrl1	1.48	6.33	7.81
	ctrl2	8.93	0.67	9.6
	ctrl3	9.34	1.71	11.05
	ctrl4	8.94	1.78	10.72
	BI1	2.34	6.5	8.84
	BI2	10.85	1.01	11.86
	BI3	8.82	2.61	11.43
	BI4	9.03	2.84	11.87
	5—FU1	2.04	8.33	10.37
	5—FU2	11.02	0.62	11.64
	5—FU3	9.52	2.93	12.45
	5—FU4	10.59	3.05	13.64
	C1	3.4	4.32	7.72
	C2	8.04	1.5	9.54
	C3	8.33	2.88	11.21
	C4	8.28	3.33	11.61

Figure 3C Apoptosis of SGC-7901 cells was detected by Annexin V-FITC/PI double staining assay after 24h of treatments with BI, 5-FU alone, or their combination.

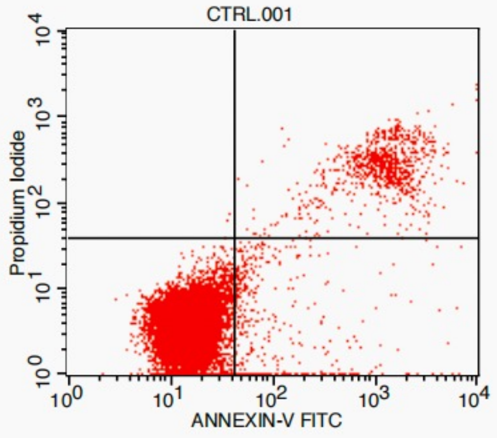

File: CTRL.001

Quad	Events	% Gated	% Total
UL	2	0.01	0.01
UR	838	5.67	5.59
LL	13508	91.39	90.05
LR	432	2.92	2.88

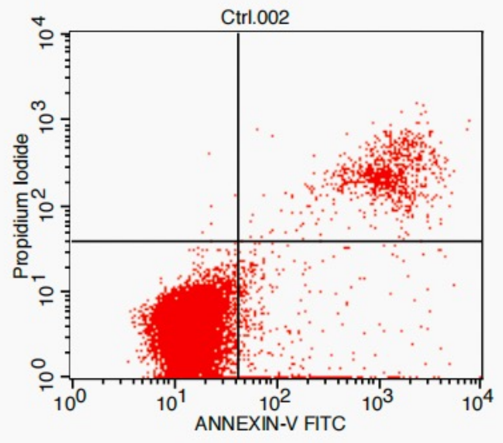

File: Ctrl.002

Quad	Events	% Gated	% Total
UL	6	0.04	0.04
UR	860	5.82	5.73
LL	13465	91.16	89.77
LR	439	2.97	2.93

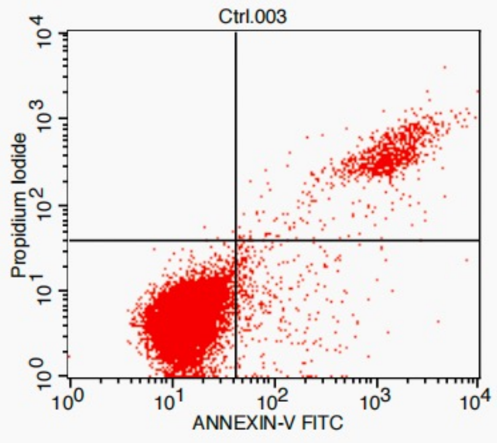

File: Ctrl.003

Quad	Events	% Gated	% Total
UL	3	0.02	0.02
UR	946	6.42	6.31
LL	13433	91.18	89.55
LR	350	2.38	2.33

SGC-7901 Ctrl

Figure 3C Apoptosis of SGC-7901 cells was detected by Annexin V-FITC/PI double staining assay after 24h of treatments with BI, 5-FU alone, or their combination.

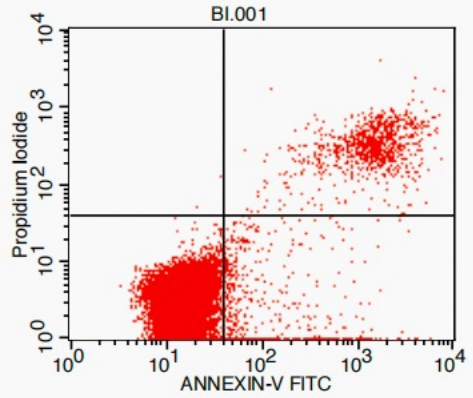

File: BI.001

Quad	Events	% Gated	% Total
UL	2	0.01	0.01
UR	980	6.63	6.53
LL	13226	89.52	88.17
LR	567	3.84	3.78

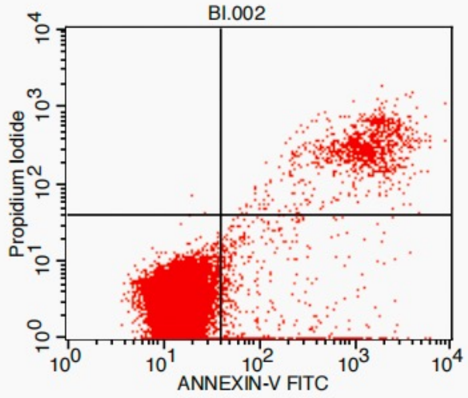

File: BI.002

Quad	Events	% Gated	% Total
UL	3	0.02	0.02
UR	1024	6.94	6.83
LL	13175	89.26	87.83
LR	558	3.78	3.72

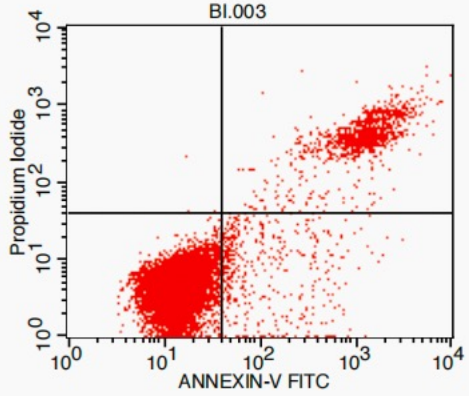

File: BI.003

Quad	Events	% Gated	% Total
UL	3	0.02	0.02
UR	1106	7.50	7.37
LL	13169	89.32	87.79
LR	465	3.15	3.10

SGC-7901 BI

Figure 3C Apoptosis of SGC-7901 cells was detected by Annexin V-FITC/PI double staining assay after 24h of treatments with BI, 5-FU alone, or their combination.

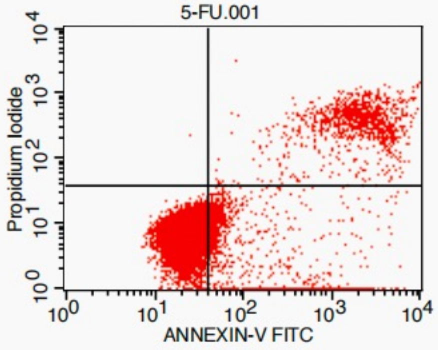

File: 5-FU.001

Quad	Events	% Gated	% Total
UL	1	0.01	0.01
UR	1081	7.31	7.21
LL	12499	84.47	83.33
LR	1216	8.22	8.11

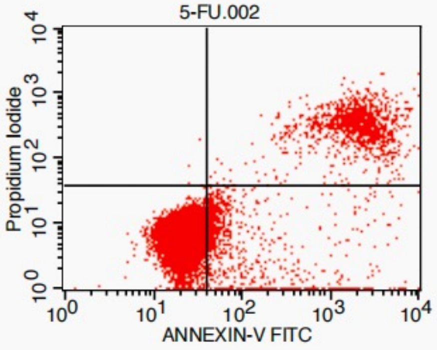

File: 5-FU.002

Quad	Events	% Gated	% Total
UL	3	0.02	0.02
UR	1148	7.78	7.65
LL	12491	84.67	83.27
LR	1110	7.52	7.40

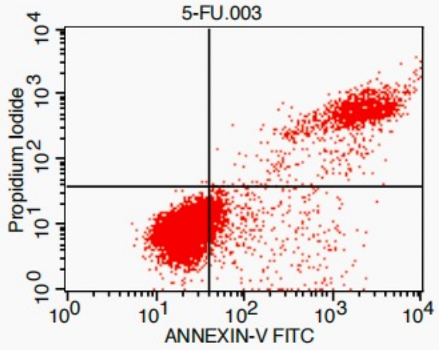

File: 5-FU.003

Quad	Events	% Gated	% Total
UL	2	0.01	0.01
UR	1269	8.63	8.46
LL	12582	85.52	83.88
LR	859	5.84	5.73

SGC-7901 5-FU

Figure 3C Apoptosis of SGC-7901 cells was detected by Annexin V-FITC/PI double staining assay after 24h of treatments with BI, 5-FU alone, or their combination.

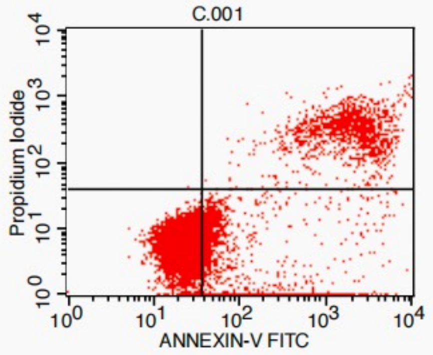

File: C.001

Quad	Events	% Gated	% Total
UL	1	0.01	0.01
UR	1246	8.46	8.31
LL	11520	78.26	76.80
LR	1954	13.27	13.03

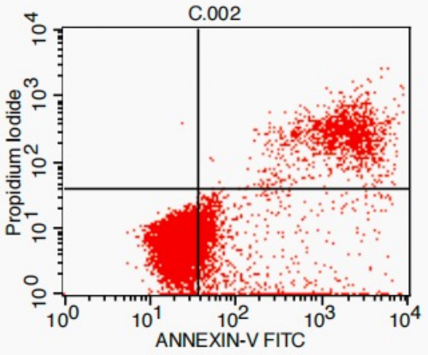

File: C.002

Quad	Events	% Gated	% Total
UL	1	0.01	0.01
UR	1269	8.68	8.46
LL	11525	78.81	76.83
LR	1828	12.50	12.19

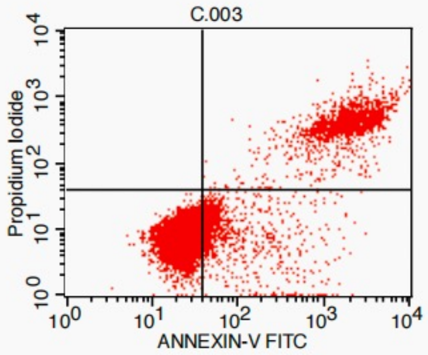

File: C.003

Quad	Events	% Gated	% Total
UL	0	0.00	0.00
UR	1516	10.39	10.11
LL	11663	79.92	77.75
LR	1415	9.70	9.43

SGC-7901 BI+5-FU

Figure 3C Apoptosis of SGC-7901 cells was detected by Annexin V-FITC/PI double staining assay after 24h of treatments with BI, 5-FU alone, or their combination.

7901	24h	右下	右上	总
	ctrl1	2.88	5.59	8.47
	ctrl2	2.93	5.73	8.66
	ctrl3	2.33	6.31	8.64
	ctrl4			0
	BI1	3.78	6.53	10.31
	BI2	3.72	6.83	10.55
	BI3	3.1	7.37	10.47
	BI4			0
	5—FU1	8.11	7.21	15.32
	5—FU2	7.4	7.65	15.05
	5—FU3	5.73	8.46	14.19
	5—FU4			0
	C1	13.03	8.31	21.34
	C2	12.19	8.46	20.65
	C3	9.43	10.11	19.54
	C4			0

Figure 3D The changes of mitochondrial membrane potential (MMP) in SGC-7901 cells were detected by RH-123 staining after treatment with BI, 5-FU alone or their combination for 6 h

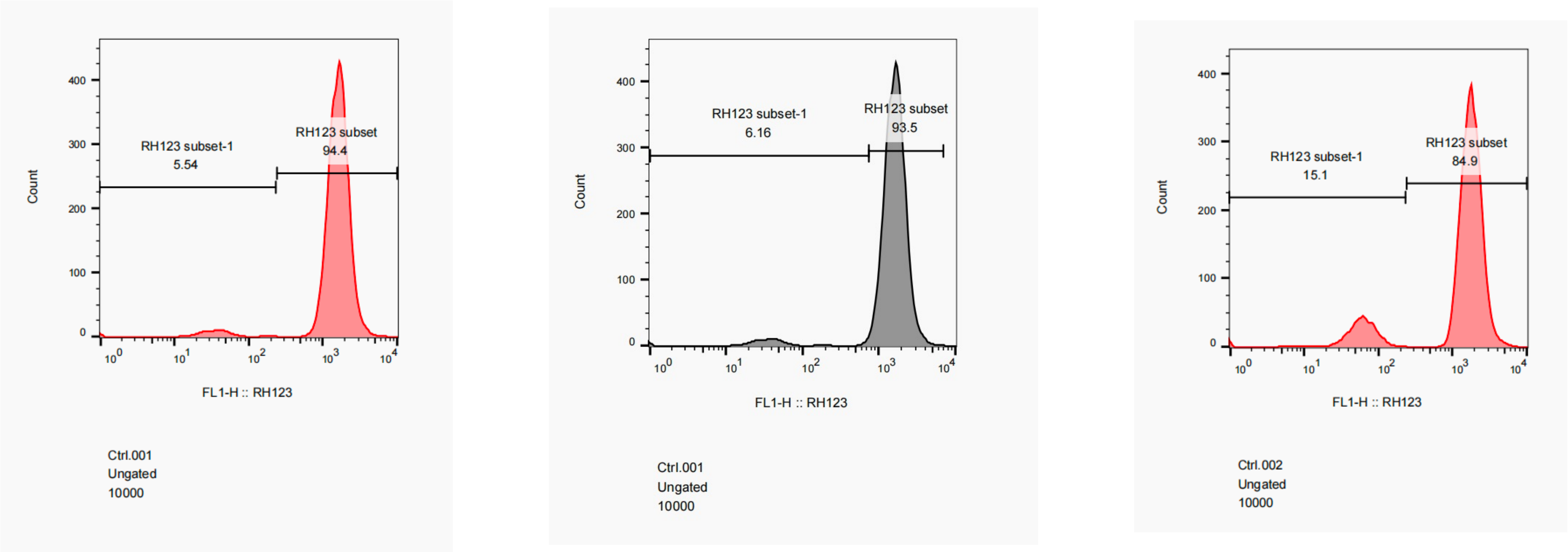

SGC-7901 Ctrl

Figure 3D The changes of mitochondrial membrane potential (MMP) in SGC-7901 cells were detected by RH-123 staining after treatment with BI, 5-FU alone or their combination for 6 h

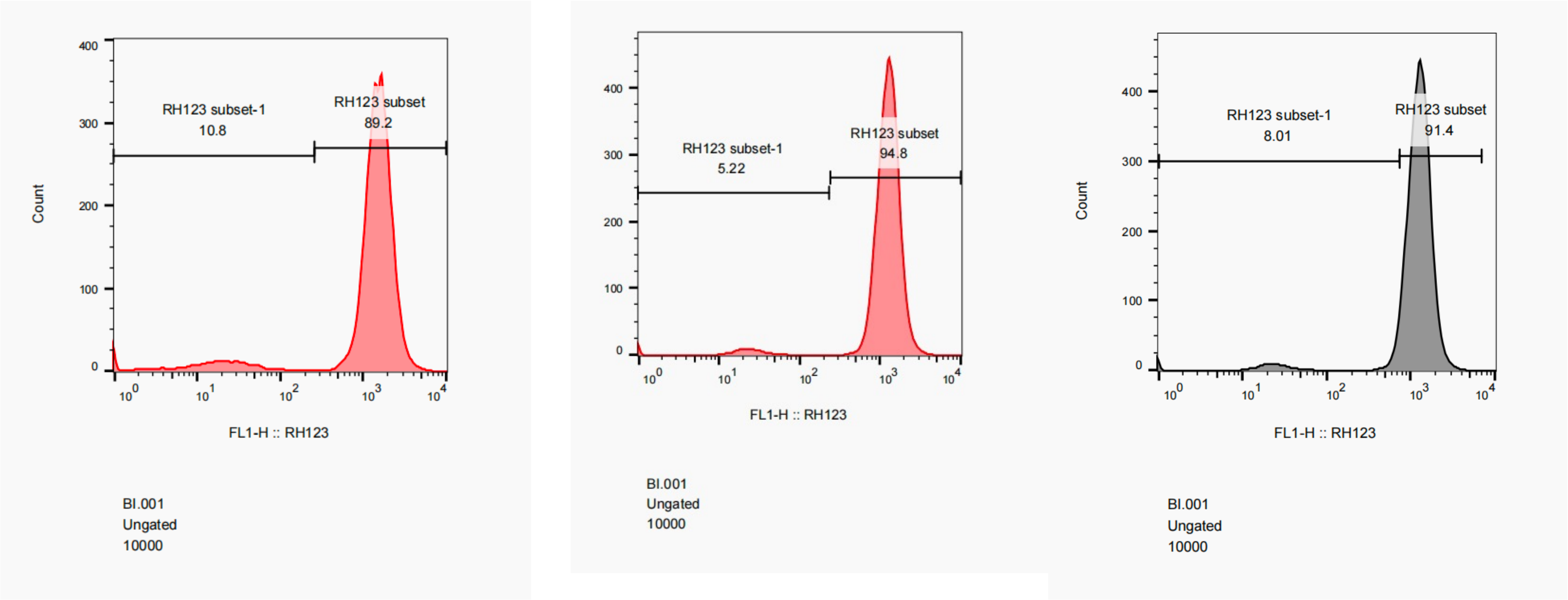

SGC-7901 BI

Figure 3D The changes of mitochondrial membrane potential (MMP) in SGC-7901 cells were detected by RH-123 staining after treatment with BI, 5-FU alone or their combination for 6 h

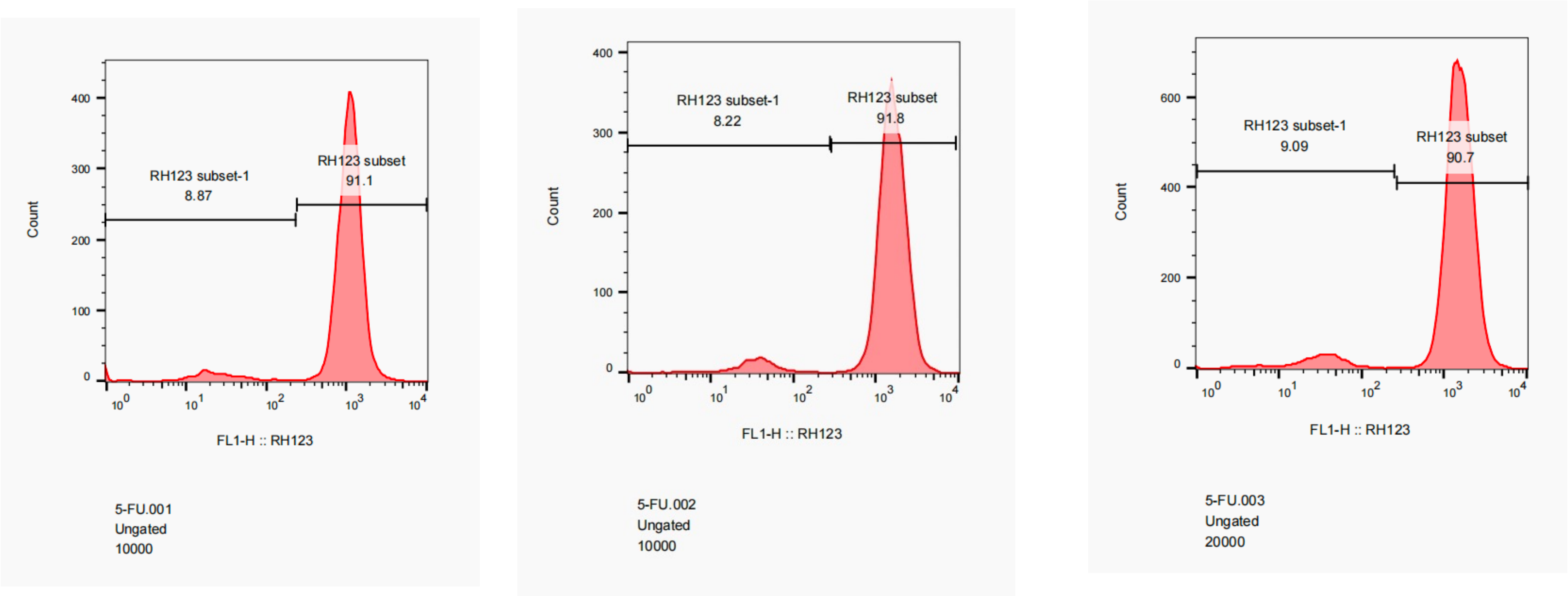

SGC-7901 5-FU

Figure 3D The changes of mitochondrial membrane potential (MMP) in SGC-7901 cells were detected by RH-123 staining after treatment with BI, 5-FU alone or their combination for 6 h

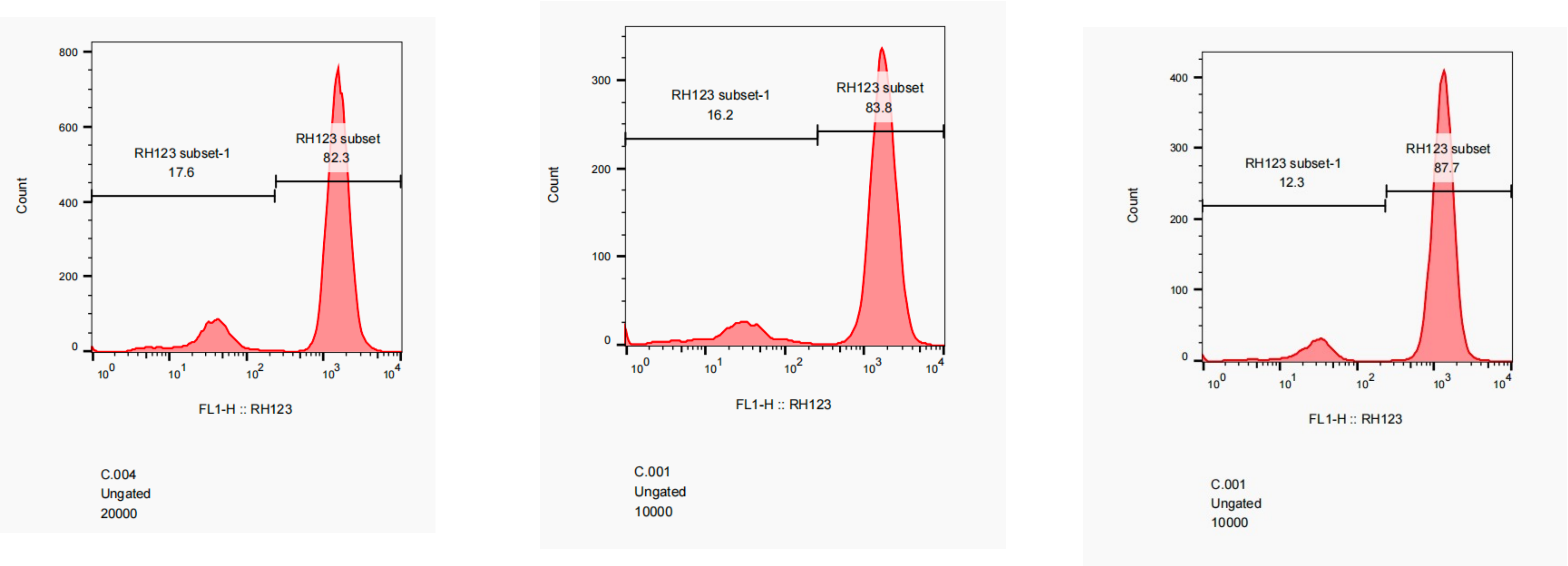

SGC-7901 BI+5-FU

Figure 3D The changes of mitochondrial membrane potential (MMP) in SGC-7901 cells were detected by RH-123 staining after treatment with BI, 5-FU alone or their combination for 6 h

ctrl	BI	5-FU	BI+5-FU
93.5	91.4	91.8	83.8
94.4	94.8	91.1	87.8
84.9	89.2	90.7	82.3

Figure 3E The changes of mitochondrial membrane potential (MMP) in SGC-7901 cells were detected by RH-123 staining after treatment with BI, 5-FU alone or their combination for 12 h

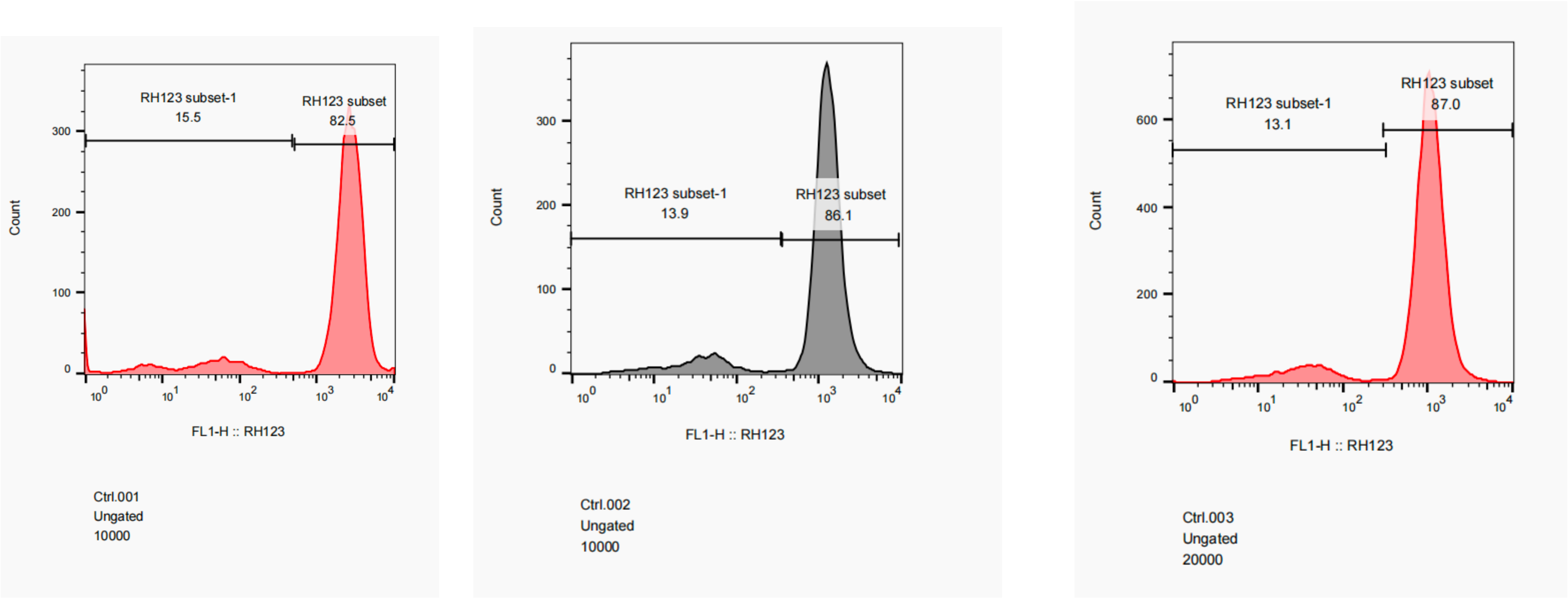

SGC-7901 Ctrl

Figure 3E The changes of mitochondrial membrane potential (MMP) in SGC-7901 cells were detected by RH-123 staining after treatment with BI, 5-FU alone or their combination for 12 h

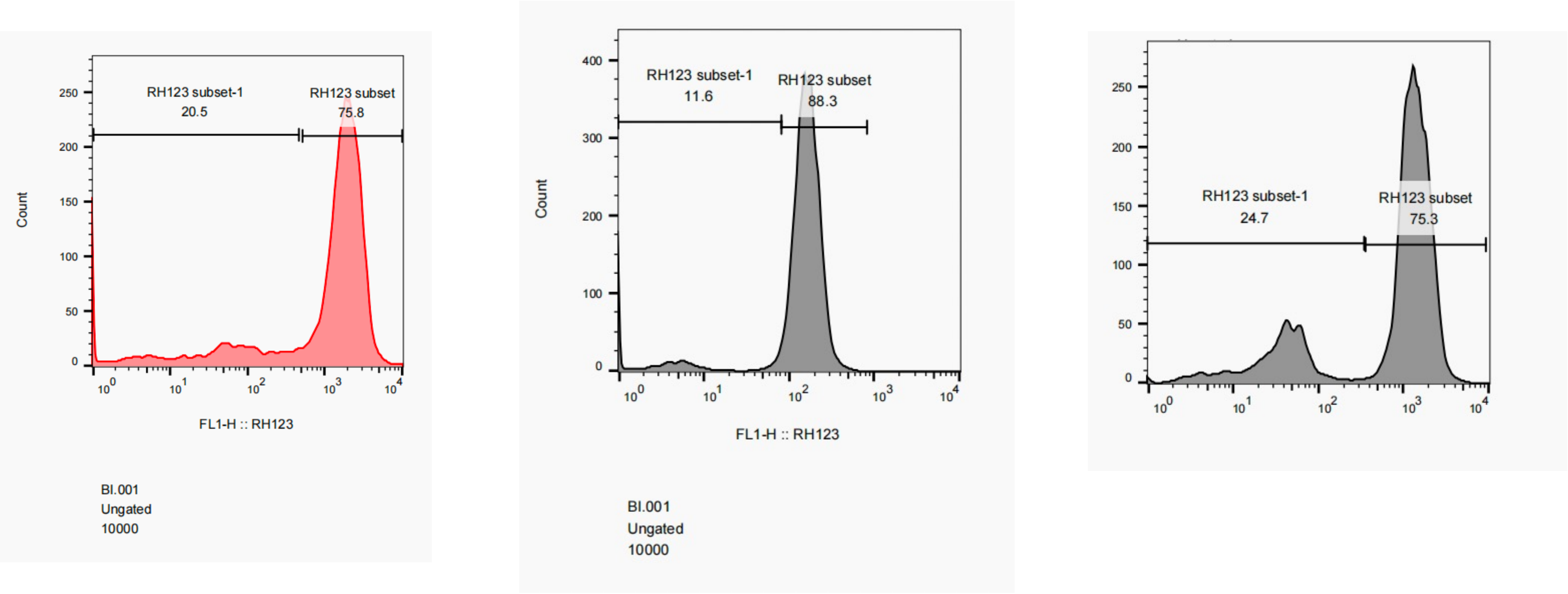

SGC-7901 BI

Figure 3E The changes of mitochondrial membrane potential (MMP) in SGC-7901 cells were detected by RH-123 staining after treatment with BI, 5-FU alone or their combination for 12 h

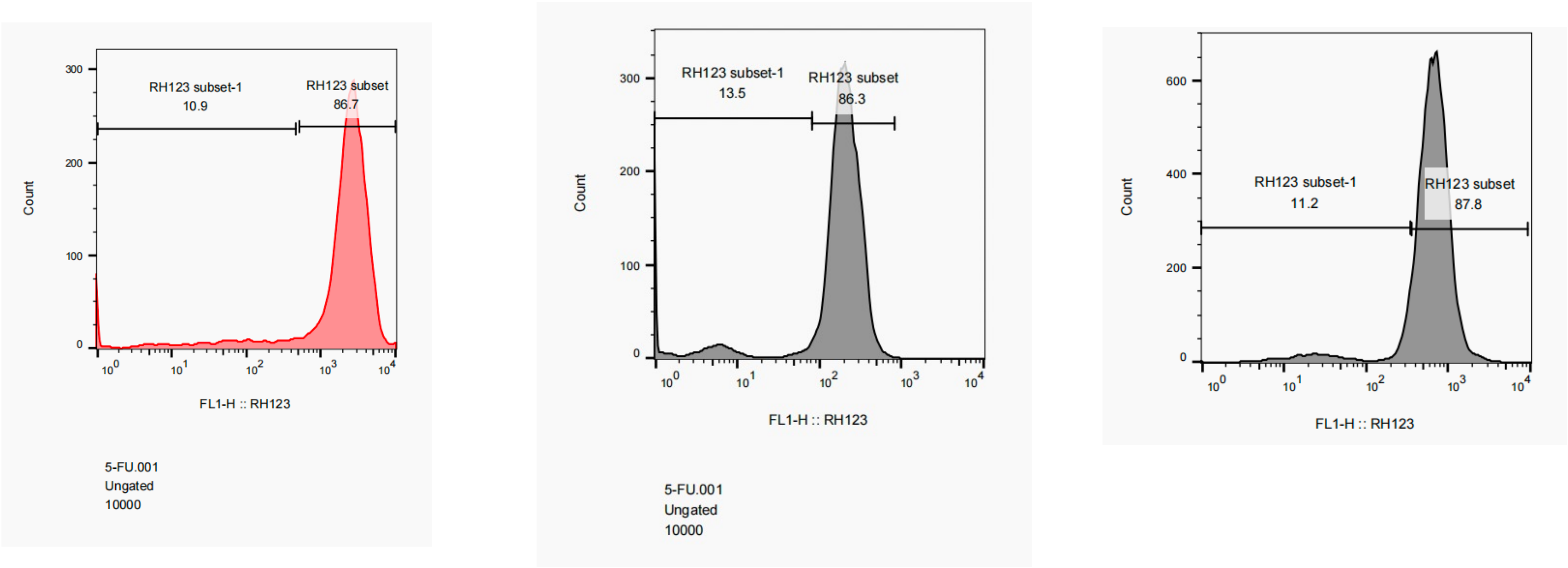

SGC-7901 5-FU

Figure 3E The changes of mitochondrial membrane potential (MMP) in SGC-7901 cells were detected by RH-123 staining after treatment with BI, 5-FU alone or their combination for 12 h

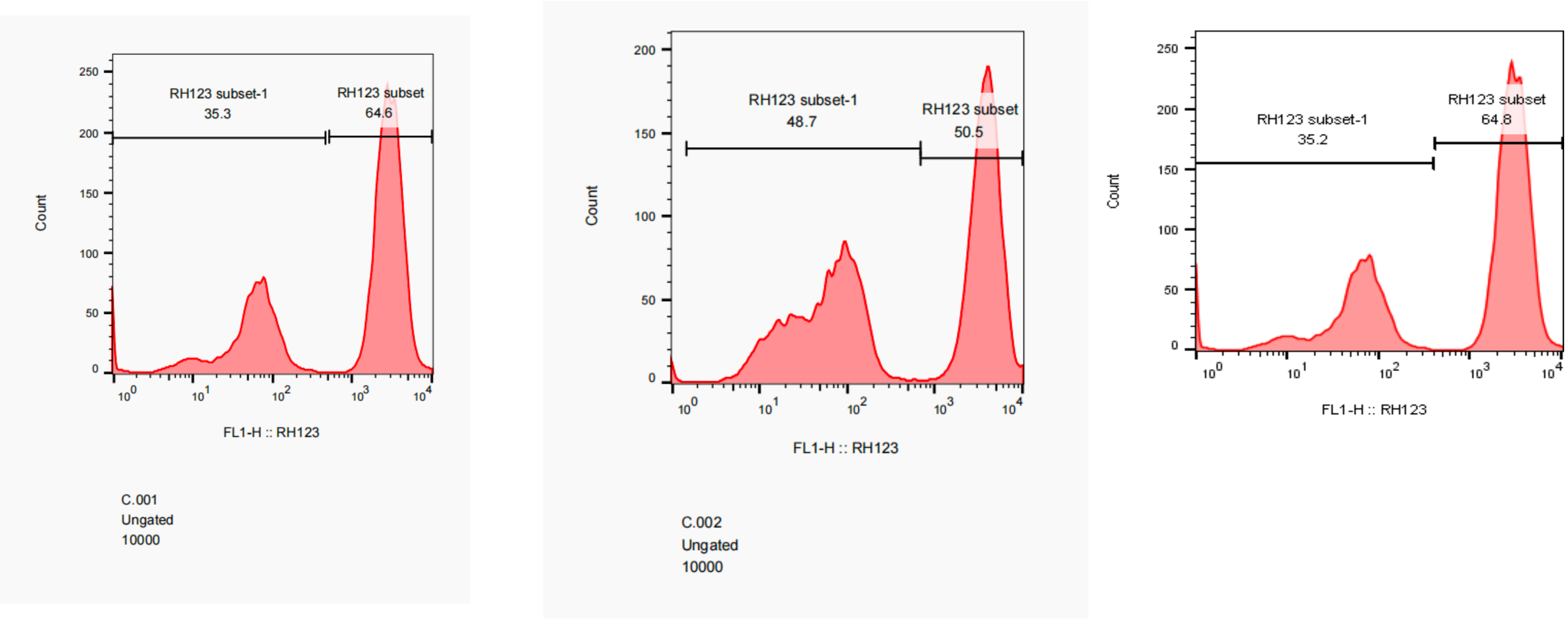

SGC-7901 BI+5-FU

Figure 3E The changes of mitochondrial membrane potential (MMP) in SGC-7901 cells were detected by RH-123 staining after treatment with BI, 5-FU alone or their combination for 12 h

ctrl	BI	5-FU	BI+5-FU
82.5	75.8	87.8	64.6
87.0	88.3	86.7	64.8
86.1	75.3	86.8	50.5

Figure 3F The changes of mitochondrial membrane potential (MMP) in SGC-7901 cells were detected by RH-123 staining after treatment with BI, 5-FU alone or their combination for 24 h

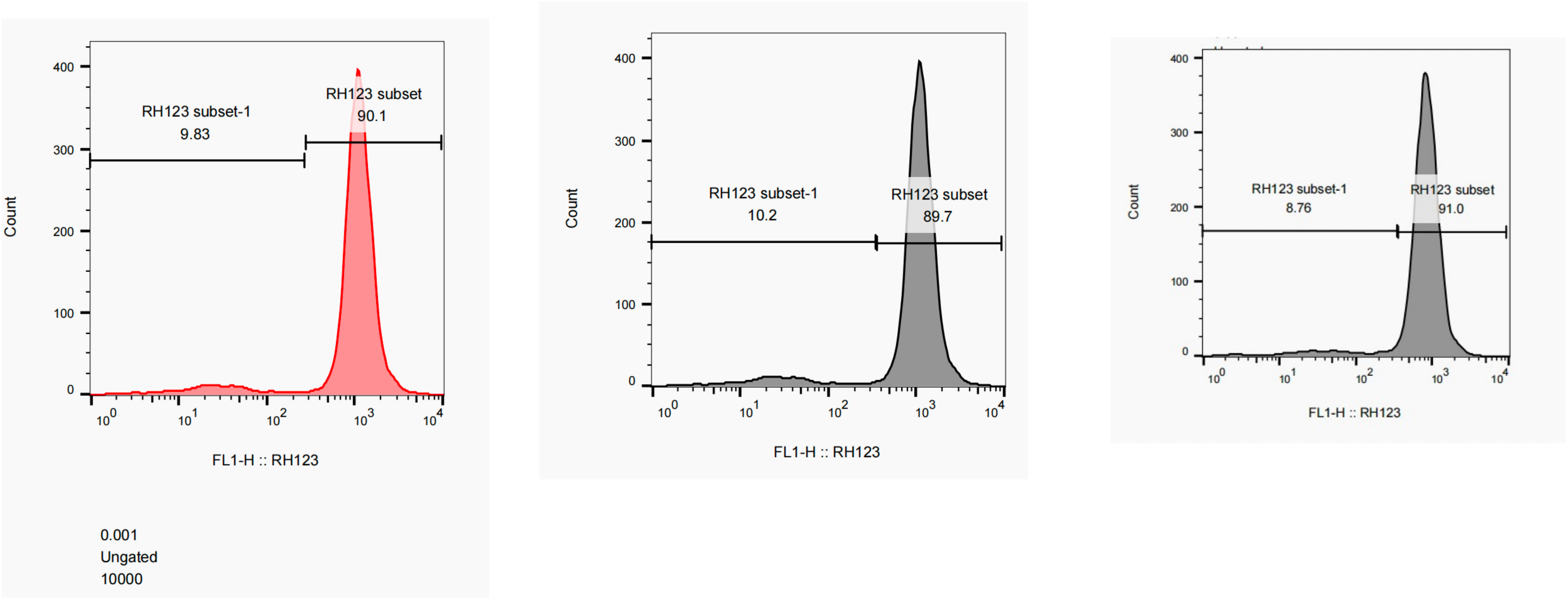

SGC-7901 Ctrl

Figure 3F The changes of mitochondrial membrane potential (MMP) in SGC-7901 cells were detected by RH-123 staining after treatment with BI, 5-FU alone or their combination for 24 h

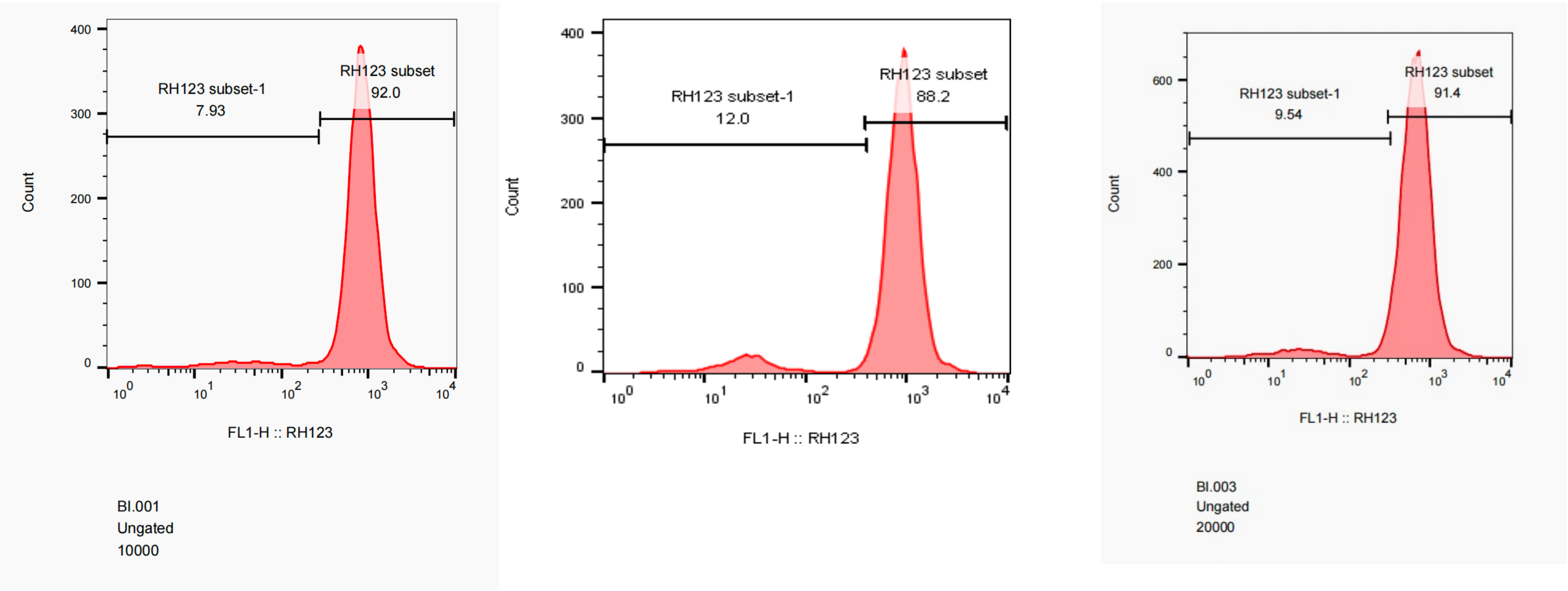

SGC-7901 BI

Figure 3F The changes of mitochondrial membrane potential (MMP) in SGC-7901 cells were detected by RH-123 staining after treatment with BI, 5-FU alone or their combination for 24 h

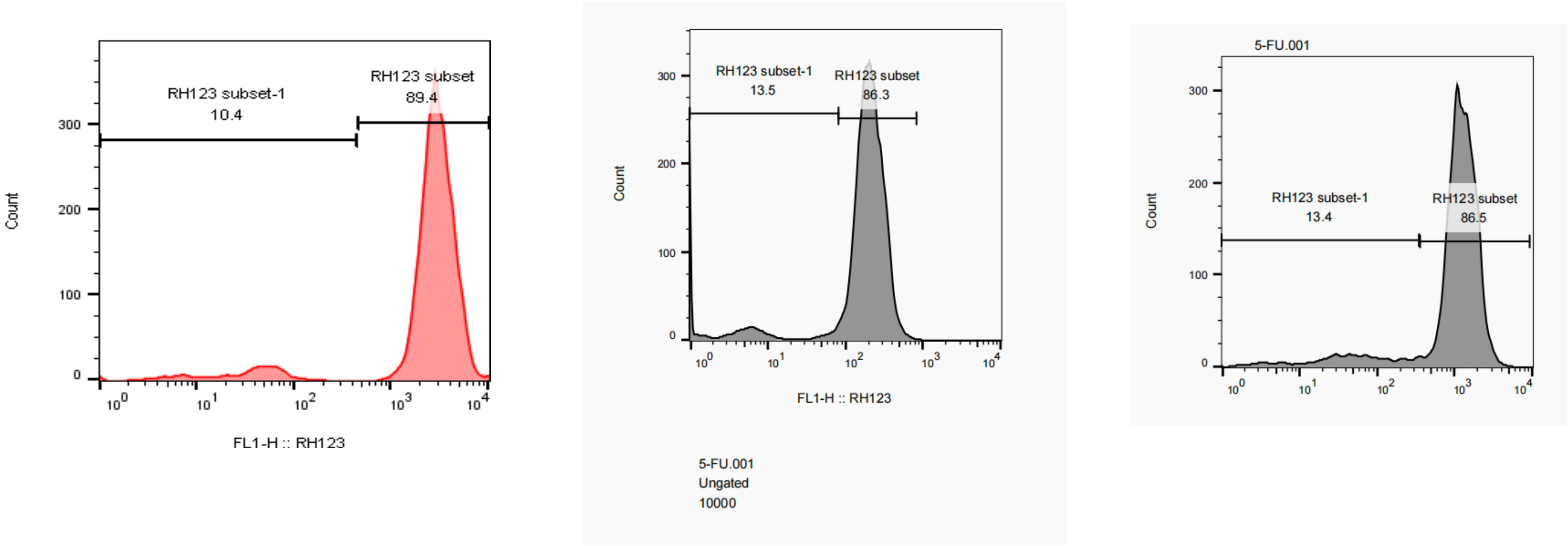

SGC-7901 5-FU

Figure 3F The changes of mitochondrial membrane potential (MMP) in SGC-7901 cells were detected by RH-123 staining after treatment with BI, 5-FU alone or their combination for 24 h

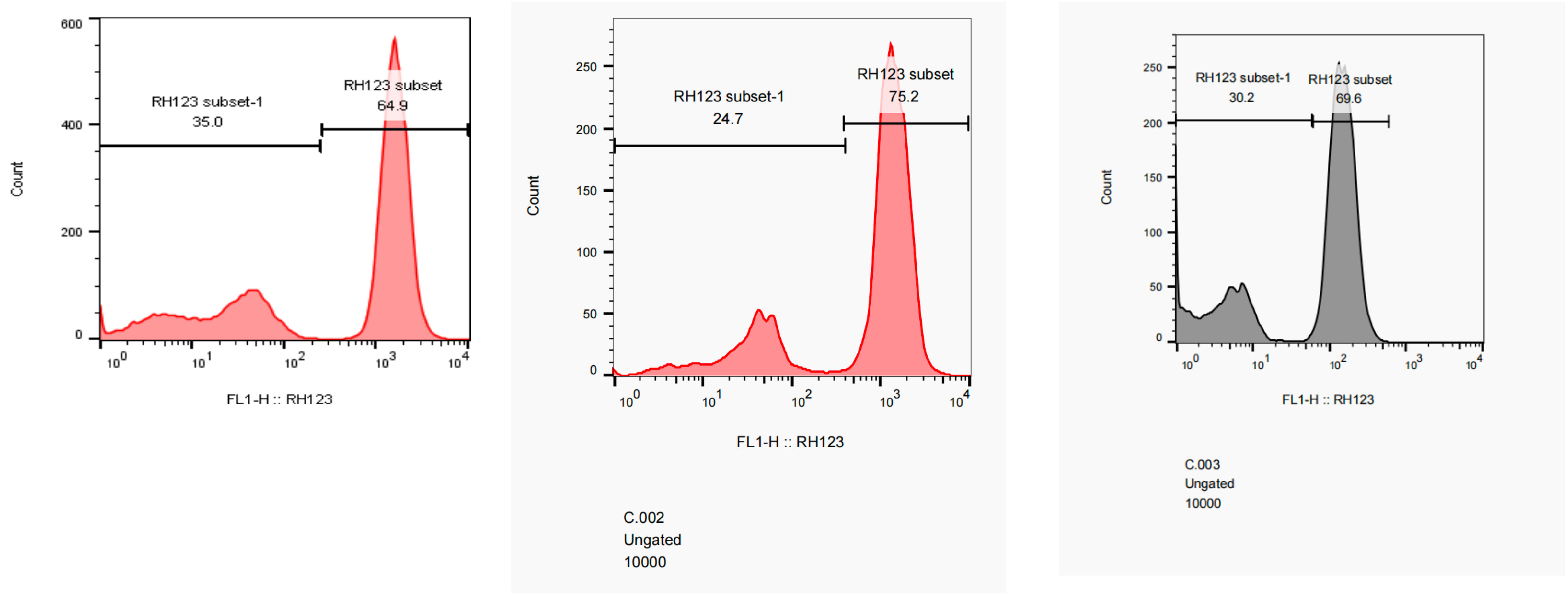

SGC-7901 BI+5-FU

Figure 3F The changes of mitochondrial membrane potential (MMP) in SGC-7901 cells were detected by RH-123 staining after treatment with BI, 5-FU alone or their combination for 24 h

ctrl	BI	5-FU	BI+5-FU
89.7	91.4	86.5	75.2
90.1	92.0	89.4	69.6
91.0	88.2	86.3	64.9
