# Supplementary material for: β-Ionone enhances the inhibitory effects of 5-fluorouracil on the proliferation of gastric adenocarcinoma cells by the GSK-3β signaling pathway
Source: PLoS One. 2024 Sep 6;19(9):e0309014. doi: 10.1371/journal.pone.0309014 (PMC11379261; doi:10.1371/journal.pone.0309014)

Figure 4E HE staining of SGC-7901 cell xenografts

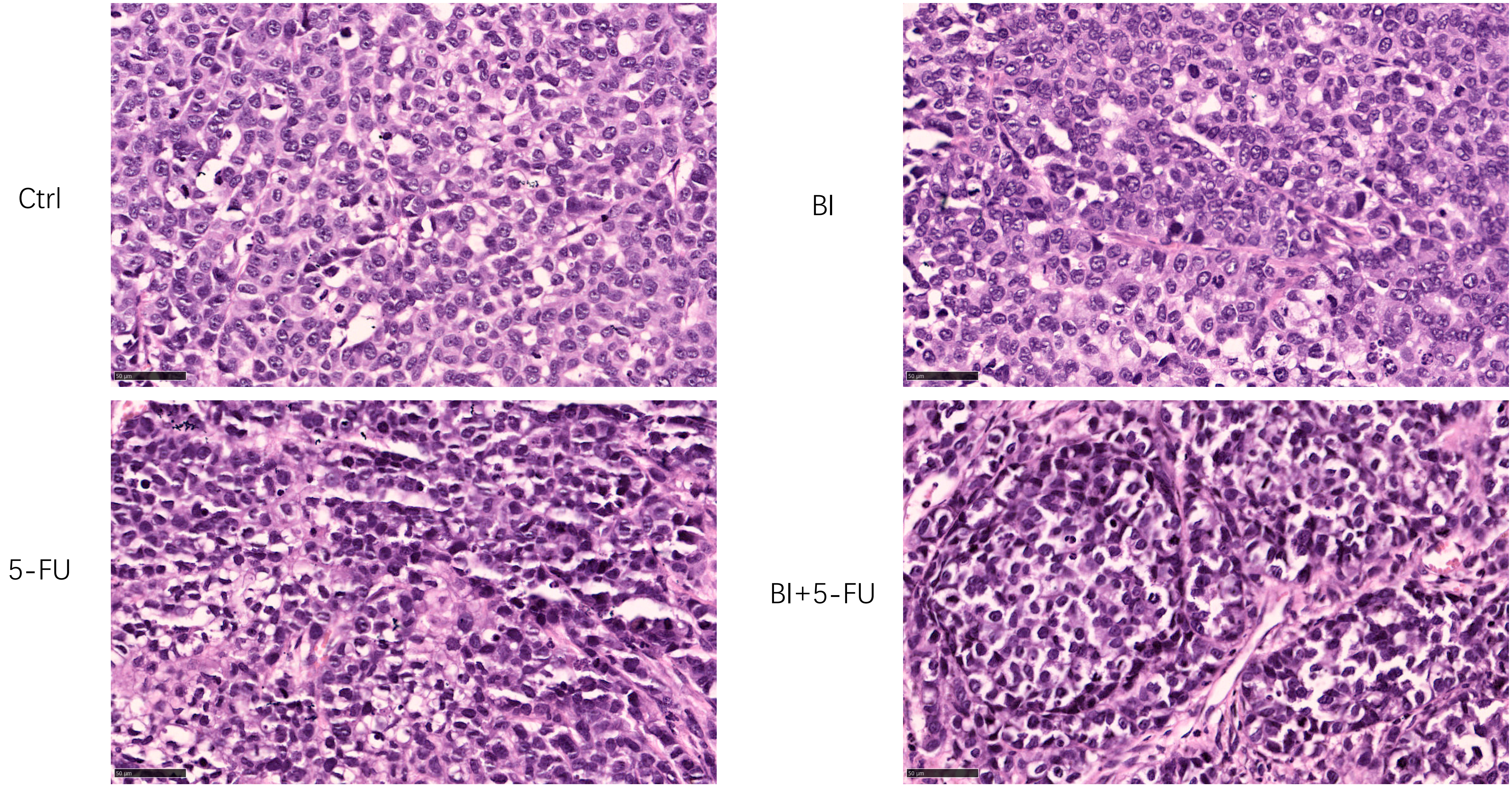

Figure 5A The expression of PCNA was detected in xenografts by IHC

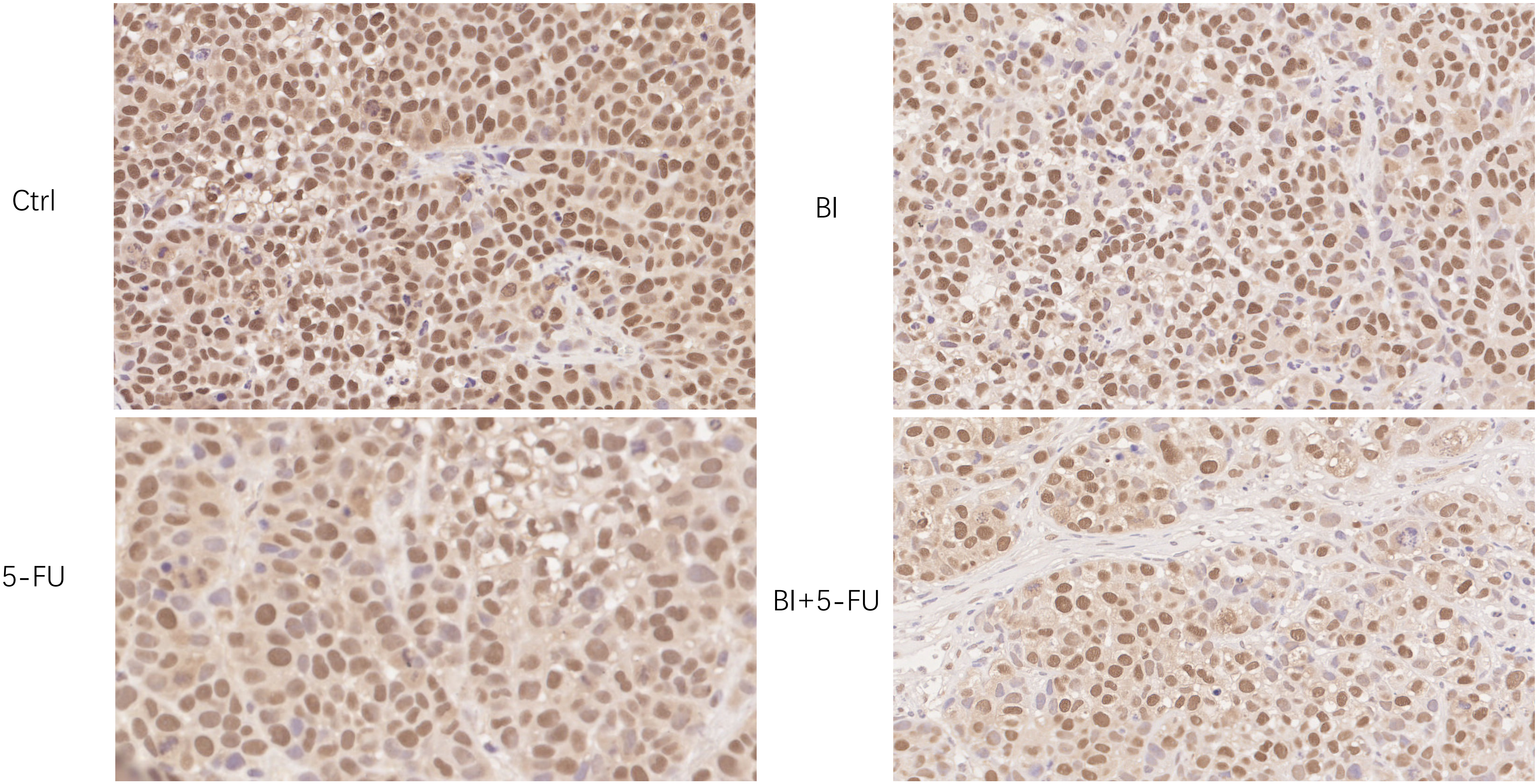

PCNA

0	BI	5-FU	BI+5-FU
302.0	215.0	201.0	124.0
393.0	219.0	211.0	119.0
415.6	250.0	224.2	104.0
410.0	230.0	217.0	128.0

Figure 5D The number of apoptotic cells detected in xenografts by TUNEL

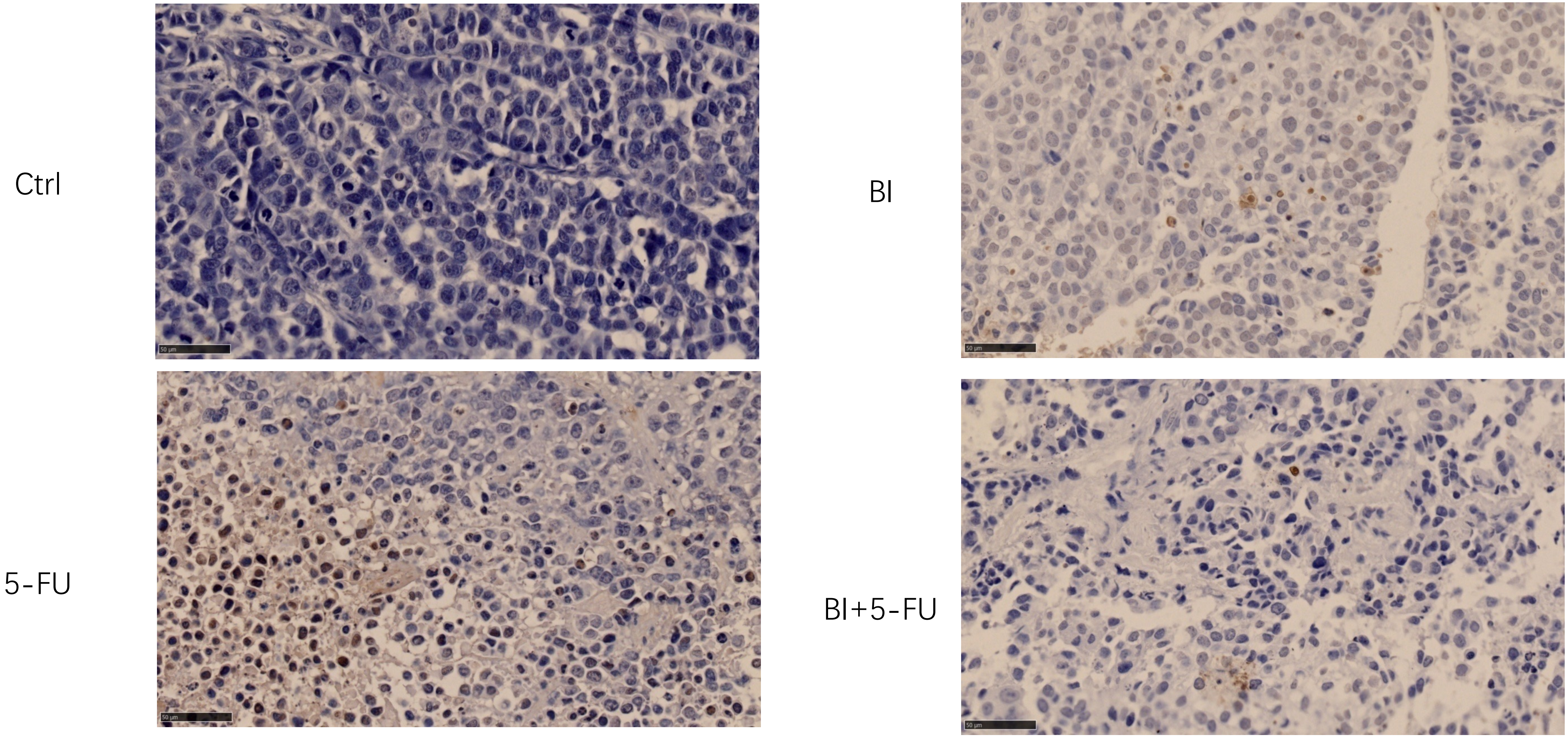

TUNEL 联合	右上	右下	中-3	左上	左下	左上2	左下2	中2	
2020-9-10 18.12.9上	31	32	35	67	31				39.2
2020-9-10 18.12.9下	22	47	24	23	21				27.4
2021-1-12 18.12.9 联合 1左	154	160	212	172	148	166			168.6667
2021-1-12 18.12.9 联合 2左	139	173	183	156	91	151			148.8333
2021-1-12 18.12.9 联合 2右	158	217	140	168	176		154		168.8333
2020-9-10 18.12.9 联合上									
2021-1-12 18.12.9 对照 左上	4	2	2	0	0	0			1.333333
2021-1-12 18.12.9 对照 右上	1	0	1	0	3		2		1.166667
2021-1-12 18.12.9 对照 左下	1	2	1	1	1		0		1
19-10-12 18.12.9对照 上	2	6	1	3	2				2.8
19-10-12 18.12.9对照 上-右	9	7	4	5	8			4	
19-10-12 18.12.9对照 下	1	1	3	2	2			4	2.166667
21-1-12 18.12.9 BI 上	7	6	7	8	15	6			8.166667
21-1-12 18.12.9 BI 下	16	6	13	8	7				10
20-10-12 上	14	10	8	9	9	7			9.5
20-10-12 下	10	22	14	9	9	12			12.66667
19-11-05 BI组 上	12	11	22	8	11				12.8
19-11-05 BI组 下	13	12	15	11	16	10	13	10	12.5
20-9-10 5-FU 18.12.9上	9	9	14	12	8				10.4
20-9-10 5-FU 18.12.9下	15	20	11	15	13				14.8
21-1-12 中	8	17	13	18	19				15
21-1-12 下	10	10	5	23	65				22.6

Figure 5E The expression of Bcl-2 proteins was detected in xenografts by IHC

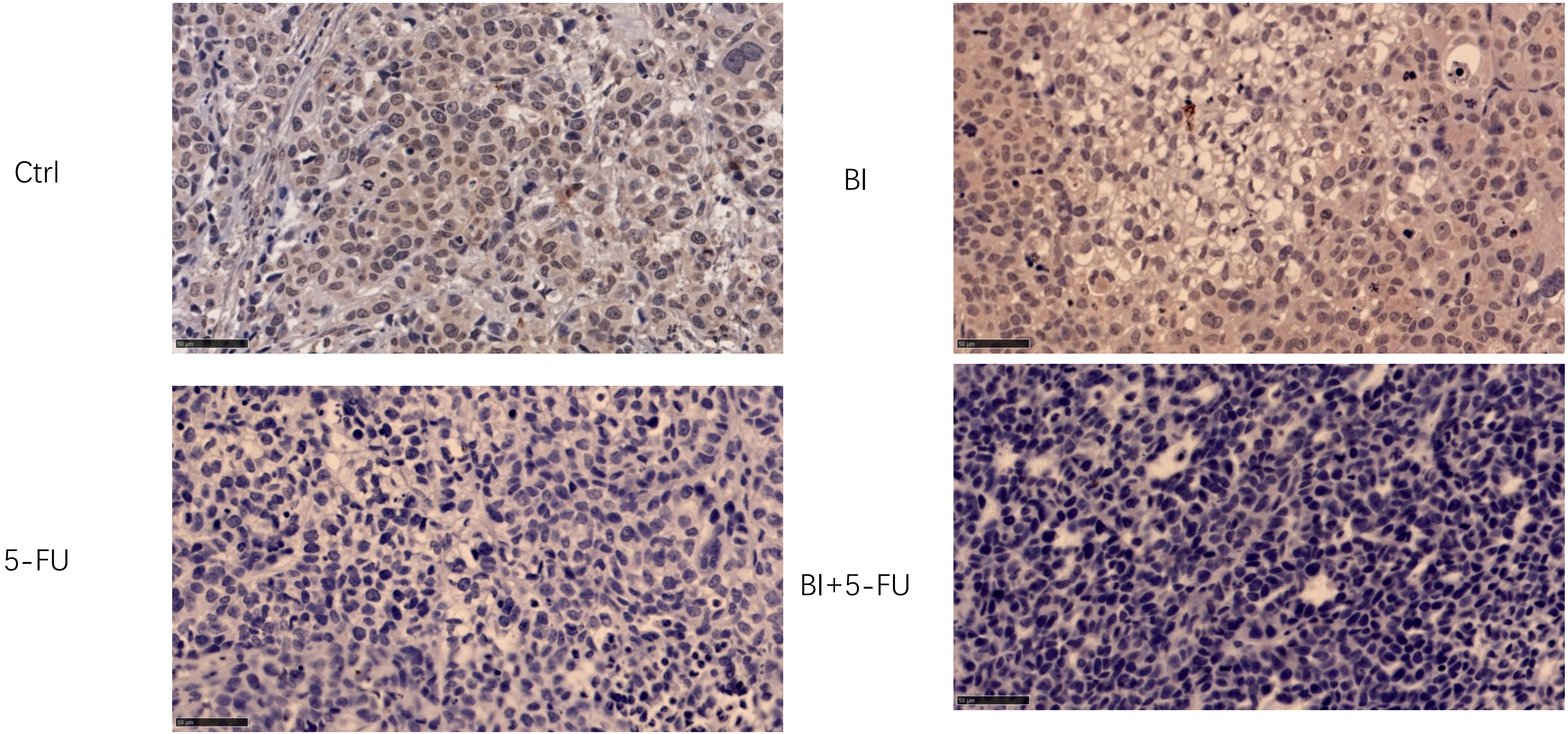

BCL—2	右上	右下	中	左上	左下	中2		
21.4.17 18.10.27 对照 BCL—2上	80	166	129	104	124	160		127.1667
21.4.17 18.10.27 对照 BCL—2右下	141	123	185	208	151	200		168
21.4.17 18.10.27 对照 BCL—2左下	111	115	155	140	93	120		122.3333
21.4.17 18.10.27 BI BCL—2上	39	70	78	85	54			65.2
21.4.17 18.10.27 BI BCL—2右下	29	40	28	63	66			45.2
21.4.17 18.10.27 BI BCL—2左下	82	90	140	41	64	62		79.83333
21.4.19 18.12.9 bcl—2 5—FU 左	8	11	30	21	8			15.6
21.4.19 18.12.9 bcl—2 5—FU 左下	22	14	10	24	20	12		17
21.6.30 18.12.9 bcl—2 5—FU 右	34	17	21	33	38			28.6
21.6.30 18.12.9 bcl—2 5—FU 左	47	16	31	58	40			38.4
21.4.17 18.10.27 联合 BCL—2上	0	0	1	0	0			0.2
21.4.17 18.10.27 联合 BCL—2右下	1	1	0	0	0			0.4
21.4.17 18.10.27联合 BCL—2左下	0	0	1	0	0			0.2
21.6.24 18.12.9 联合 Bcl—2左	0	3	1	0	2			1.2

Figure 5F The expression of Bax proteins was detected in xenografts by IHC

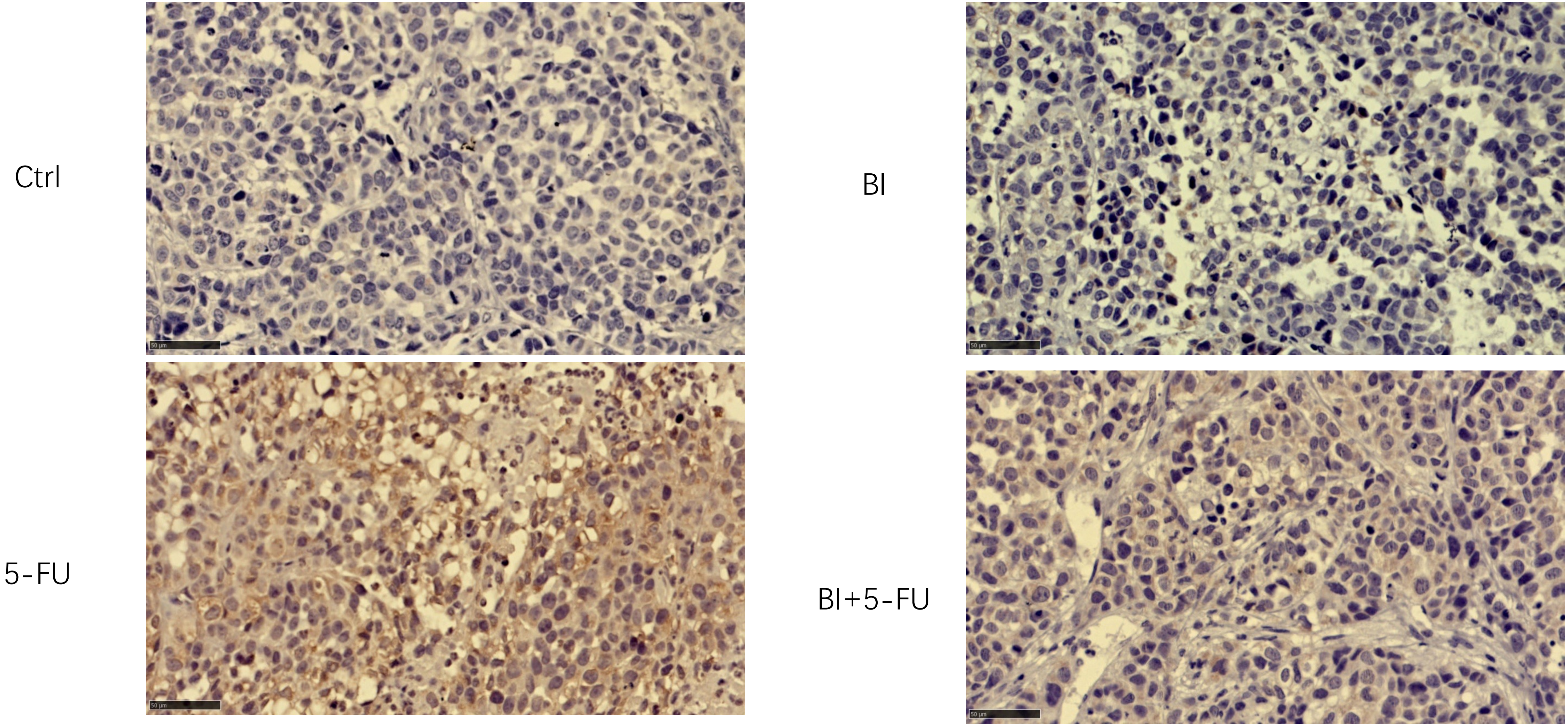

BAX	右上	右下	中	左上	左下	左上-2		
21.4.20 18.12.9 对照bax上	0	0	4	0	0			0.8
21.4.20 18.12.9 对照bax下	3	1	2	3	1			2
21.4.20 18.12.9 对照bax右上	0	0	0	1	0			0.2
21.6.24 18.12.9 对照 bax右	0	2	0	0	0			0.4
21.6.24 18.12.9 对照 bax中	0	0	1	0	0			0.2
21.6.24 18.12.9 对照 bax左	1	0	0	0	0			0.2
21.4.23 18.12.9 联合 bax上	54	57	114	123	100	76		89.6
21.4.23 18.12.9 联合 bax下	58	80	78	144	129	116		97.8
21.6.24 18.12.9 联合 bax右	130	178	141	160	167			155.2
21.6.24 18.12.9 联合 bax左	165	172	170	149	204			172
21.4.20 18.12.9 BI bax上	33	35	28	36	16	25		29.6
21.4.20 18.12.9 BI bax右	13	6	20	18	14	16		14.2
21.4.20 18.12.9 BI bax左上	10	12	18	16	21			15.4
21.4.20 18.12.9 5-FU bax 右上	11	6	10	10	15	9		10.4
21.4.20 18.12.9 5-FU bax 左上	6	11	7	16	20			12
21.4.20 18.12.9 5-FU bax 左下	12	9	13	17	7	14		11.6

Figure 5G The expression of Cleaved caspase-3 proteins was detected in xenografts by IHC

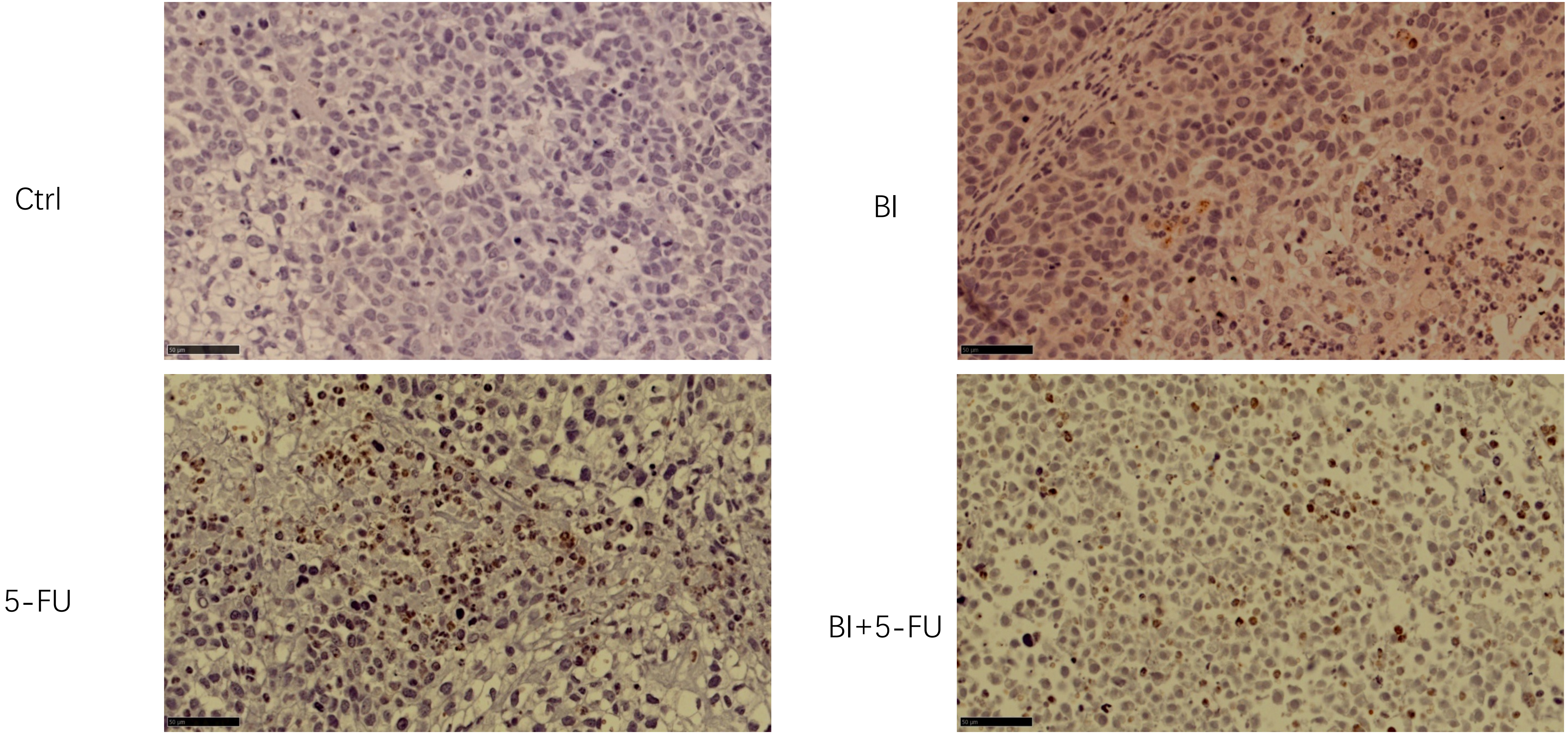

caspase3	右上	右下	中	左上	左下	中2		
18.12.9 19.9.27caspase3 联合 1 右	95	94	124	113	67	65		93
18.12.9 19.9.27caspase3 联合 1 左	71		62	78	66	64		68.2
18.12.9 19.9.27caspase3 联合 右上	95	85	88	89	81	71		84.83333
18.12.9 21.6.24caspase3 联合 右	35	31	39	42	28			35
18.12.9 21.6.24caspase3 联合 左	27	25	22	15	21			22
19.9.22 18.11.21 4组 BI 2 caspase3	5	7	12	12	16	11		10.5
19.9.22 18.11.21 4组 BI 2 caspase3	6	7	7	7	9	8		7.333333
19.9.22 18.11.21 BI caspase3 右	14	6	8	11	5			8.8
19.9.22 18.11.21 BI caspase3 左2	5	6	14	5	4	7		6.833333
19.9.22 18.11.21 BI caspase3 左上	9	5	10	8				8
21.4.23 18.12.9 5-FU caspase3上	10	11	15	7	9			10.4
21.4.23 18.12.9 5-FU caspase3下	8	6	8	7	6	6		6.833333
21-6-24 18-12-9 5-FUcaspase3右	18	16	20	13	8			15
21-6-24 18-12-9 5-FUcaspase3左	16	11	11	15		9		12.4
19.9.22 18.11.21 对照 caspase3上	0	0	1	0	2	0		0.5
19.9.22 18.11.21 对照 caspase3右	2	0	0	0	1	0		0.5
19.9.22 18.11.21 对照 caspase3中	0	0	1	0	0	0		0.166667
21-4-23 18.11.21 对照caspase3 上	0	1	0	0	0	0		0.166667
21-4-23 18.11.21 对照caspase3 右上	0	0	1	0	0	0		0.166667

Figure 6C The changes of pGSK-3β protein expression in SGC-7901 cells by immunofluorescence assay

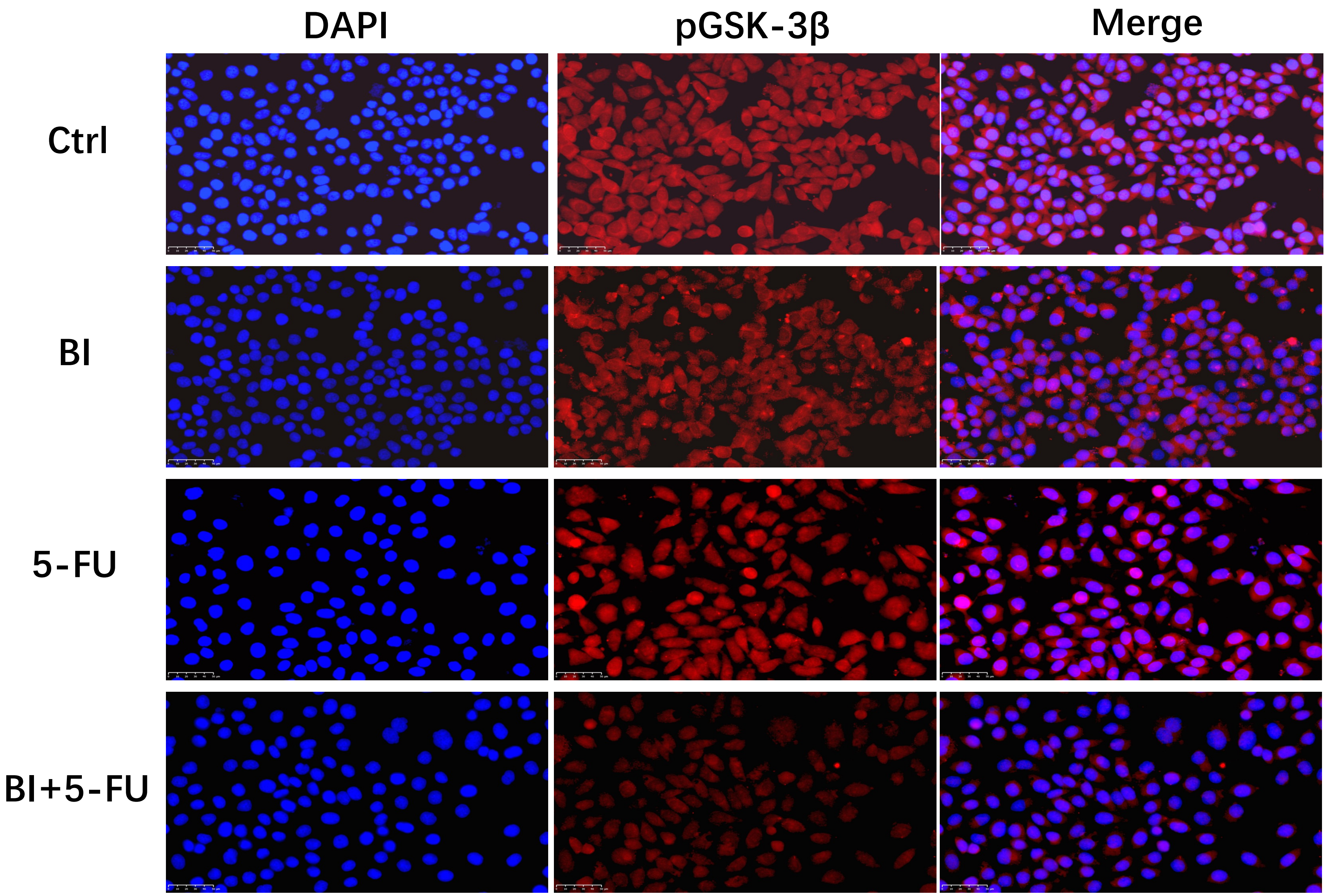

Supplement: S2 Data — (PDF) [file pone.0309014.s002.pdf]
