# Supplementary material for: β-Ionone enhances the inhibitory effects of 5-fluorouracil on the proliferation of gastric adenocarcinoma cells by the GSK-3β signaling pathway
Source: PLoS One. 2024 Sep 6;19(9):e0309014. doi: 10.1371/journal.pone.0309014 (PMC11379261; doi:10.1371/journal.pone.0309014)

Figure 2E WB assessed the expression of PCNA after treatment of SGC-7901 cells with BI and 5-FU alone or in combination for 72h.

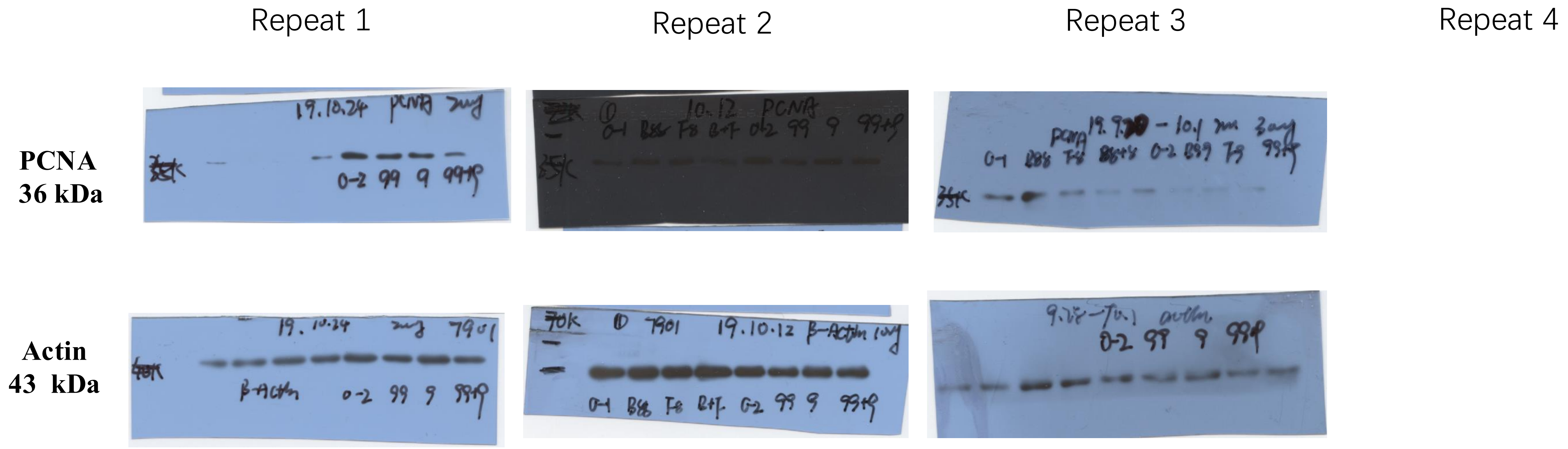

Figure 2F WB assessed the expression of Cyclin D1 and CDK4 after treatment of SGC-7901 cells with BI and 5-FU alone or in combination for 72h.

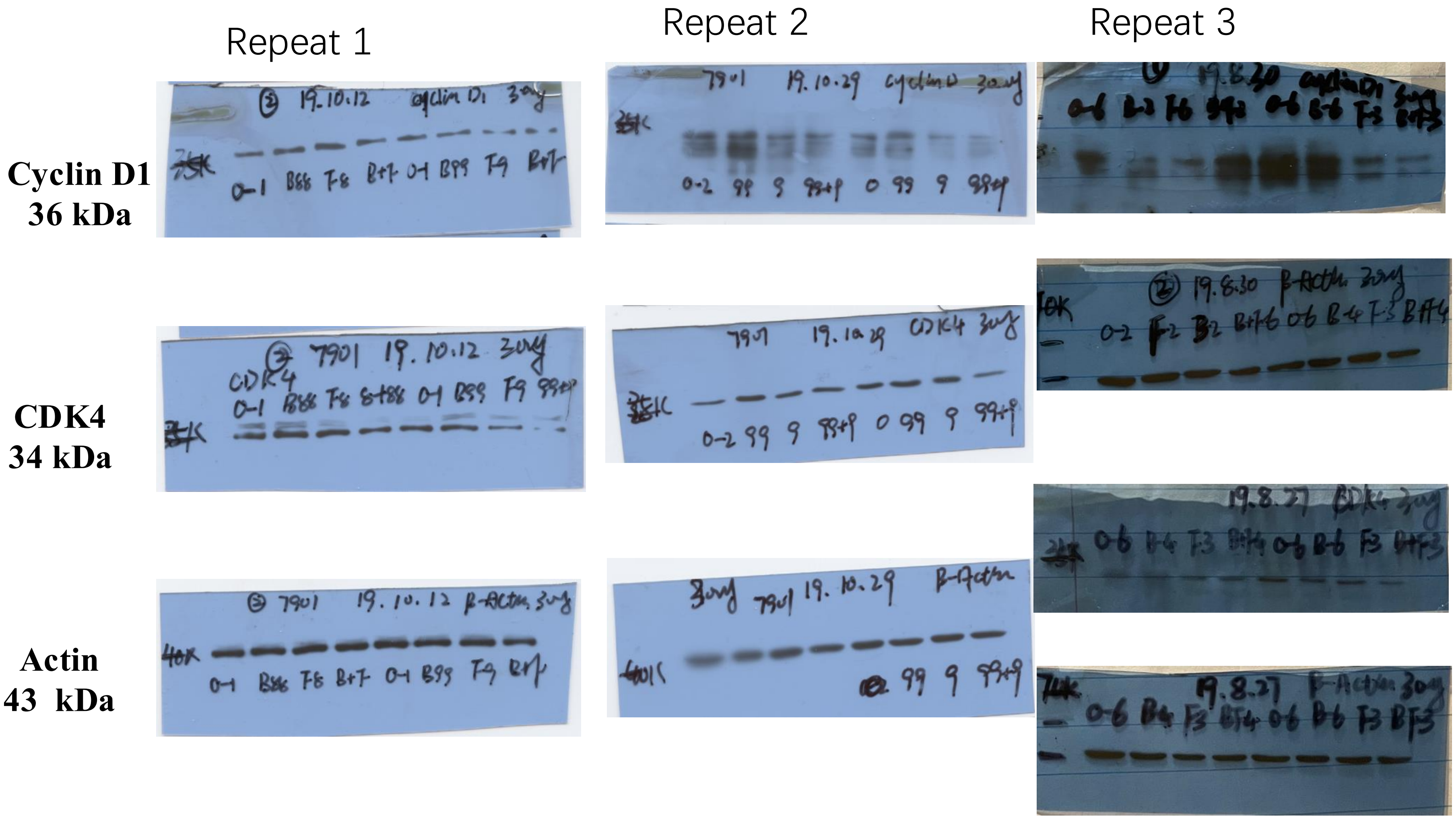

Figure 5B The expression of PCNA was detected in xenografts by Western blot

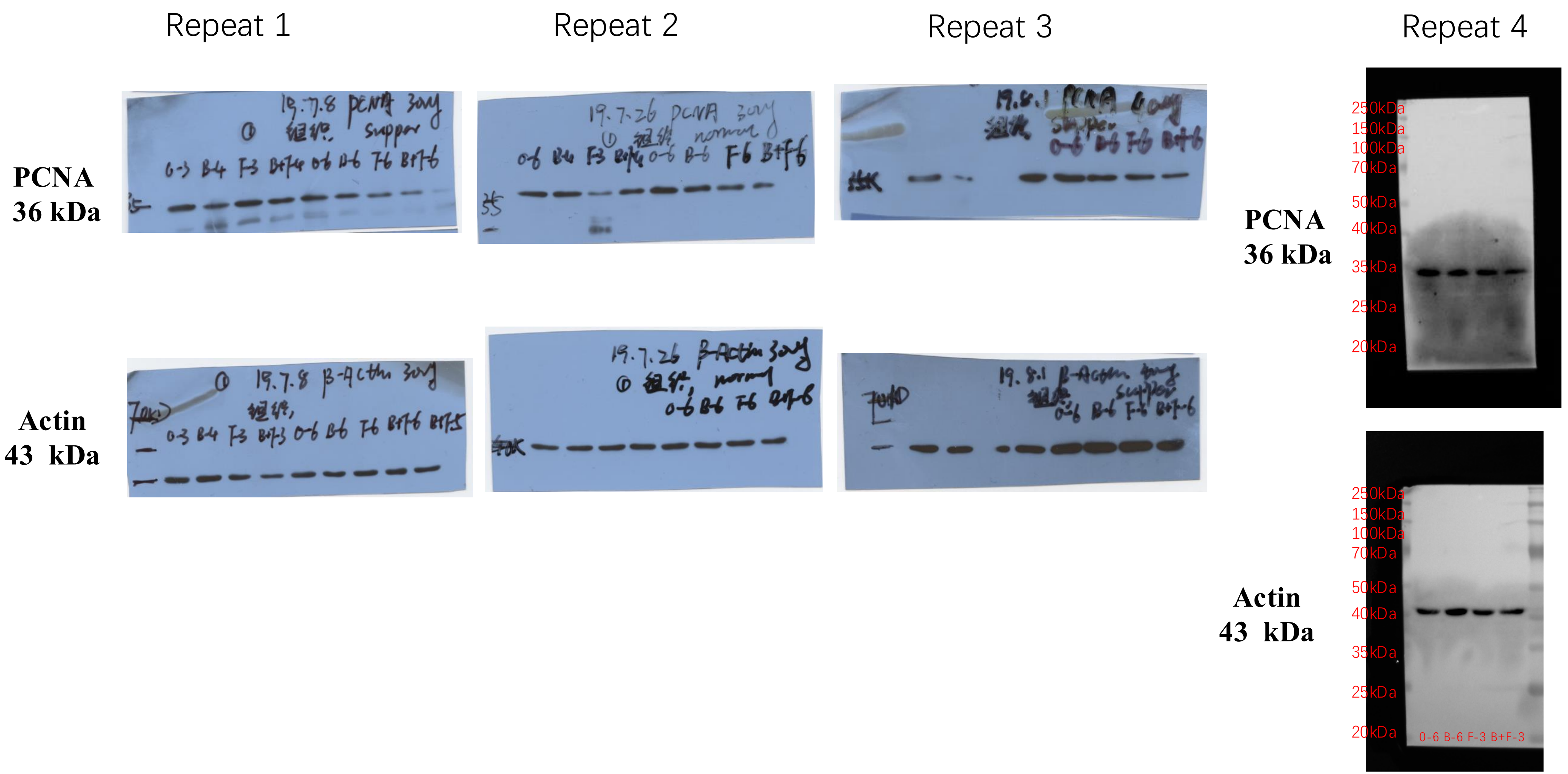

Figure 5C The expression of Cyclin D1 and CDK4 in xenografts

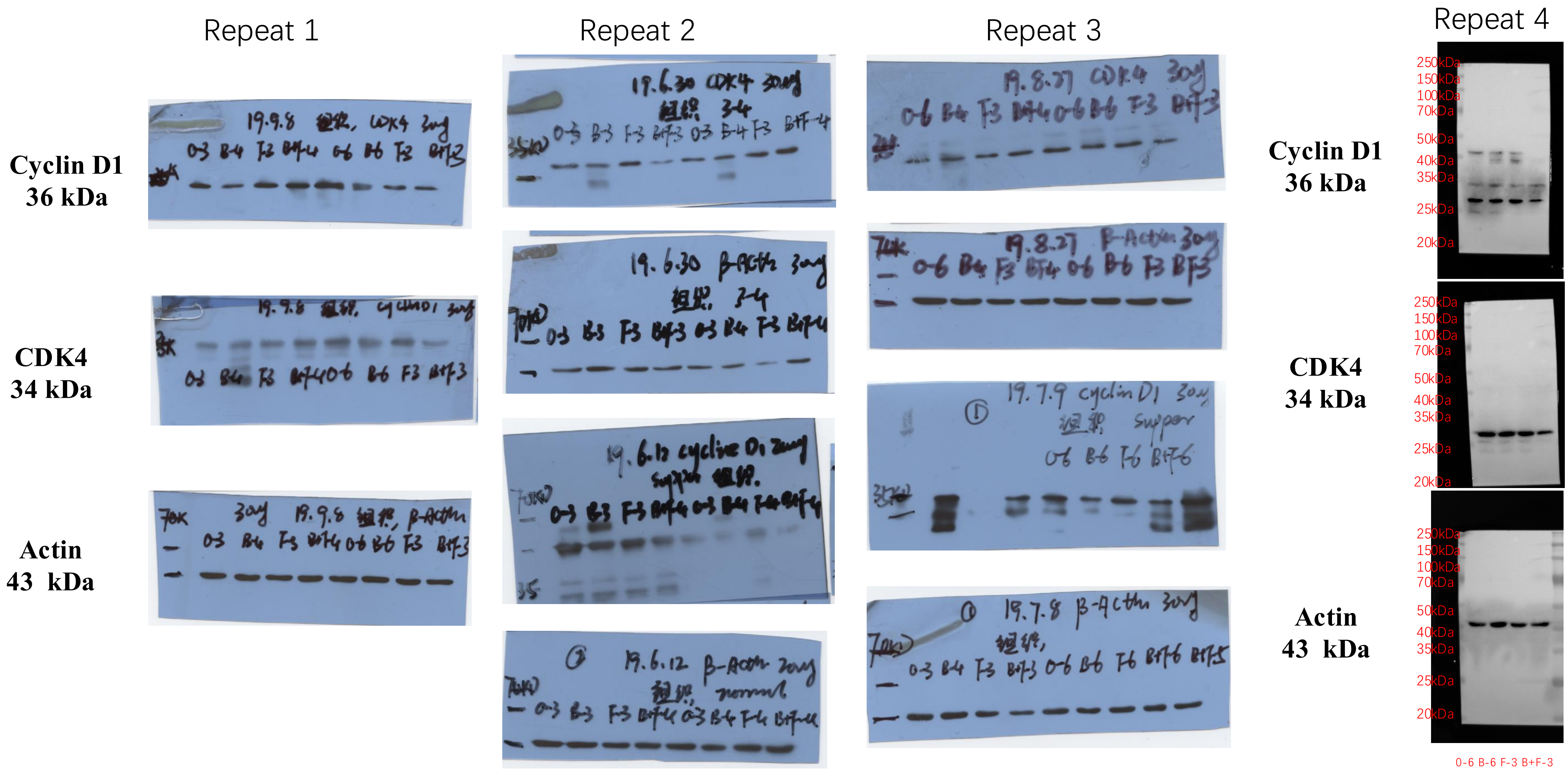

Figure 6A The expression of pGSK-3β protein was examined in SGC-7901 cells treated with different concentrations of BI by Western blot

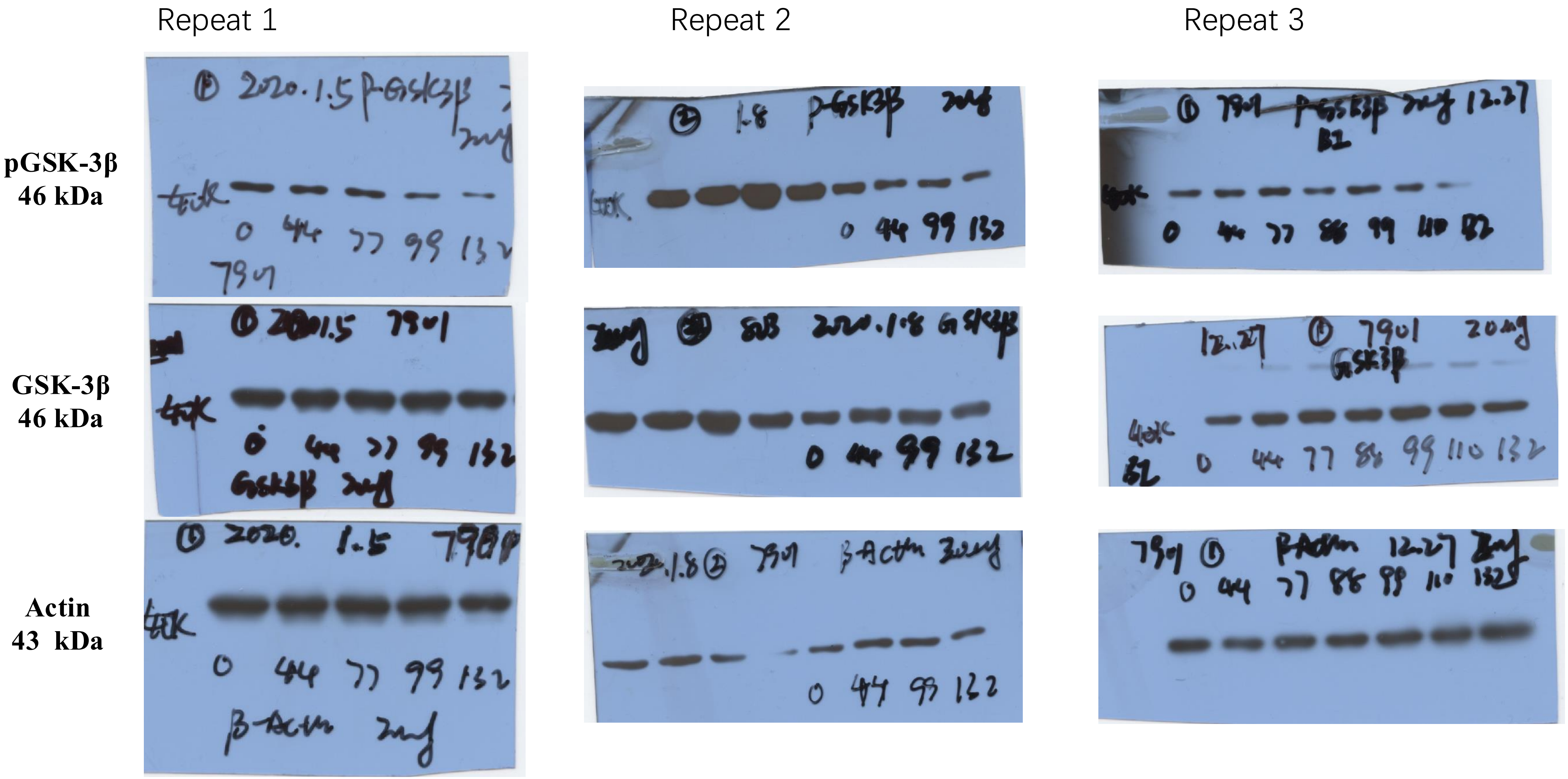

Figure 6B The expression of pGSK-3β protein was examined in SGC-7901 cells treated with BI and 5-FU alone and in their combination

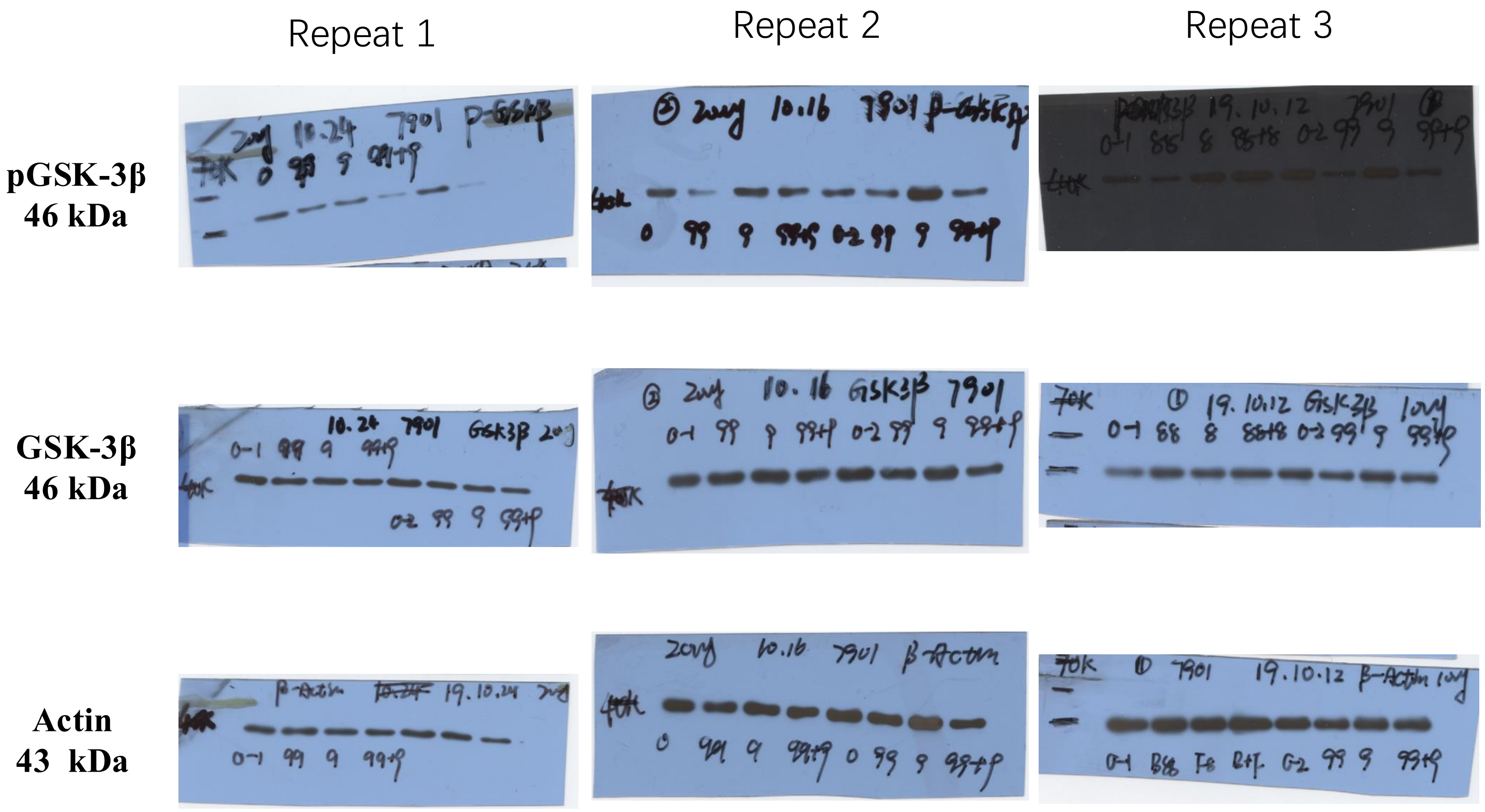

Figure 6D The expression of pGSK-3β protein in SGC-7901 cell xenografts

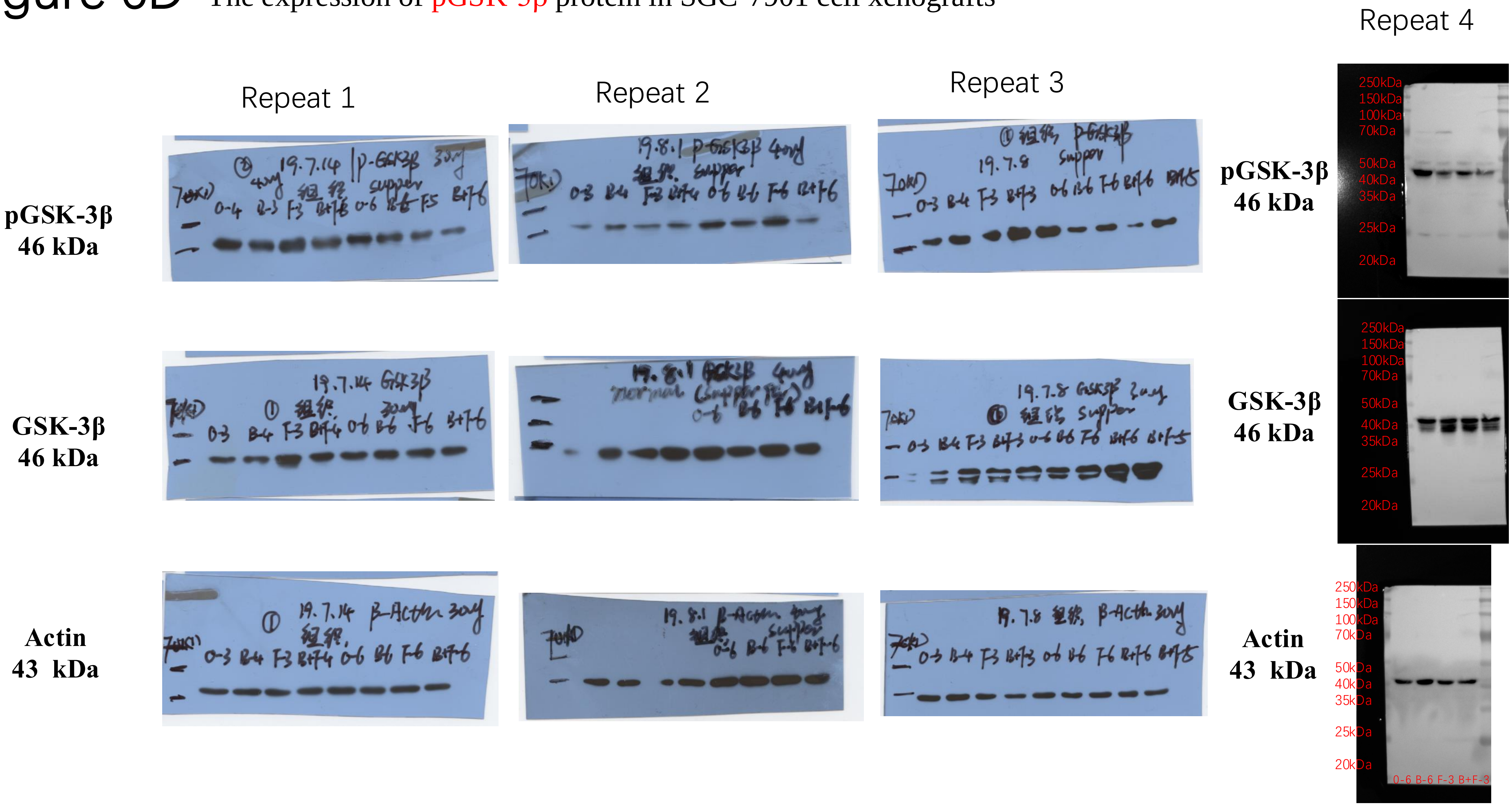

Supplement: S3 Data — (PDF) [file pone.0309014.s003.pdf]
